# Association of short-term ambient environmental exposures with suicide and drug overdose deaths among U.S. Veterans

**DOI:** 10.1093/aje/kwag099

**Published:** 2026-05-12

**Authors:** Alina Peluso, Dirga Lamichhane, James A. VanDerslice, Jeremy Logan, Anuj Kapadia, Jodie A. Trafton, Amanda V. Bakian

**Affiliations:** 1 Oak Ridge National Laboratory, Oak Ridge, TN 37830, United States; 2 University of Utah, Salt Lake City, UT 84112, United States; 3 U.S. Department of Veterans Affairs, Veterans Health Administration, Palo Alto, CA 94025, United States

**Keywords:** built environment, case-crossover study, suicide, drug overdose, air pollution, meteorological factors, short-term exposure, risk assessment

## Abstract

This study examined associations between short-term ambient environmental exposures and suicide (*n* = 3210) and overdose mortality (*n* = 4293; 2226 opioid-related) among U.S. Veterans from 2018 to 2019. Daily exposure to 24-hour maximum temperature, average atmospheric pressure, average fine particulate matter (PM_2.5_), 1-hour maximum nitrogen dioxide (NO_2_), and 8-hour maximum ozone (O_3_) was assessed at the decedent’s county of residence on the day of death and up to 6 days prior. A national bidirectional, time-stratified case-crossover design was applied. Conditional logistic regression models estimated associations between each exposure and suicide or overdose deaths, overall, and stratified by season, region, elevation, and urbanicity. Over lag days 0-1, an interquartile range increase in maximum temperature was associated with increased suicide (19%) and overdose (27%) mortality, with stronger summer effects for suicide (55%) and overdose (71%). In winter, interquartile range increases in atmospheric pressure, PM_2.5_, and NO_2_ were associated with 104%, 15%, and 19% increases in suicide mortality. Maximum temperature was associated with a 22% increase in suicide risk in metropolitan areas and 57% in the Western U.S., while NO_2_ was associated with a 26% increase in overdose mortality in nonmetropolitan areas. Findings suggest environmental stressors contribute to suicide and overdose mortality among Veterans, supporting environmentally informed prevention efforts.

## Introduction

Rates of suicide, drug overdose, and alcohol-related deaths have been on the rise in the United States, reflecting a complex public health crisis influenced by structural inequities, economic challenges, and behavioral risk factors.^1–3^ While long-term risk of suicide and drug overdose is shaped by persistent social and psychological stressors—such as psychiatric illness, posttraumatic stress disorder, and social isolation in rural settings^4,5^—there is increasing interest in the role of acute environmental exposures. Short-term fluctuations in air quality and temperature may act as proximal triggers for suicide and overdose in vulnerable individuals.^6^ Given the narrow window for effective intervention preceding these deaths, a clearer understanding of how transient environmental factors contribute to early mortality is urgently needed across diverse U.S. settings.

Epidemiological studies have linked short-term fluctuations in air pollution and weather conditions to an increased risk of suicide death,^7,8^ and to a lesser extent, overdose mortality.^9–11^ Mechanistic evidence suggests that acute exposure to air pollutants may provoke neuroinflammation and oxidative stress, disrupt serotonergic signaling, and alter the regulation of mood and impulse control.^12, 13^ Similarly, rapid changes in ambient temperature can activate the hypothalamic-pituitary-adrenal (HPA) axis, increasing physiological stress while simultaneously exacerbating cardiovascular and respiratory vulnerabilities—factors known to elevate the risk of fatal overdose.^9,14–16^ Additionally, changes in atmospheric pressure may impact the autonomic nervous system, influencing stress responses and emotional regulation.^17^

Seasonal factors are also known to influence the risk of suicide and overdose.^18^ In temperate regions, including the United States, suicide rates historically peak in late spring and early summer,^19–21^ and are associated with seasonal environmental exposures, such as increased sunshine duration and solar radiation.^22–24^ In contrast, preliminary research reports higher rates of opioid-related mortality outcomes in the winter.^25^ A case-crossover study in Connecticut and Rhode Island found that lower average temperatures (0°C vs 11°C) in the 3 to 7 days before death were associated with up to a 30% increase in the odds of opioid overdose death,^10^ with the authors suggesting that concurrent exposure to cold and opioids may impair thermoregulation and respiratory function, thereby increasing mortality risk. In combination, evidence of seasonal variation in suicide and opioid-related mortality, along with seasonal variability in environmental exposures, supports examining exposure-outcome associations by season. Environmental exposures also vary geographically across the United States and are correlated with factors, such as higher elevation, which has been linked to elevated suicide risk.^26–29^ Overdose mortality rates similarly exhibit substantial geographic variation across the United States.^30^ Although geographically varying factors—such as drug supply instability, economic distress, rurality,^31^ healthcare access,^32^ demographic composition,^33^ and social context—may partly explain spatial differences in overdose mortality, rates also correlate with geographic features characterized by distinct air pollution and weather patterns, such as metropolitan^34^ and mountain regions.^35^

Most research on the association between environmental exposures and suicide or overdose mortality has focused on civilian populations, with limited investigation among U.S. Veterans.^36,37^ Yet Veterans experience disproportionately high rates of suicide^4,38^ and drug overdose,^39^ partly due to the elevated mental health risks associated with the physical and psychological demands of military service.^38^ As such, the extent to which findings from civilian studies apply to Veterans remains unclear. While the U.S. Department of Veterans Affairs has prioritized suicide and overdose prevention through various proactive initiatives, understanding the role of environmental exposures may further strengthen prevention efforts by helping to identify acute external stressors that could contribute to periods of heightened vulnerability.

To address gaps in knowledge, we conducted the first national study examining the relationship between short-term exposures to daily measures of fine particulate matter (PM_2.5_), nitrogen dioxide (NO_2_), ozone (O_3_), maximum temperature, and air pressure with the risk of suicide, drug overdose, and opioid overdose in U.S. Veterans. Using a case-crossover design and Veteran Health Administration (VHA^(1)^) data, we also explored variation of these associations by season, rurality, elevation, and region. This focus on acute exposures aims to identify environmental triggers, which may help guide targeted intervention for high-risk Veterans.

## Methods

### Participants

The study included Veterans eligible for VHA care who died between January 1, 2018, and December 31, 2019, from suicide (ICD-10 codes U03, X60-X84, Y87.0), drug overdose (X40-X44, X60-X64, X85, Y10-Y14), or opioid overdose (T40.0-T40.4, T40.6, listed as contributing causes). Overdose deaths included intentional, unintentional, and undetermined manners. These outcome categories were not mutually exclusive; decedents could be classified under more than one category. For example, a death coded as an intentional opioid overdose (eg., X64) was classified as both a suicide and an opioid overdose. Each outcome was analyzed independently in separate models. Deaths were identified through the VA/Department of Defense Mortality Data Repository. Residential location at time of death was assigned using ZIP code-linked Federal Information Processing Standards (FIPS) county codes^(2)^. Deaths occurred across 1691 counties for suicide, 1032 for drug overdose, and 749 for opioid overdose.

### Environmental exposure and geographic factors

Meteorological data, including daily precipitation (mm/day), maximum ambient temperature (°C), and shortwave radiation (W/m^2^), were obtained from the Daymet 1-km gridded dataset (version 4 R1) provided by NASA’s Oak Ridge National Laboratory Distributed Active Archive Center^(3)^. Mean daily sea-level atmospheric pressure (Pa) was sourced from the ERA5 reanalysis product maintained by the European Centre for Medium-Range Weather Forecasts (ECMWF) ^(4)^. Estimates of daily air pollution—24-hour average PM_2.5_ (μg/m^3^), 1-hour maximum NO_2_ (ppb), and 8-hour maximum O_3_ (ppb)—were derived from fused model-observation outputs generated by the U.S. EPA Community Multiscale Air Quality (CMAQ^(5)^) system^(6)^.

County-level exposure values were calculated by averaging gridded values across populated areas within each county, using WorldPop 100-m resolution raster data to define populated zones^(7)^.

Elevation was obtained from the USGS Global Multi-resolution Terrain Elevation Data 2010 (GMTED2010^(8)^) on a 15-arcsecond 450-m grid.^40^ Rurality was characterized using the 2010 Rural–Urban Commuting Area (RUCA^(9)^) codes from the U.S. Department of Agriculture, based on population, urbanization, and commuting patterns. Each county’s rurality was classified based on the proportion of metropolitan (codes 1-3) vs nonmetropolitan (codes 4-10) U.S. Census tracts. U.S. Census region classification followed standard definitions from the Department of Commerce^(10)^.

### Statistical analysis

We used a bi-directional, time-stratified case-crossover design to assess whether short-term exposure to environmental factors was associated with suicide, drug overdose, and opioid overdose deaths among U.S. Veterans. This within-person design controls for time-invariant factors by comparing each participant exposure during case and control periods.

For each decedent, the exposure window was defined as the day of death (lag 0) and each of the six preceding days (lags 1-6), capturing a total of seven days of potential exposure. Referent (control) days were selected bi-directionally, matched on the day of the week within the same calendar month and year as the case day, to account for seasonal, monthly, and weekday-specific exposure variability. Each decedent contributed three to four control days to the analysis.

We used conditional logistic regression models to estimate odds ratios (ORs) and 95% confidence intervals (CIs) to measure the association between environmental exposures and the odds of suicide, drug overdose, or opioid overdose. We examined the relationships with single-day lag exposures (eg, lag 0, lag 1, …, lag 6) and cumulative exposures (eg, average exposures across two or more days; lag 0-1, lag 0-2, …, lag 0-6).

Separate models were estimated for each exposure with the three outcomes, with averaged exposures including 24-hour PM_2.5_ (μg/m^3^), 1-hour maximum NO_2_ (ppb), 8-hour maximum O_3_ (ppb), 24-hour maximum ambient temperature (°C), and average 24-hour atmospheric pressure (Pa). Given that the case-crossover design inherently controls for individual-level confounders (such as fixed characteristics like age and sex), the number of potential confounders was limited to environmental exposures that could vary over time and potentially affect the risk of suicide or overdose. Specifically, the air pollution models (ie, PM_2.5_, NO_2_, O_3_) were adjusted for maximum temperature, average 24-hour precipitation (mm/day), and average 24-hour short-wave solar radiation (W/m^2^). Maximum temperature models were adjusted for PM_2.5_, precipitation, and solar radiation. Atmospheric pressure models were adjusted for PM_2.5_, temperature, precipitation, and solar radiation.

Air pollutants (PM_2·5_, NO_2_, O_3_), maximum temperature, and atmospheric pressure were modeled as continuous linear terms^(11)^ consistent with prior studies.^41–44^ Precipitation and solar radiation were modeled using natural cubic splines to account for potential nonlinear confounding.^45^

Effect modification was assessed by stratifying the models to estimate subgroup-specific effects across key geographic and temporal dimensions including season (spring, summer, fall, winter), U.S. Census region (Northeast, North Central, South, West), urbanicity (metro vs non-metro), and elevation (above vs below 500 m).

Results are expressed as adjusted odds ratios (ORs) and 95% confidence intervals (CIs) calculated per interquartile range (IQR) increase of each exposure. All statistical analyses were conducted in R (version 4.2.1), using the survival package (version 3.5-5) for conditional logistic regression (*clogit* function with *method=“breslow”*).

## Results

### Study population characteristics

Table 1 presents demographic and geographic characteristics of the study population. Between January 1, 2018, and December 31, 2019, we identified 6389 deaths due to suicide, 4326 due to drug overdose, and 2761 due to opioid overdose among U.S. Veterans. The average (±SD) age at death was 60 (19) years for suicide, 54 (14) years for drug overdose, and 52 (14) years for opioid overdose. Across all outcomes, more than 90% of decedents were male. Deaths were relatively evenly distributed across seasons, with slightly higher counts in summer for suicide. Most decedents resided in metropolitan areas (79-90%). The South had the largest proportion of deaths for all outcomes (37-43%), followed by the West, North Central, and Northeast regions (for additional detail by Census division, see Table S1). Most decedents lived at lower elevations (<500 m; 84-92%). Analyses stratified by region and elevation were limited to Veterans residing in the continental United States.

Table S2 displays the distribution of the environmental exposures on cumulative lags. Fine particulate matter levels were modestly higher on case days compared to control days for overdose and opioid overdose deaths. Mean atmospheric pressure was slightly lower on case vs control days among suicide deaths. Other environmental variables, including NO_2_, O_3_, precipitation, solar radiation, and maximum temperature, showed little difference between case and control days. Single lag exposure measures in Table S3 were generally consistent with cumulative lag exposures.

Figure S3 presents the distributions of environmental exposures overall and across subgroups defined by season, region, urbanicity, and elevation, illustrating baseline IQR differences on case and control days. The observed variation across strata—for example, higher baseline maximum temperatures during summer months—provides important context for seasonal comparisons and subgroup-specific associations with suicide and overdose mortality.

### Population-wide analysis

**Figure 1:**
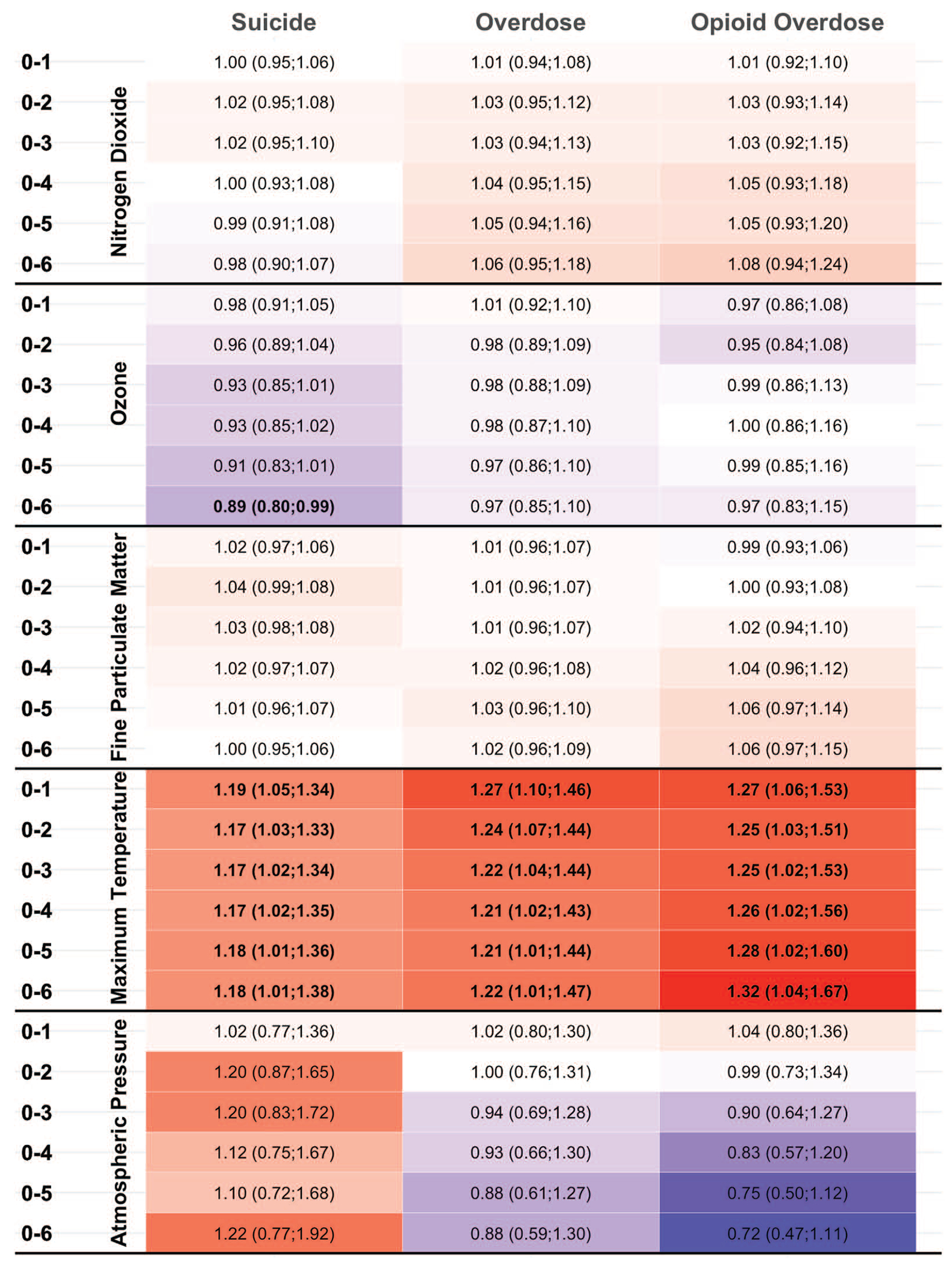
Adjusted ORs and 95% CIs estimated for each exposure across *cumulative lag days* (0-1 to 0-6) for suicide, drug overdose, and opioid overdose outcomes across the entire population. Estimates with 95% CIs that do not cross 1 are highlighted in bold, with blue shading indicating ORs < 1 and red shading indicating ORs > 1. Shading darkness increases with distance from 1. Air pollution models (PM_2.5_, NO_2_, O_3_) were adjusted for maximum temperature, precipitation (mm/day), and shortwave solar radiation (W/m^2^). Maximum temperature models were adjusted for PM_2.5_, precipitation, and solar radiation. Atmospheric pressure models were adjusted for PM_2.5_, maximum temperature, precipitation, and solar radiation. Results are expressed as adjusted ORs calculated per IQR increase of each exposure. Abbreviations: 95% CI, 95% confidence interval; IQR, interquartile range; OR, odds ratio; PM_2.5_, fine particulate matter; NO_2_, nitrogen dioxide; O_3_, ozone.

Figure 1 presents the adjusted ORs and 95% CIs for the associations between environmental exposures and mortality outcomes on cumulative lag days. An IQR increase in maximum temperature was associated with elevated odds of death across all three outcomes across the entire population. For example, an IQR increase in maximum temperature over cumulative lag days 0-1 was associated with higher odds of suicide (OR, 1.19; 95% CI, 1.05-1.34), drug overdose (OR, 1.27; 95% CI, 1.10-1.46), and opioid overdose (OR, 1.27; 95% CI, 1.06-1.53). Similar associations were observed for cumulative lag days 0-6: suicide (OR, 1.18; 95% CI, 1.01-1.38), overdose (OR, 1.22; 95% CI, 1.01-1.47), and opioid overdose (OR, 1.32; 95% CI, 1.04-1.67). Single lag models showed associations primarily on lag 0 and lag 1 days, with attenuated estimates on preceding lag days (Figure S4).

### Stratified analyses

**Figure 2:**
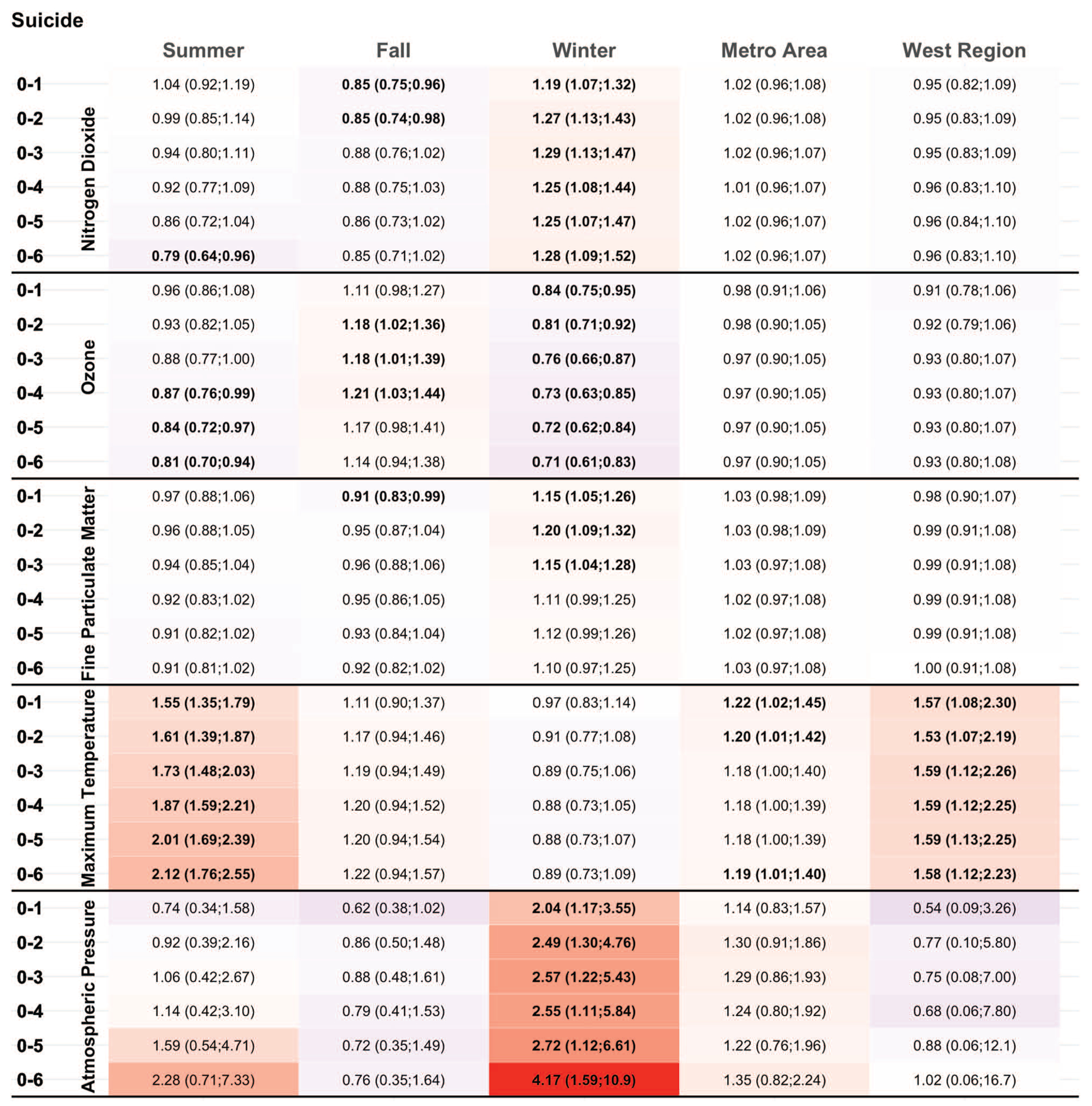
Adjusted ORs and 95% CIs for *suicide* deaths across *cumulative lag days* (0-1 to 0-6), presented for the most relevant pattern among the models stratified by season, region, rural/urban classification, and elevation. Estimates with 95% CIs that do not cross 1 are highlighted in bold, with blue shading indicating ORs < 1 and red shading indicating ORs > 1. Shading darkness increases with distance from 1. Air pollution models (PM_2.5_, NO_2_, O_3_) were adjusted for maximum temperature, precipitation (mm/day), and shortwave solar radiation (W/m^2^). Maximum temperature models were adjusted for PM_2.5_, precipitation, and solar radiation. Atmospheric pressure models were adjusted for PM_2.5_, maximum temperature, precipitation, and solar radiation. Results are expressed as adjusted ORs calculated per IQR increase of each exposure. Abbreviations: 95% CI, 95% confidence interval; IQR, interquartile range; OR, odds ratio; PM_2.5_, fine particulate matter; NO_2_, nitrogen dioxide; O_3_, ozone.

**Figure 3:**
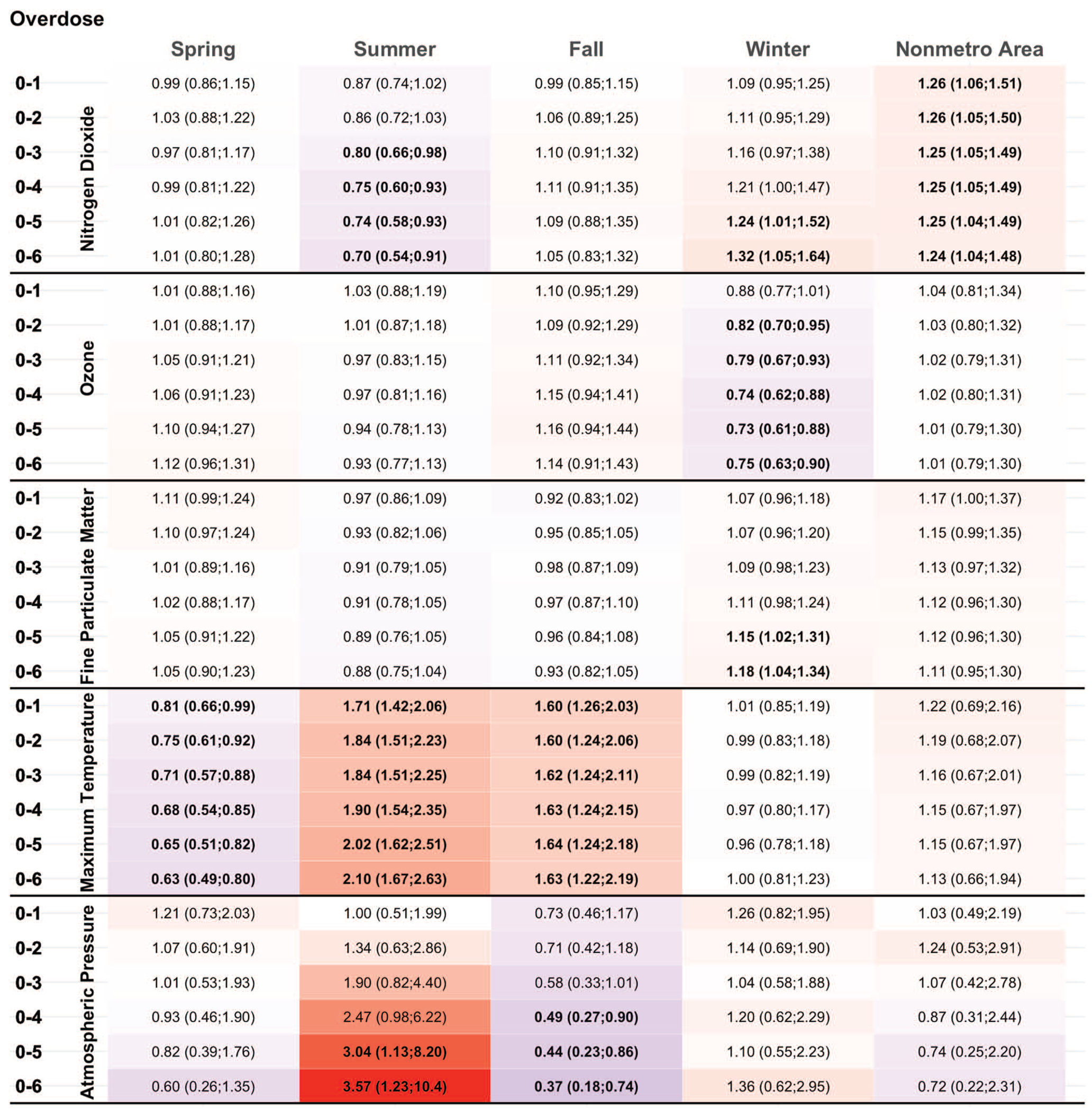
Adjusted ORs and 95% CIs for *overdose* deaths across *cumulative lag days* (0-1 to 0-6), presented for the most relevant pattern among the models stratified by season, region, rural/urban classification, and elevation. Estimates with 95% CIs that do not cross 1 are highlighted in bold, with blue shading indicating ORs < 1 and red shading indicating ORs > 1. Shading darkness increases with distance from 1. Air pollution models (PM_2.5_, NO_2_, O_3_) were adjusted for maximum temperature, precipitation (mm/day), and shortwave solar radiation (W/m^2^). Maximum temperature models were adjusted for PM_2.5_, precipitation, and solar radiation. Atmospheric pressure models were adjusted for PM_2.5_, maximum temperature, precipitation, and solar radiation. Results are expressed as adjusted ORs calculated per IQR increase of each exposure. Abbreviations: 95% CI, 95% confidence interval; IQR, interquartile range; OR, odds ratio; PM_2.5_, fine particulate matter; NO_2_, nitrogen dioxide; O_3_, ozone.

**Figure 4:**
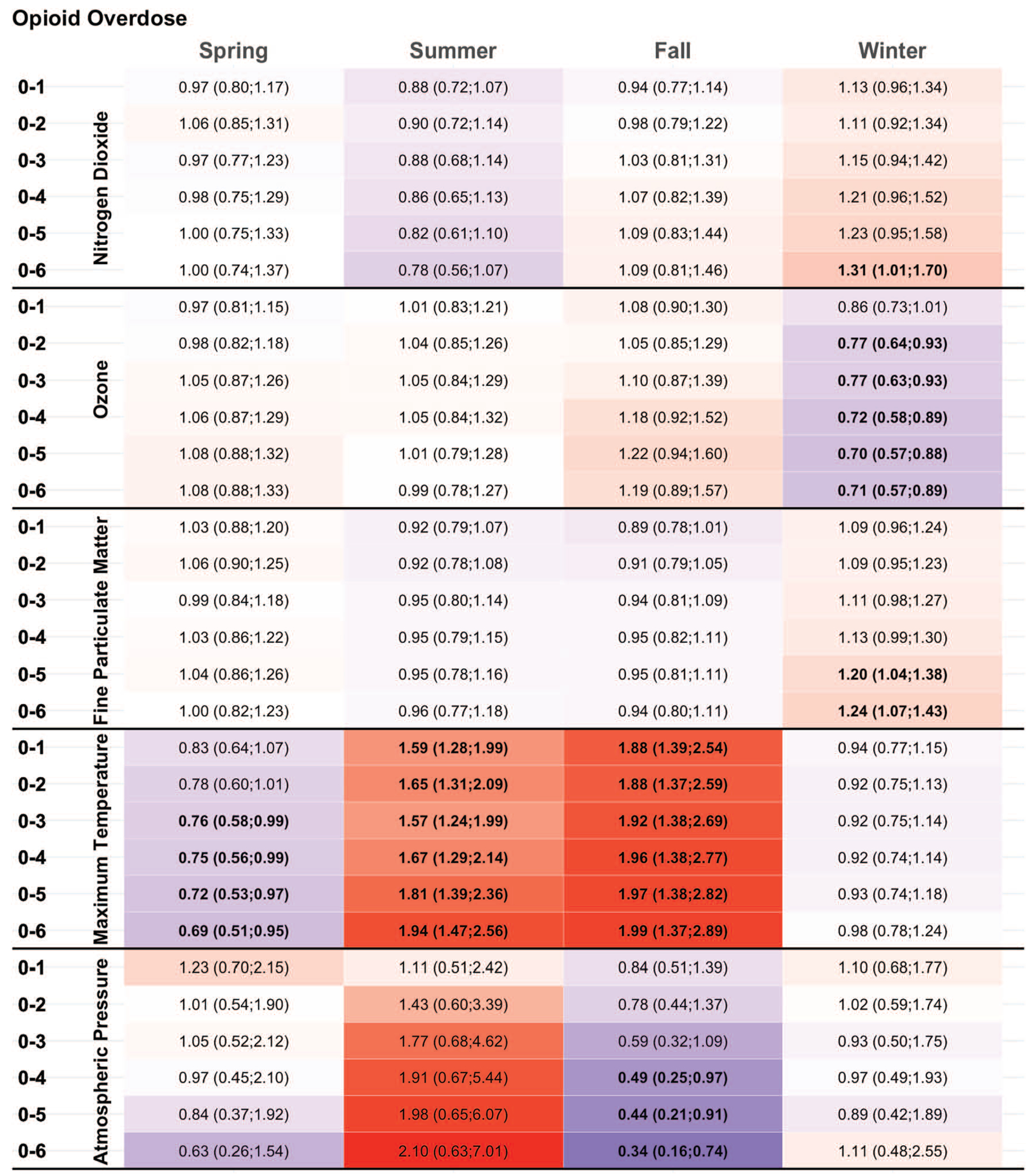
Adjusted ORs and 95% CIs for deaths due to *opioid overdose* across *cumulative lag days* (0-1 to 0-6), presented for the most relevant pattern among the models stratified by season, region, rural/urban classification, and elevation. Estimates with 95% CIs that do not cross 1 are highlighted in bold, with blue shading indicating ORs < 1 and red shading indicating ORs > 1. Shading darkness increases with distance from 1. Air pollution models (PM_2.5_, NO_2_, O_3_) were adjusted for maximum temperature, precipitation (mm/day), and shortwave solar radiation (W/m^2^). Maximum temperature models were adjusted for PM_2.5_, precipitation, and solar radiation. Atmospheric pressure models were adjusted for PM_2.5_, maximum temperature, precipitation, and solar radiation. Results are expressed as adjusted ORs calculated per IQR increase of each exposure. Abbreviations: 95% CI, 95% confidence interval; IQR, interquartile range; OR, odds ratio; PM_2.5_, fine particulate matter; NO_2_, nitrogen dioxide; O_3_, ozone.

Figures 2–4 present ORs across cumulative lag periods, stratified by season, geographic region, urbanicity, and elevation. Only strata in which at least one exposure showed a statistically significant association are shown. The full set of results for all exposures and strata are provided in Tables S4–S6 (cumulative lag days) and Tables S7–S9 (single-day lags); results for single-day lags were generally consistent with the patterns observed in the cumulative lag analyses.

During winter, an IQR increase in NO_2_ exposure was significantly associated with heightened suicide risk across all cumulative lags (eg, cumulative lag 0-6: OR, 1.28; 95% CI, 1.09-1.52), and with overdose on longer lags (eg, cumulative lag 0-6: OR, 1.32; 95% CI, 1.05-1.64) including opioid overdose (eg, cumulative lag 0-6: OR, 1.31; 95% CI 1.01-1.70). In contrast, NO_2_ was inversely associated with suicide (eg, cumulative lag 0-6: OR, 0.79; 95% CI, 0.64-0.96), and overdose (cumulative lag 0-6: OR, 0.70; 95% CI, 0.54-0.91) in the summer. An inverse association was measured in fall for an IQR increase in NO_2_, particularly for suicide on some cumulative lag days (eg, cumulative lag 0-1: OR, 0.85; 95% CI, 0.75-0.96). Additionally, an IQR increase in NO_2_ exposure was associated with overdose on all cumulative lags in nonmetropolitan counties (eg, cumulative lag 0-6: OR, 1.24; 95% CI, 1.04-1.48).

For PM_2.5_, positive associations were primarily measured in winter. An IQR increase was associated with suicide (eg, cumulative lag 0-1: OR, 1.15; 95% CI, 1.05-1.26), and with overdose (eg, cumulative lag 0-6: OR, 1.18; 95% CI, 1.04-1.34) and opioid overdose (eg, cumulative lag 0-6: OR, 1.24; 95% CI, 1.07-1.43).

Exposure to O_3_ was negatively associated with all outcomes during winter. In summer, ozone was inversely associated with suicide across cumulative lag days 0-4 to days 0-6. However, a positive association emerged in fall, with suicide risk increasing on lag 0-2 to 0-4.

Strong temperature effects were measured in summer. An IQR increase in maximum temperature was associated with significantly elevated odds across all three outcomes on all lag days: suicide (eg, cumulative lag 0-6: OR 2.12; 95% CI, 1.76-2.55), overdose (eg, cumulative lag 0-6: OR, 2.10; 95% CI, 1.67-2.63), and opioid overdose (eg, cumulative lag 0-6: OR, 1.94; 95% CI, 1.47-2.56). In fall, elevated maximum temperature was linked to increased odds on all lag days for overdose (eg, cumulative lag 0-6: OR, 1.63; 95% CI, 1.22-2.19) and opioid overdose (eg, cumulative lag 0-6: OR, 1.99; 95% CI, 1.37-2.89). Conversely, in spring ORs were significantly lower than 1.0 for all six cumulative lags for overdose (eg, cumulative lag 0-6: OR, 0.63; 95% CI, 0.49-0.80) and lags 0-3 to 0-6 for opioid overdose (eg, cumulative lag 0-6: OR, 0.69; 95% CI, 0.51-0.95). The association between an IQR increase in maximum temperature and suicide was found in the Western region on all cumulative lags (eg, cumulative lag 0-6: OR, 1.58; 95% CI, 1.12-2.23), but not in other regions. Similarly, an IQR increase in maximum temperature was associated with suicide on all cumulative lags in metropolitan counties (eg, lag 0-1: OR, 1.22; 95% CI, 1.02-1.45) but not in nonmetropolitan areas.

Mixed findings were measured for atmospheric pressure. In winter, an IQR increase in mean atmospheric pressure was associated with higher odds of suicide on all lag days (eg, cumulative lag 0-1: OR, 2.04; 95% CI, 1.17-3.55). In summer, an IQR increase in air pressure was associated with overdose on cumulative lag 0-5 to 0-6 (eg, cumulative lag 0-6: OR, 3.57; 95% CI, 1.23-10.40). In contrast, inverse associations were observed on lags 0-4 to 0-6 between pressure and both overdose (eg, cumulative lag 0-6: OR, 0.37; 95% CI, 0.18-0.74) and opioid overdose (eg, cumulative lag 0-6: OR, 0.34; 95% CI, 0.16-0.74).

## Discussion

### Air pollutants

In our study, we observed that elevated levels of PM_2.5_ and NO_2_ during winter were associated with an increased risk of suicide and drug overdose mortality. These winter-time effects of air pollutants align with previous research done in the general population on suicide^46^ and overall mortality.^43^ Elevated levels of PM_2.5_ and NO_2_ in winter—particularly in regions like the Intermountain West—are often driven by temperature inversions in mountainous topography and the increased use of combustible fuels for heating.^44^ Additionally, NO_2_ exposure was linked to increased overdose risk in nonmetropolitan counties, suggesting that Veterans residing in rural areas may be especially vulnerable to the negative health effects of air pollution potentially due to the higher prevalence of underlying health conditions in this group, more limited healthcare access, and greater exposure to localized emission sources.^47,48^ A negative association was observed between ozone and mortality outcomes in winter, which may reflect the fact that wintertime O_3_ typically represents lower, background concentrations that are inversely correlated with other pollutants. A potential biological mechanism underlying these associations involves pollutant-induced systemic inflammation and oxidative stress, which may disrupt neurochemical pathways, exacerbate mental health conditions, and heighten susceptibility to substance use and self-harm behaviors, which are more common conditions in Veterans than the general U.S. population. Additionally, the cumulative impact of environmental exposures overtime, including those encountered during military service, could further heighten Veterans’ sensitivity to short-term ambient air pollutants.

### Ambient temperature

In this study, increased maximum daily temperature was associated with higher risks of all three mortality outcomes: suicide, overdose, and opioid overdose, particularly in summer and fall. In contrast, increasing maximum daily temperature in the spring appeared to reduce the risk of overdose deaths. These findings align with prior research in the general population linking high temperatures to increasing suicide rates.^49,50^ However, in our cohort of U.S. Veterans (mean age ≈ 60 years), short-term increases in temperature during the summer were associated with more than a 2-fold increase in the risk of suicide and overdose death—substantially larger than the effect reported for heat in the general population in China (Realtive Risk = 1.44; mean age, 57.6 years).^51^ Several factors may contribute to heat’s amplified effect in Veterans. First, age plays a critical role in sensitivity to temperature extremes. With an average age of older than 50 years in our study, Veterans are more likely to experience age-related physiological changes, such as diminished thermoregulation and a reduced ability to cope with heat stress. These age-related changes, combined with pre-existing chronic health conditions, such as cardiovascular disease, respiratory issues, and substance use disorders, may elevate Veterans’ vulnerability to high temperature’s harmful effects potentially through mechanisms like neurodegeneration and behavioral dysregulation.^49,52–54^ The association between high temperatures and overdose mortality is also consistent with studies in the general population linking extreme heat to elevated risks of overdose mortality and morbidity.^9, 15, 55^ However, Veterans with specific characteristics including a history of substance use, such as cocaine and amphetamines, may face additional physiological challenges during heat events as these substances can impair the body’s ability to regulate temperature during periods of heat stress.^56^ Some substances of potential abuse can cause vasoconstriction and cardiovascular strain, compounding the effects of heat exposure and further increasing the risk of overdose, especially when combined with medications used to treat chronic conditions. Notably, the strongest associations were observed on immediate lag days (0-1), indicating a significant short-term effect. This suggests that Veterans may be particularly susceptible to rapid changes in temperature, which could be exacerbated by the acute physiological stress induced by both temperature extremes and pre-existing vulnerabilities. This may be compounded by service-related environmental exposures, such as those experienced in deployment settings, which could contribute to chronic inflammatory states and a lowered threshold for environmental stressors like extreme heat.

### Atmospheric pressure

Prior studies^41^ in general population samples have speculated that decreasing atmospheric pressure is associated with an increased risk of preventable deaths, such as suicide, potentially due to inducing hypoxia. However, in our study, we observed that increasing atmospheric pressure was associated with higher suicide risk in winter and greater overdose mortality in summer. It is possible that both rising and falling atmospheric pressure can influence brain function, including serotonin metabolism, thereby contributing to worsening mental health and suicidal behavior.^27,57^ Moreover, elevated pressure often coincides with specific environmental conditions—such as higher temperatures, reduced precipitation, and increased air pollution—that have themselves been linked to elevated suicide risk.^53^ Among U.S. Veterans, these associations may be amplified by service-related trauma, chronic mental health conditions, and physical injuries. Combat exposure may heighten sympathetic nervous system reactivity, increasing sensitivity to atmospheric shifts, while rising atmospheric pressure may intensify physiological stress responses and interact with conditions such as PTSD, depression, and anxiety to elevate risk of suicidal ideation or overdose. Weather-related exacerbations of musculoskeletal or neurological pain may further compound psychological distress and substance use vulnerability. Together, these factors reflect a cumulative burden that may heighten susceptibility to stress-related neurobiological disruptions linked to atmospheric changes potentially explaining the strong effects observed in Veterans compared with the general population, particularly in regions with marked seasonal variability.

### Potential mechanisms and public health implications

Short-term environmental exposures—such as elevated temperature, air pollution, and atmospheric pressure—may elevate suicide and overdose risk through interrelated *biological*, *behavioral*, and *psychosocial* mechanisms. These mechanisms are likely multifactorial, involving complex interactions between physiological stress responses and context-dependent behavioral patterns that may exacerbate existing vulnerabilities.

From a biological pathway, these exposures can trigger systemic inflammation, oxidative stress, and HPA axis activation^58^—processes implicated in affective dysregulation and psychiatric vulnerabil- ity.^13,14,59^ Analogous to early-life stressors that impair neurodevelopment, these environmental stimuli may act as acute destabilizing triggers, exacerbating symptoms of depression, anxiety, or substance use disorders.^60–62^ Stress-induced mitochondrial dysfunction and lipid membrane damage, mediated by cortisol, may further impair neurotransmitter signaling and stress resilience.^28^

Behavioral mechanisms are also likely contributors to the observed associations. In particular, Routine Activity Theory^63^ may provide a framework for understanding how environmental stressors, such as extreme heat or poor air quality, could influence behaviors that increase the risk of suicide or overdose. For instance, elevated temperatures may increase social interaction, time spent outdoors, and opportunities for substance use or impulsive behavior—particularly among individuals already at risk. Conversely, colder temperatures and high-pressure winter conditions may exacerbate social isolation, psychological distress, or access to care. These seasonal patterns may manifest differently across the year but point to a common pathway: that environmental extremes—regardless of season or direction—act as physiological stressors that compound the effects of other risk factors for suicide and overdose. Both outcomes are fundamentally disorders of impulsive relief seeking, and intolerable weather conditions may serve as one more stressor from which individuals seek relief. While these are plausible hypotheses, they remain untested in the current study. Our analysis focuses primarily on identifying associations, and further research would be necessary to investigate the behavioral mechanisms involved.

While unmeasured individual-level psychosocial factors, such as psychiatric comorbidities or substance use, are recognized limitations, the case-crossover design inherently controls for stable individual characteristics, minimizing confounding. Moreover, such factors are unlikely to vary systematically with short-term environmental exposures. However, they may interact with environmental stressors in complex ways. Future research incorporating granular behavioral and contextual data is needed to elucidate these interactions and better identify at-risk subgroups.

### Strengths and limitations

Study strengths include the use of national-scale data and the novel multi-exposure assessment of temperature, atmospheric pressure, and air pollution in relation to suicide and overdose mortality among U.S. Veterans. The case-crossover design enabled evaluation of short-term exposures, offering insight into potential immediate environmental triggers.

Limitations include potential exposure misclassification, as ambient temperature and pollution may not reflect individual exposures due to microclimates, indoor air quality, and behavior. Additionally, although maximum ambient temperature is a relatively crude measure of temperature’s effect on the human body, it is a commonly used and standardized metric in environmental health research, allowing for comparison across studies. While alternative measures, such as heat waves or heat index, could offer additional insights into heat exposure, we chose daily maximum temperature due to its widespread availability and consistency across spatial and temporal scales. In contrast, air pressure is a more consistent measure, given minimal differences between indoor and outdoor settings. Importantly, while the case-crossover design controls for time-invariant characteristics, residual confounding by time-varying factors remains possible. Short-term behavioral changes, such as sleep disruption,^64^ reduced physical activity,^65^ or changes in alcohol or substance use—potentially influenced by heat or weather conditions—may affect suicide risk but were not directly measured in the current study. Limited availability of data on these transient behaviors precluded adjustment for such factors. The absence of sensitivity analyses for such factors is a limitation and should be addressed in future studies. Moreover, environmental exposure data were aggregated to the county level using population-weighted means to harmonize differing spatial resolutions across datasets. This approach may mask within-county variability, particularly in geographically large or heterogeneous counties, and could lead to exposure misclassification. Lastly, findings may not generalize beyond the VHA-enrolled Veteran population, particularly for those without healthcare access or with different demographic characteristics. Future research should address the complex, multi-exposure nature of the different ambient exposures explored in the current study.

## Conclusions

Suicide and drug overdose remain among the most urgent public health crises in the United States, disproportionately affecting Veterans due to unique occupational exposures, service-related stressors, and mental health vulnerabilities. While prior research has linked short-term ambient exposures to suicide risk in civilian populations, our case-crossover study demonstrates that the magnitude of these associations—particularly for temperature-related suicide and overdose risk—is substantially greater among Veterans, indicating population-specific vulnerability. Moreover, the seasonality and geography of risk differ for Veterans, suggesting that climatic and infrastructural factors may uniquely interact with underlying health and behavioral patterns in this population. Importantly, environmental risk is not confined to urban settings; rural Veterans appear disproportionately affected, revealing a previously underrecognized environmental health disparity. Together, these findings underscore the need to integrate environmental monitoring and early warning systems into Veteran-focused prevention efforts, as environmental stressors may act as acute triggers within a population already burdened by chronic physiological and psychological stress.

## Supplementary Material

Supplemental materials

Supplementary material is available at the *American Journal of Epidemiology* online.

## Figures and Tables

**Table 1:** Summary statistics for Veterans receiving care within the Veterans Health Administration who died by suicide, drug overdose, or opioid overdose between 2018 and 2019.

	Suicide		Overdose		Opioid overdose	
	*n*	% or Mean (SD) [min; max]	n	% or Mean (SD) [min; max]	*n*	% or Mean (SD) [min; max]
**Sex**	6389		4326		2761	
Female	235	4%	257	6%	154	6%
Male	6154	96%	4069	94%	2607	94%
**Age**	6389	60 (19) [19;100]	4326	54 (14) [22;100]	2761	52 (14) [22;100]
**Season**	6389		4326		2761	
Spring	1649	26%	1144	26%	702	25%
Summer	1705	27%	1067	25%	695	25%
Fall	1507	24%	1051	24%	663	24%
Winter	1528	24%	1064	25%	701	25%
**Urbanicity**	6389		4326		2761	
Metro area	5068	79%	3817	88%	2480	90%
Nonmetro area	1321	21%	509	12%	281	10%
**Region**	6366		4321		2760	
North Central	1299	20%	931	22%	640	23%
Northeast	655	10%	850	20%	655	24%
South	2734	43%	1629	38%	1034	37%
West	1678	26%	911	21%	431	16%
**Elevation**	6366		4321		2760	
< 500 m	5374	84%	3905	90%	2532	92%
≥ 500 m	992	16%	416	10%	228	8%

For regional and elevation stratified analysis, we are considering only Veterans whose residence is within the continental U.S. Table S1 provides summary statistics of suicide and overdose mortality by U.S. Census division.

## Data Availability

The data that support the findings of this study are not publicly available due to the inclusion of sensitive person-level information that cannot be shared under existing legislative and institutional data protection requirements.

## Footnotes

1 The VHA is part of the U.S. Department of Veterans Affairs and provides healthcare to eligible Veterans. It is the largest healthcare system in the United States, offering services like hospital care, outpatient treatment, and mental health support, with a focus on conditions, such as PTSD and substance use disorders. The VHA operates numerous medical centers and clinics nationwide.

2 County-level residential data were the finest spatial resolution validated by VA geospatial experts at the time of data extraction. County-level assignment may also help reduce exposure misclassification due to daily mobility. Counties are also key partners in public health policy implementation, further supporting the relevance of this spatial scale.

3
https://daymet.ornl.gov/overview

4
https://cds.climate.copernicus.eu/datasets/reanalysis-era5-single-levels?tab=overview

5
https://www.epa.gov/cmaq

6
https://www.epa.gov/hesc/rsig-related-downloadable-data-files

7
https://hub.worldpop.org/project/categories?id=3

8
https://www.usgs.gov/coastal-changes-and-impacts/gmted2010

9
https://www.ers.usda.gov/data-products/rural-urban-commuting-area-codes/documentation

10
https://www2.census.gov/geo/pdfs/maps-data/maps/reference/us_regdiv.pdf

11 In a sensitivity analysis, to assess our assumptions of linearity, ANOVA models were formulated in which the primary linear model for each exposure was compared with a natural spline (three degrees of freedom) representation, while keeping all other parameters unchanged (Figure S1). For suicide outcomes and maximum temperature, there was limited evidence of improved fit at certain cumulative lags; however, for overdose outcomes, all tests were non-significant. Inspection of the spline coefficients showed that ns2, the key indicator of non-linearity, was non-significant across all cumulative lags, outcomes, and exposures (Figure S2), indicating that non-linear components did not meaningfully contribute to the exposure-response relationship. Given the lack of evidence for nonlinearity, the added complexity of spline models, and the interpretability of linear terms, we retained the linear exposure specification for the primary analyses.
