# Supplementary material for: Association of short-term ambient environmental exposures with suicide and drug overdose deaths among U.S. Veterans
Source: Am J Epidemiol. Author manuscript; Available in PMC 2026 Jul 27. (PMC13405249; doi:10.1093/aje/kwag099)
Supplement: Supplemental materials [file NIHMS2190379-supplement-Supplemental_materials.docx]

**Association of short-term ambient environmental exposures with suicide and drug overdose deaths among U.S. Veterans**

Alina Peluso, Ph.D^1,*^; Dirga Lamichhane, Ph.D^2^; James A. VanDerslice, Ph.D^2^; Jeremy Logan, Ph.D^1^;

Anuj Kapadia, Ph.D^1^; Jodie A. Trafton, Ph.D^3^; Amanda V. Bakian, Ph.D^2^

^1^ Oak Ridge National Laboratory, Oak Ridge, TN, USA

^2^ University of Utah, Salt Lake City, UT, USA

^3^ U.S. Department of Veterans Affairs, Veterans Health Administration, Palo Alto, CA, USA

*Corresponding Author: Alina Peluso, PhD, Research Scientist in Biostatistics, Advanced Computing for Health Sciences Section, Computational Sciences and Engineering Division, Oak Ridge National Laboratory. Email [pelusoa@ornl.gov](mailto:pelusoa@ornl.gov)

**Supplementary Figures and Tables**

**Figure S1**

**Figure S2**

**Figure S3A–E**

**Figure S4**

**Table S1**

**Table S2**

**Table S3**

**Table S4**

**Table S5**

**Table S6**

**Table S7**

**Table S8**

**Table S9**

**Figure S1:** ANOVA p-values comparing nonlinear versus linear exposure–response specifications for suicide risk (A), overdose (B) and opioid overdose (C) by air pollution exposure and lag structure. Each panel shows a different exposure type (atmospheric pressure, maximum temperature, PM_2.5_, NO_2_, and Ozone). Bars indicate p-values for single-day and cumulative lag models, and dashed lines represent common significance thresholds (α = 0.01, 0.05). P-values below a threshold suggest that allowing for nonlinearity improves model fit; p-values above the threshold indicate no evidence that nonlinear specification provides a better fit, supporting the linear specification.

| **(A)** | **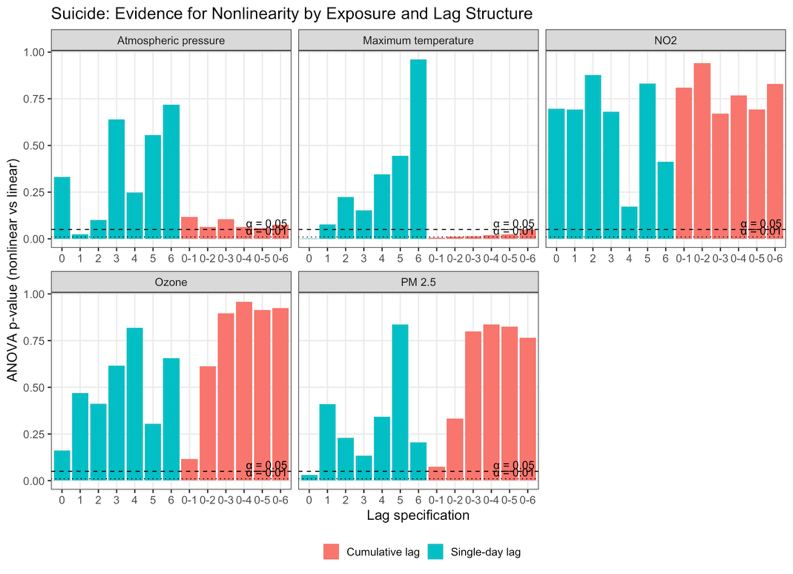** |
| --- | --- |
| **(B)** | **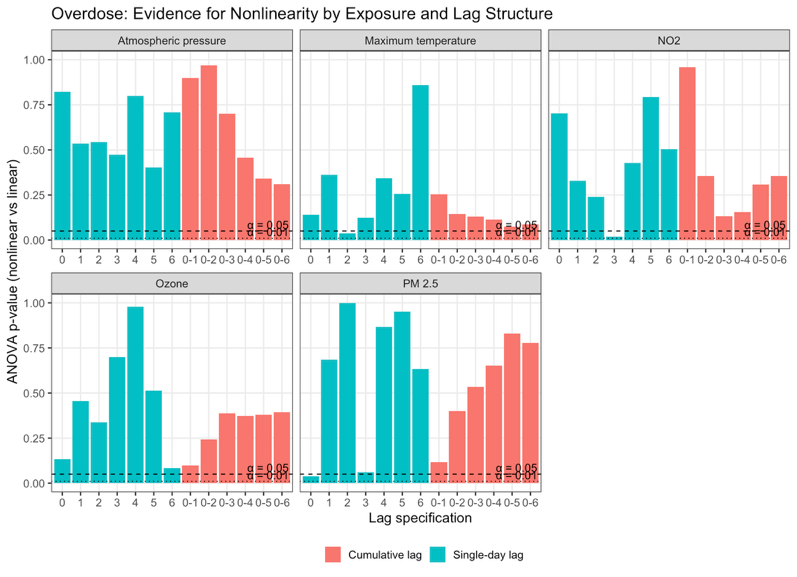** |
| **(C)** | **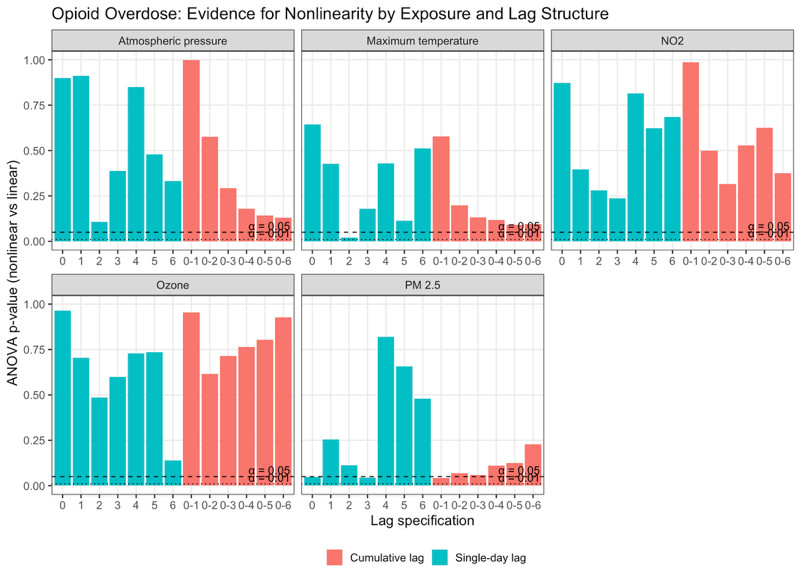** |

**Figure S2:** Exponentiated coefficients (with 95% confidence intervals) from a natural spline model with 3 degrees of freedom (ns1–ns3) by cumulative lag (0-1 to 0-6 days), outcome, and exposure. Each spline basis represents a component of the exposure-response curve: ns1 approximates the linear trend, while ns2 and ns3 capture potential non-linear deviations. The key indicator of non-linearity, ns2, was non-significant across all exposures, indicating no evidence of meaningful non-linear effects. This supports the use of a simpler linear model, which captures the main exposure-outcome associations.
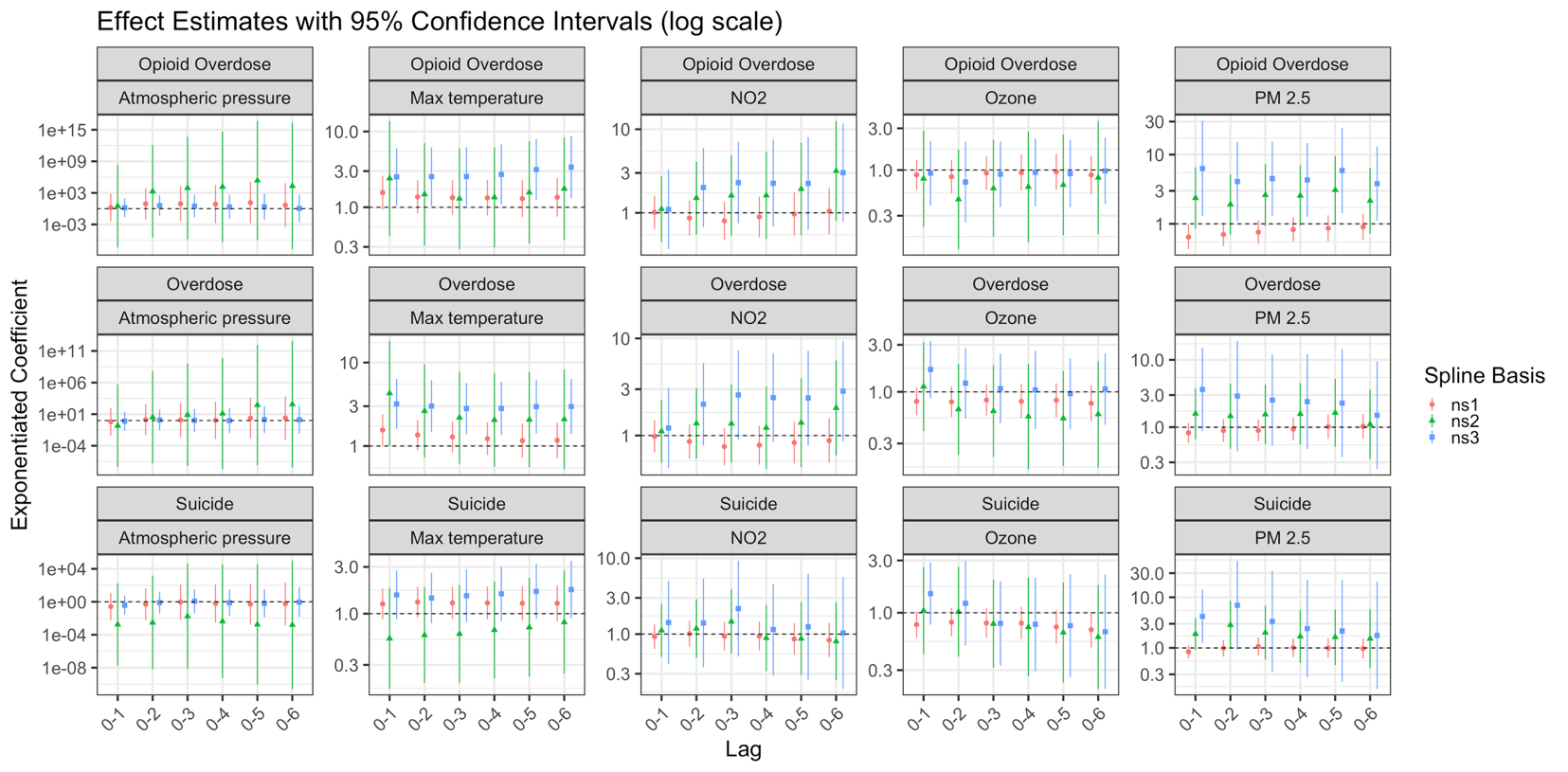


**Figure S1A–E.** Distributions of environmental exposures, including daily atmospheric pressure, maximum temperature, PM_2.5_, NO_2_, and O_3_, shown overall (A) and stratified by season (B), region (C), urbanicity (D), and elevation (E). These plots illustrate baseline IQR differences in exposure levels on case (red) and control (blue) days overall and across subgroups—for example, higher maximum temperatures during summer months—which provide important context for interpreting seasonal contrasts and subgroup-specific associations with suicide and overdose mortality.

1. Overall:

| *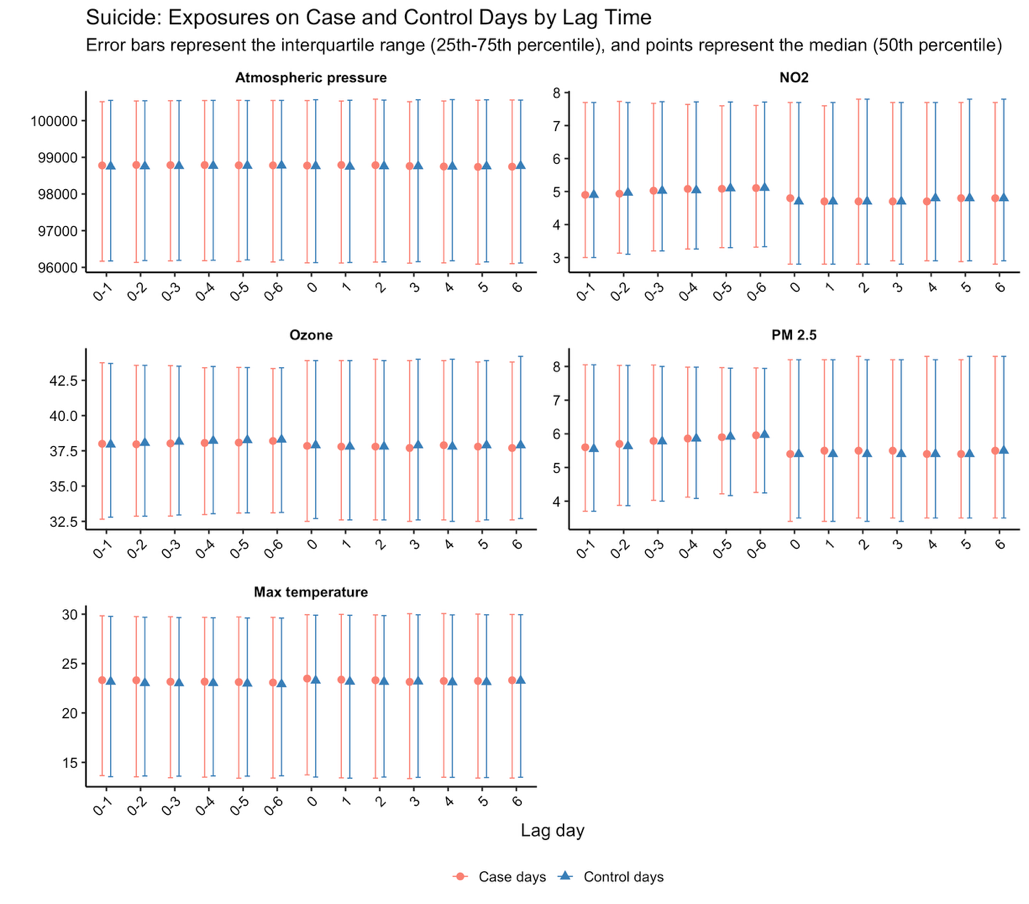* |
| --- |
| *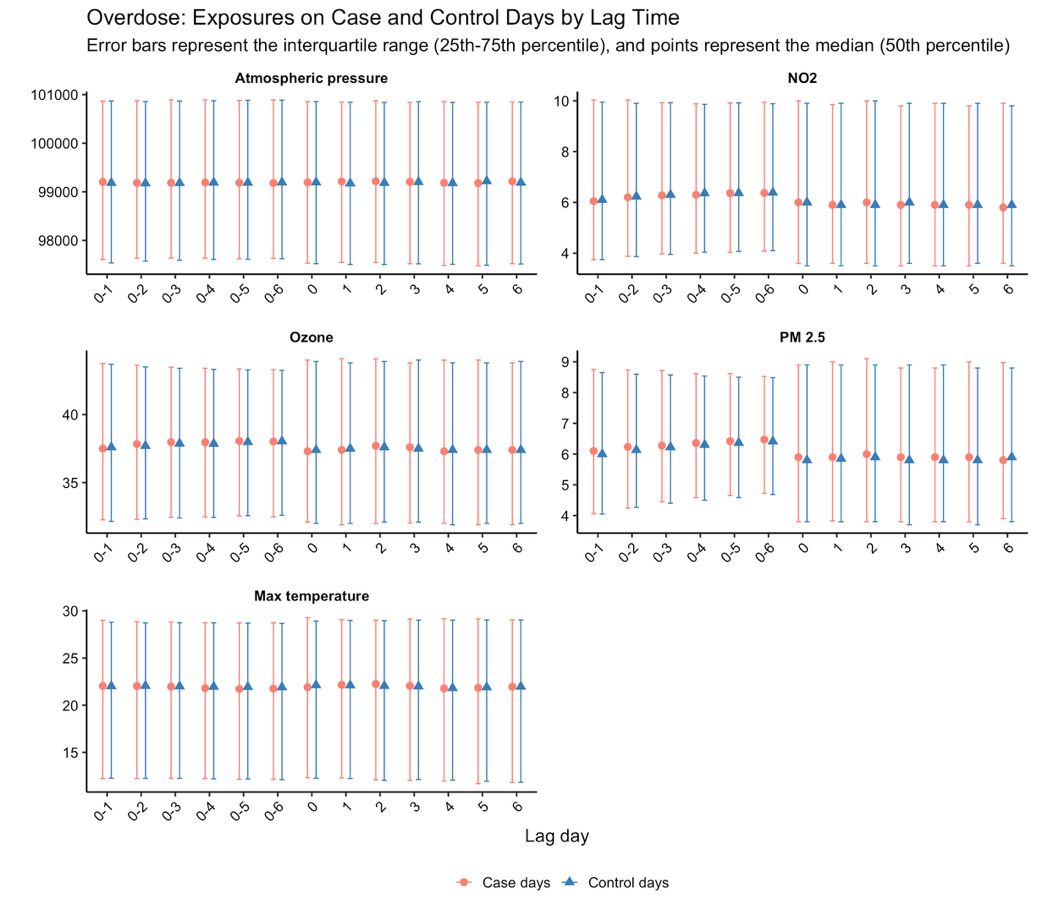* |
| *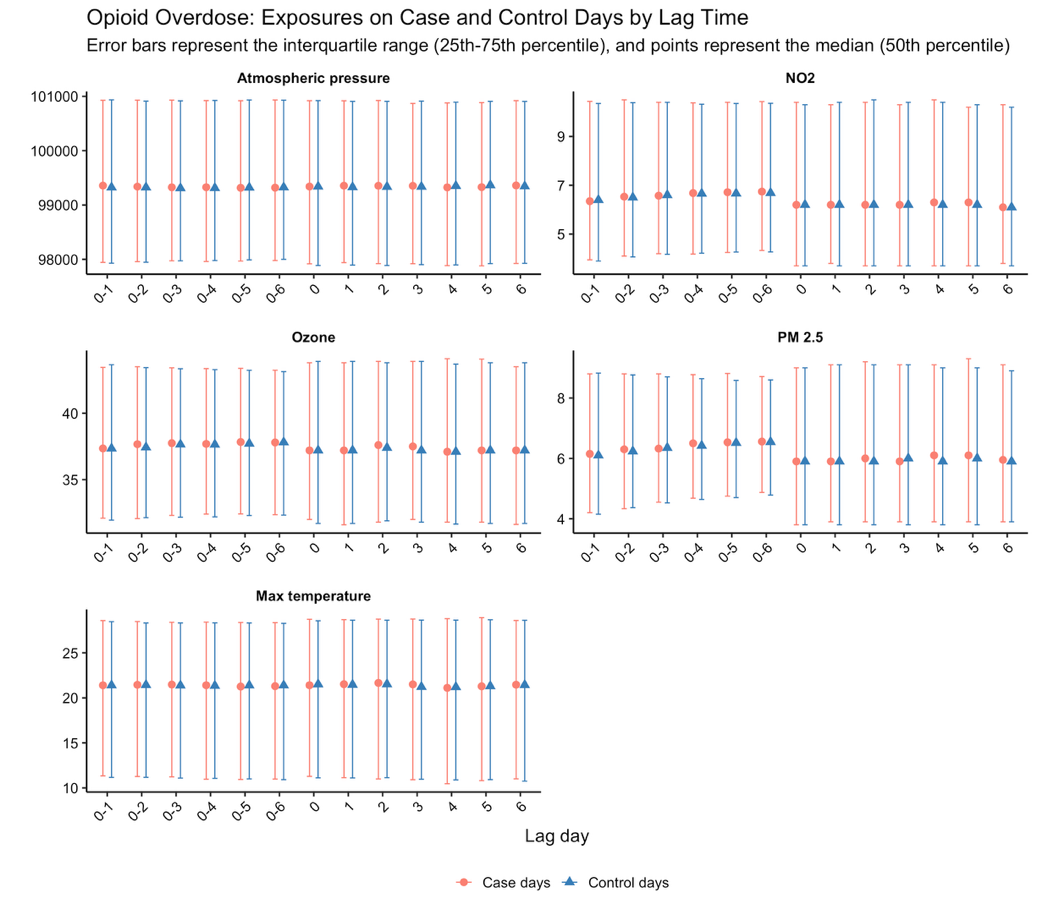* |

1. By season:

| *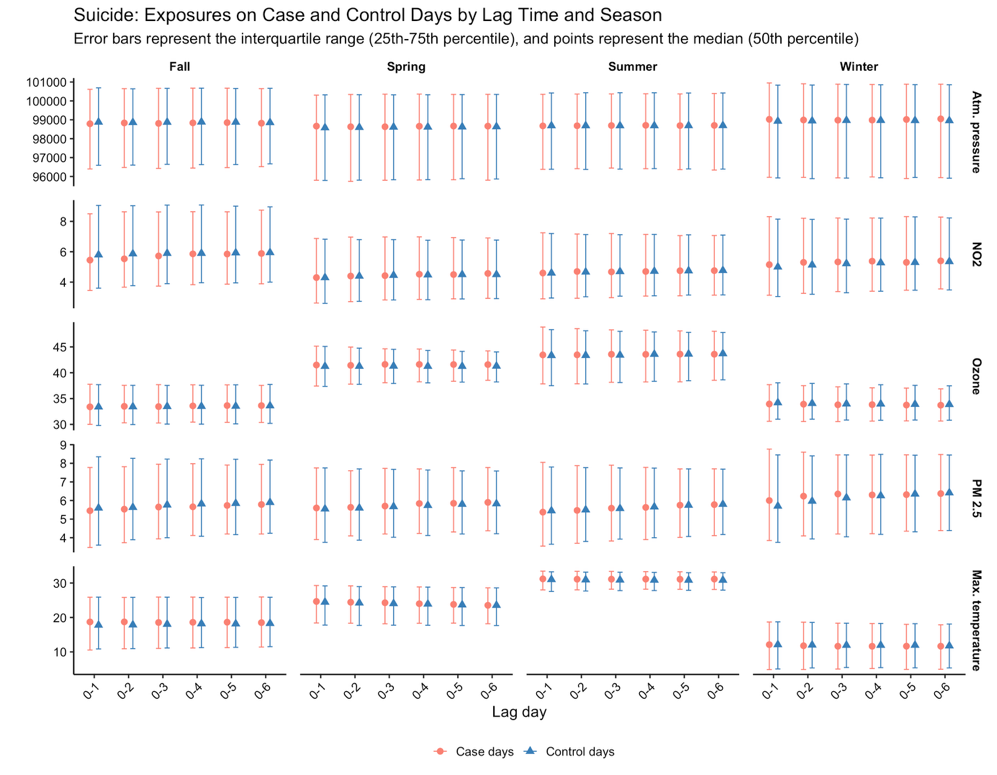* |
| --- |
| *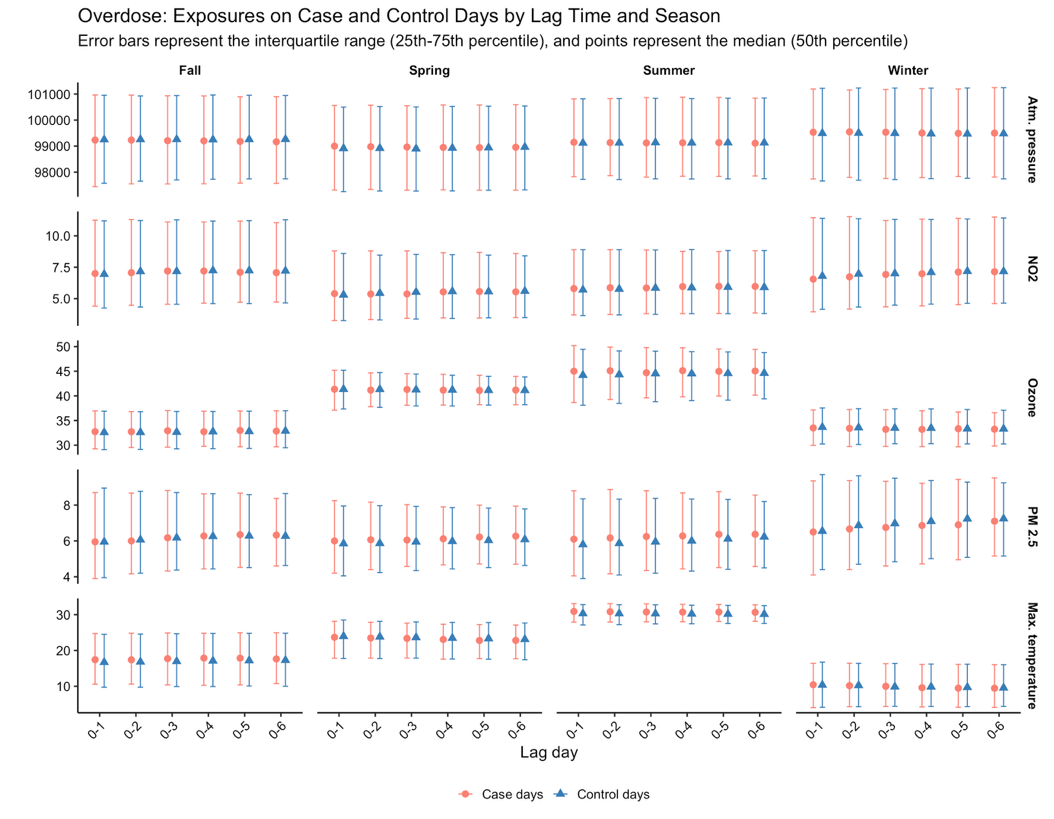* |
| *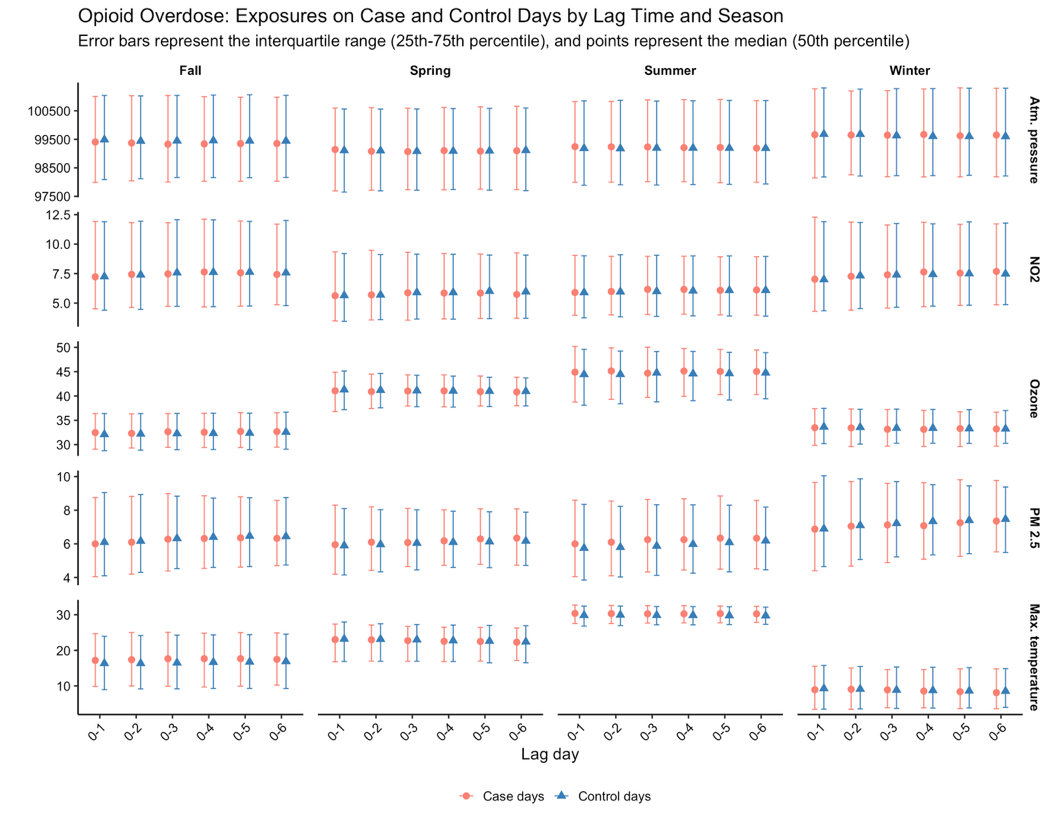* |

1. By U.S. region:

| *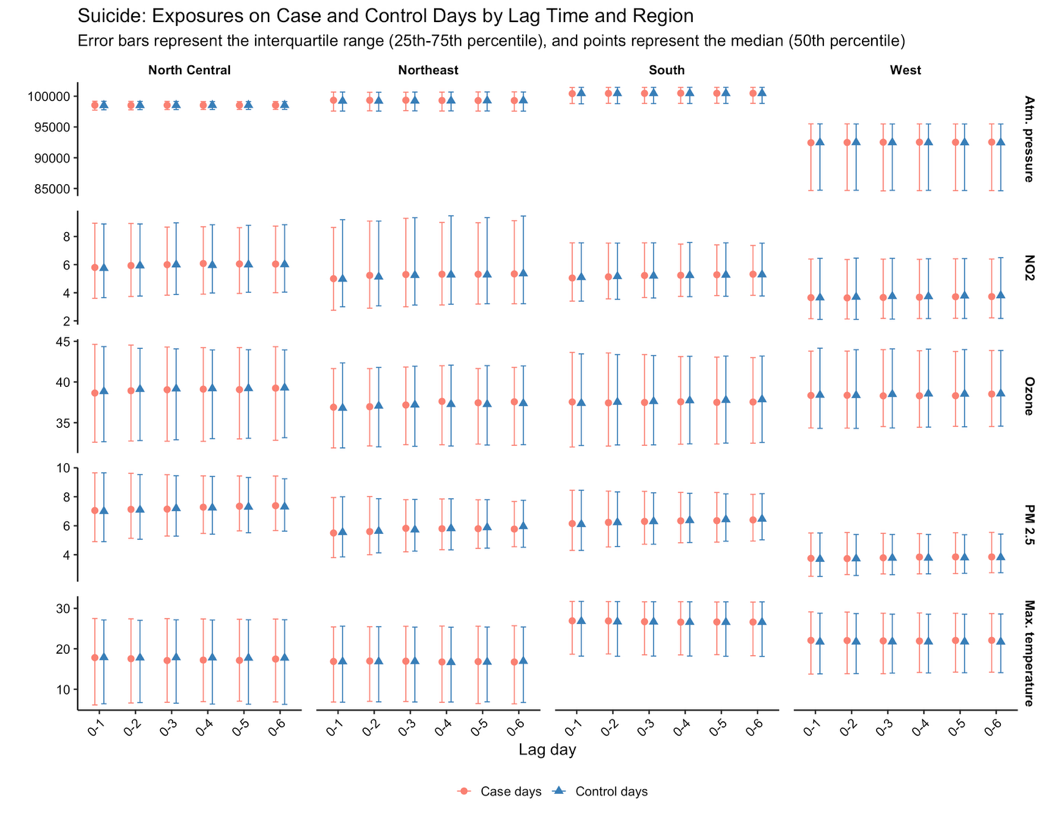* |
| --- |
| *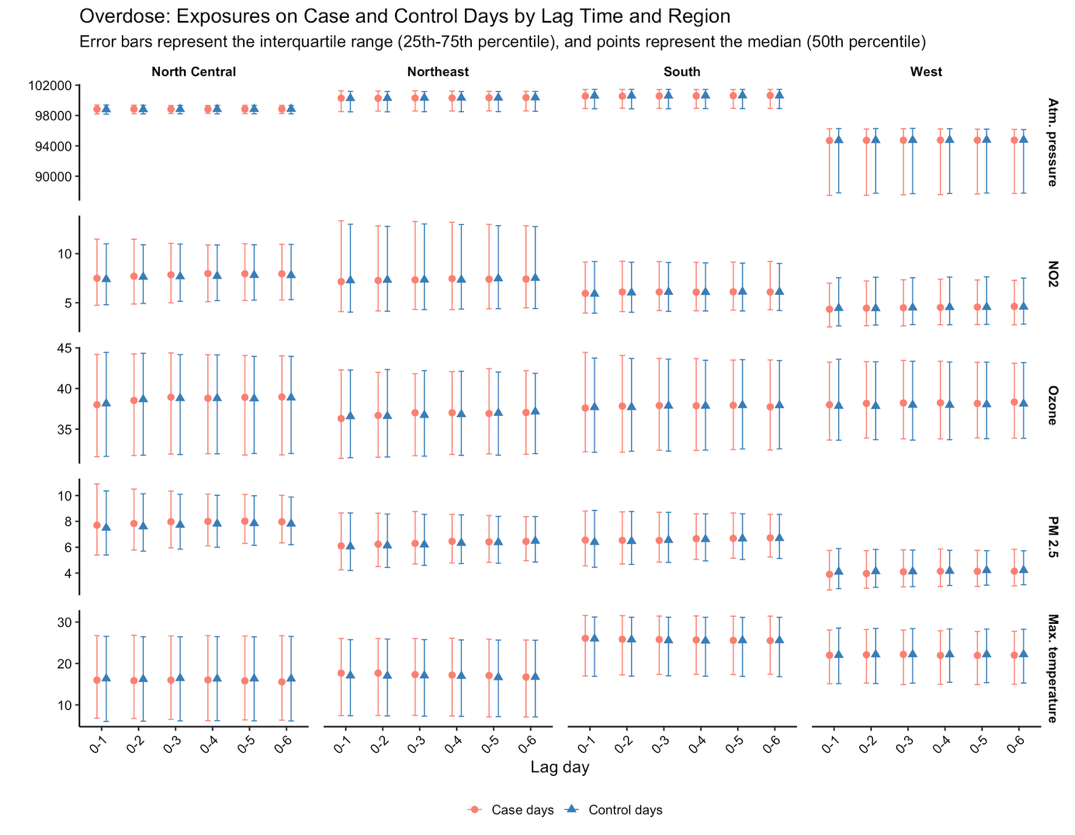* |
| *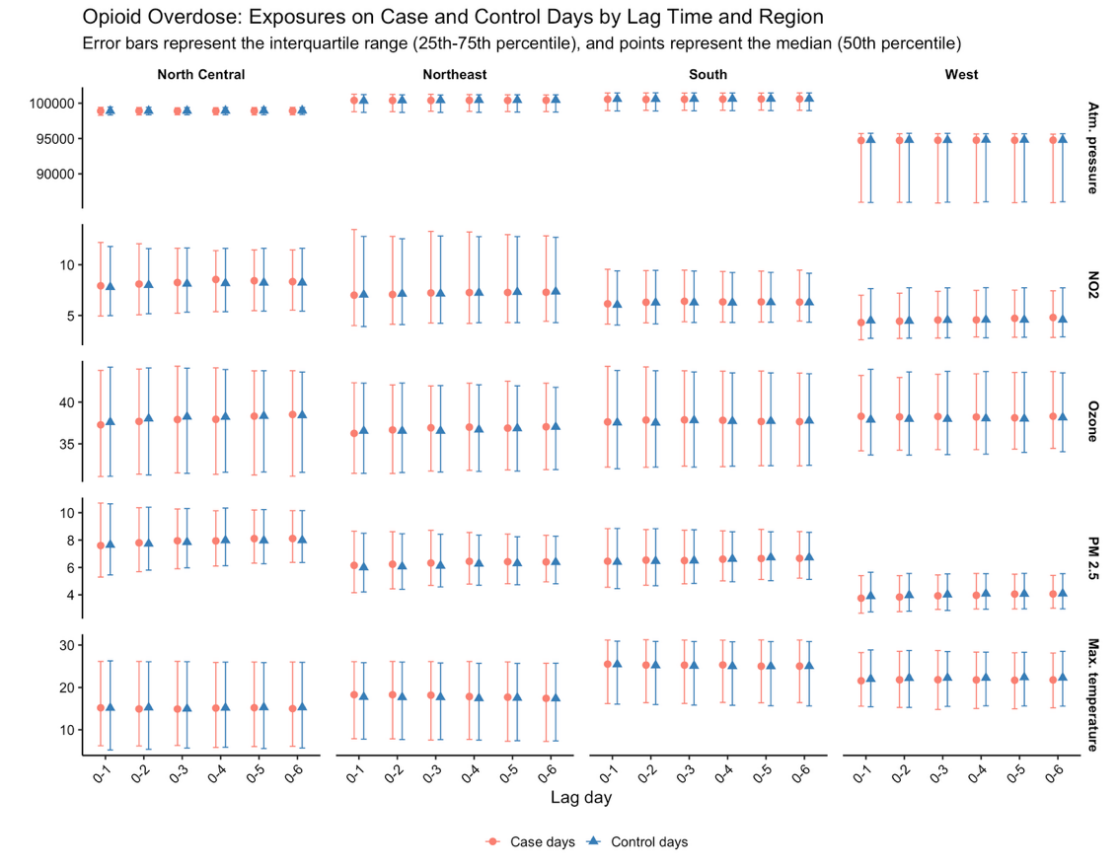* |

1. By urbanicity:

| *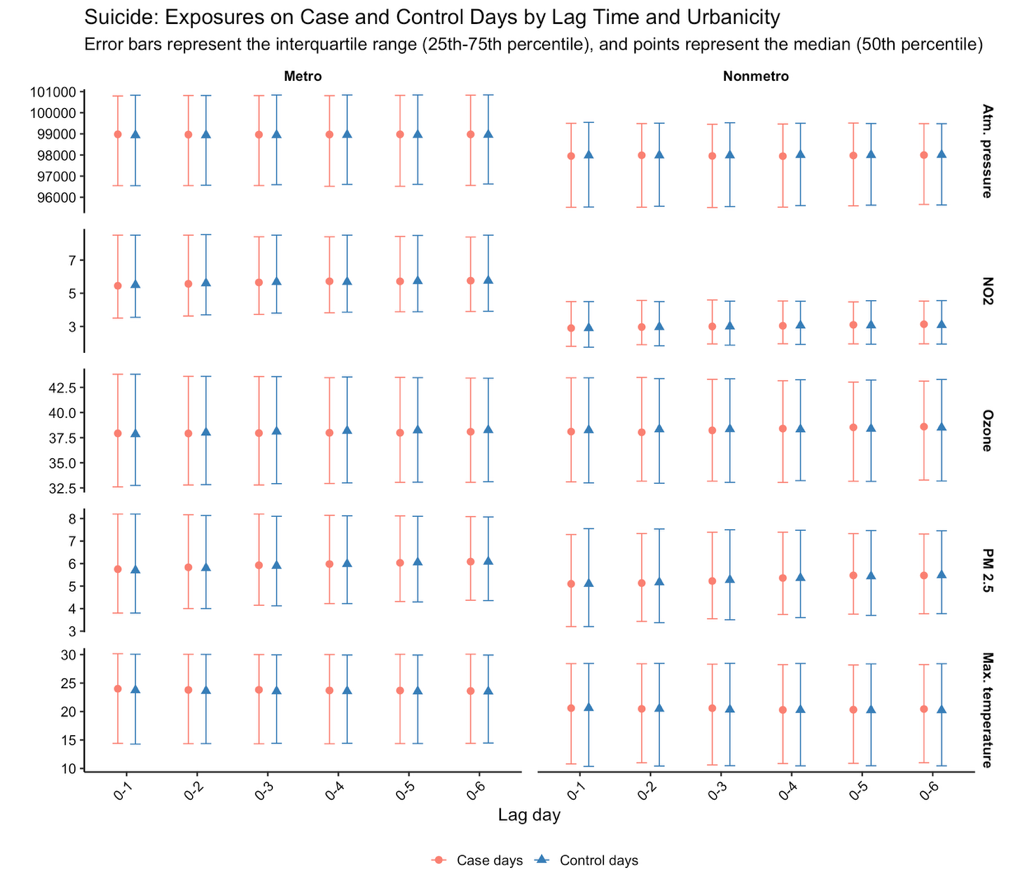* |
| --- |
| *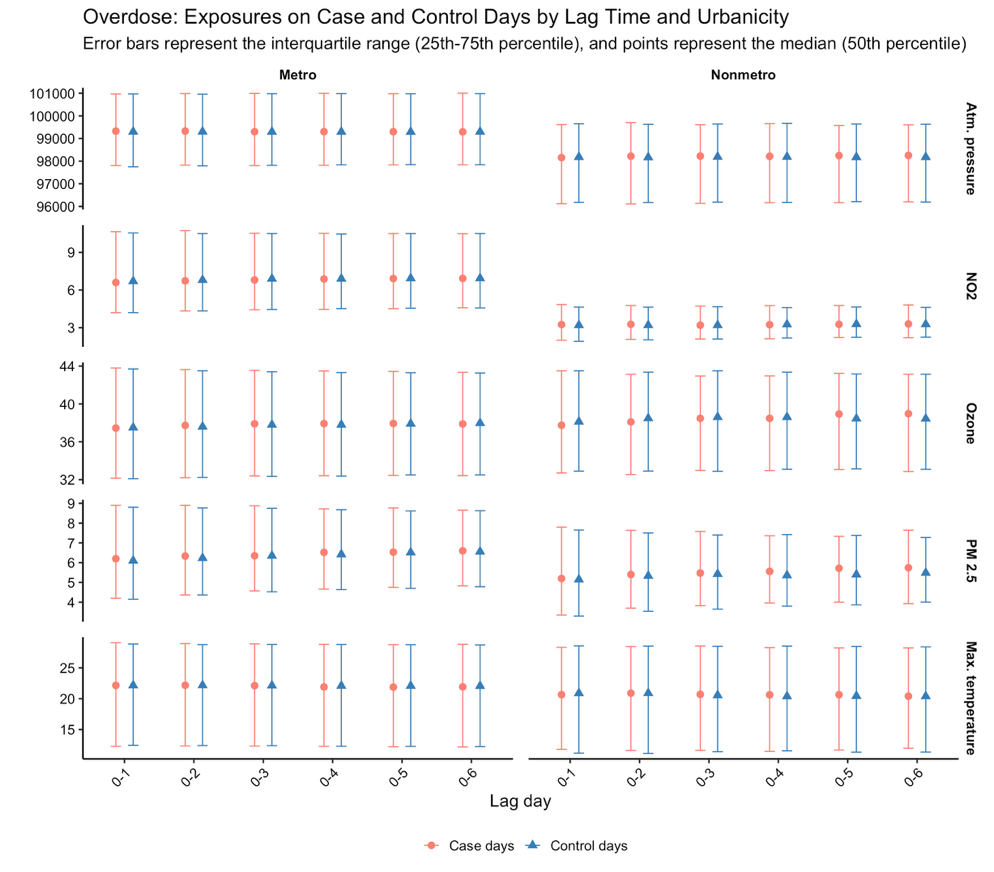* |
| *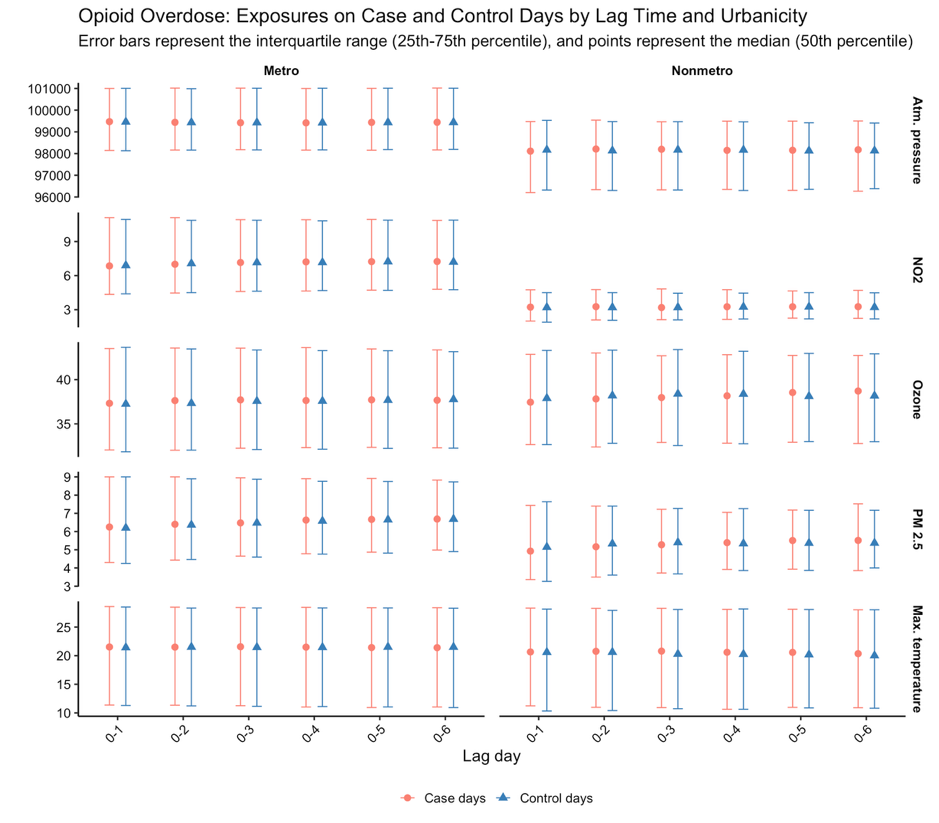* |

1. By elevation:

| *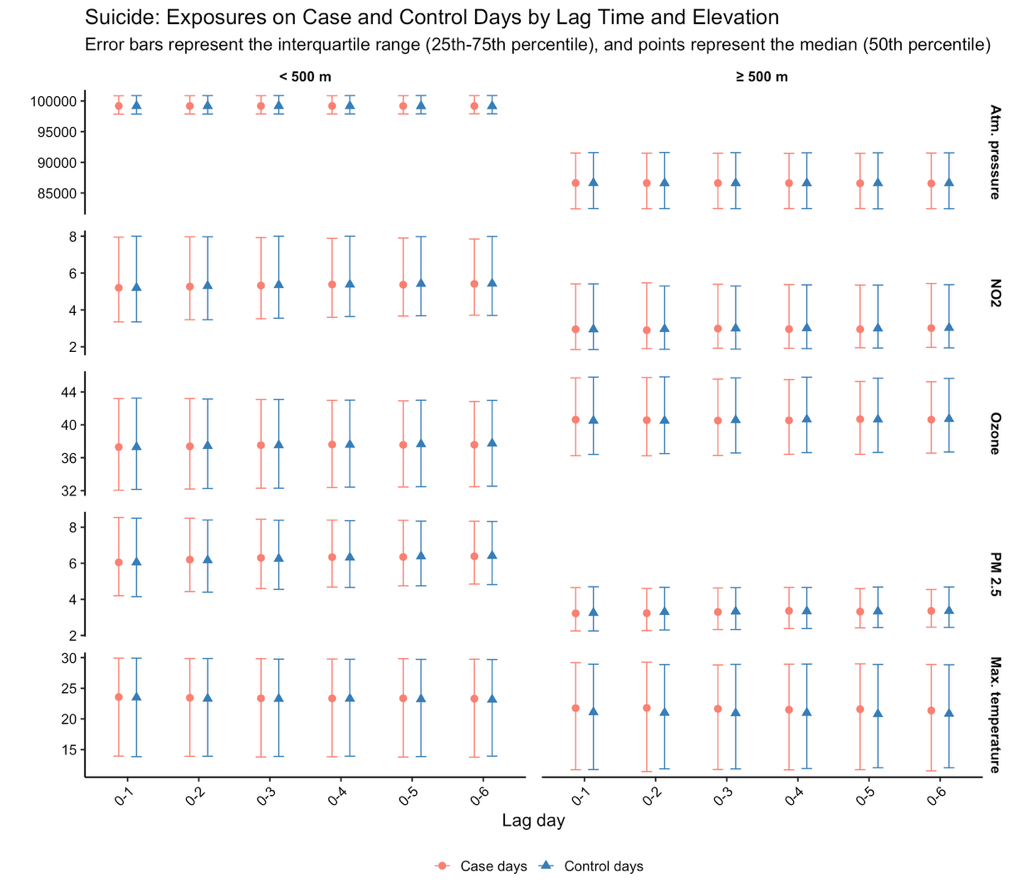* |
| --- |
| *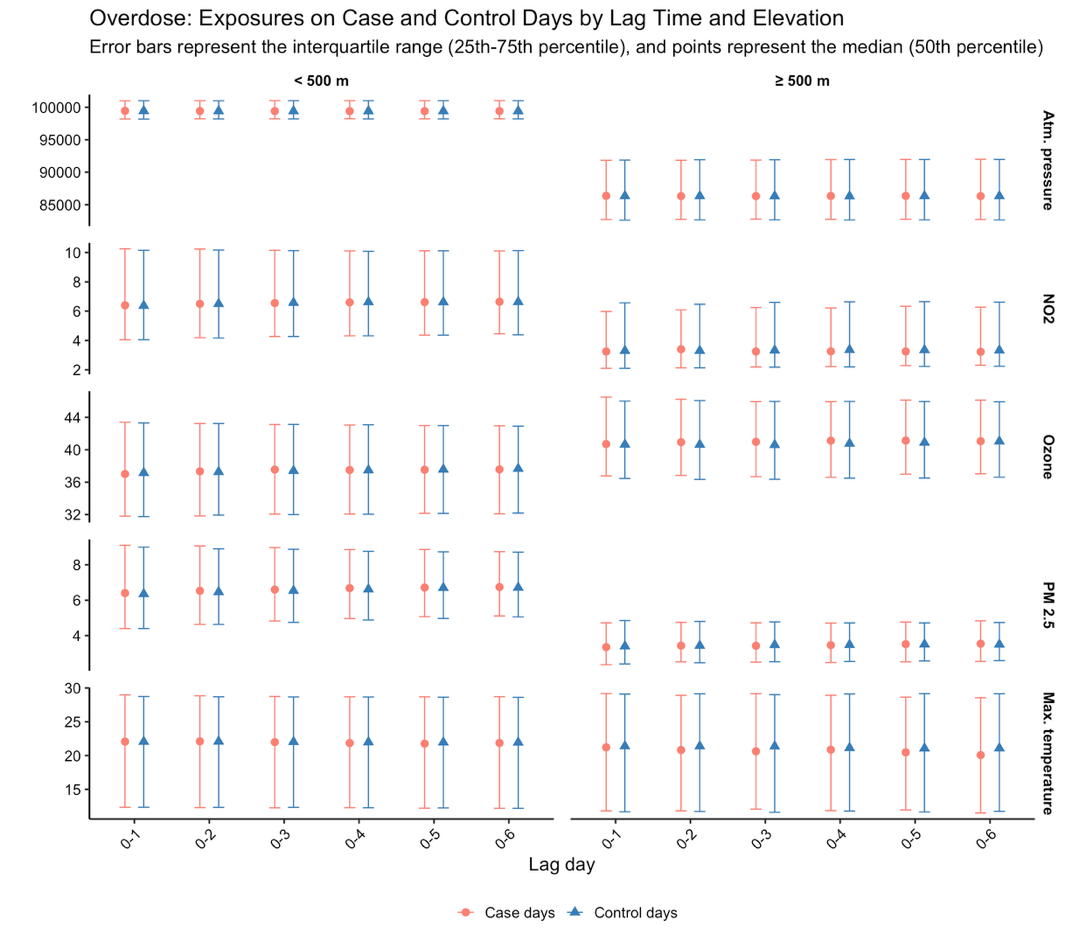* |
| *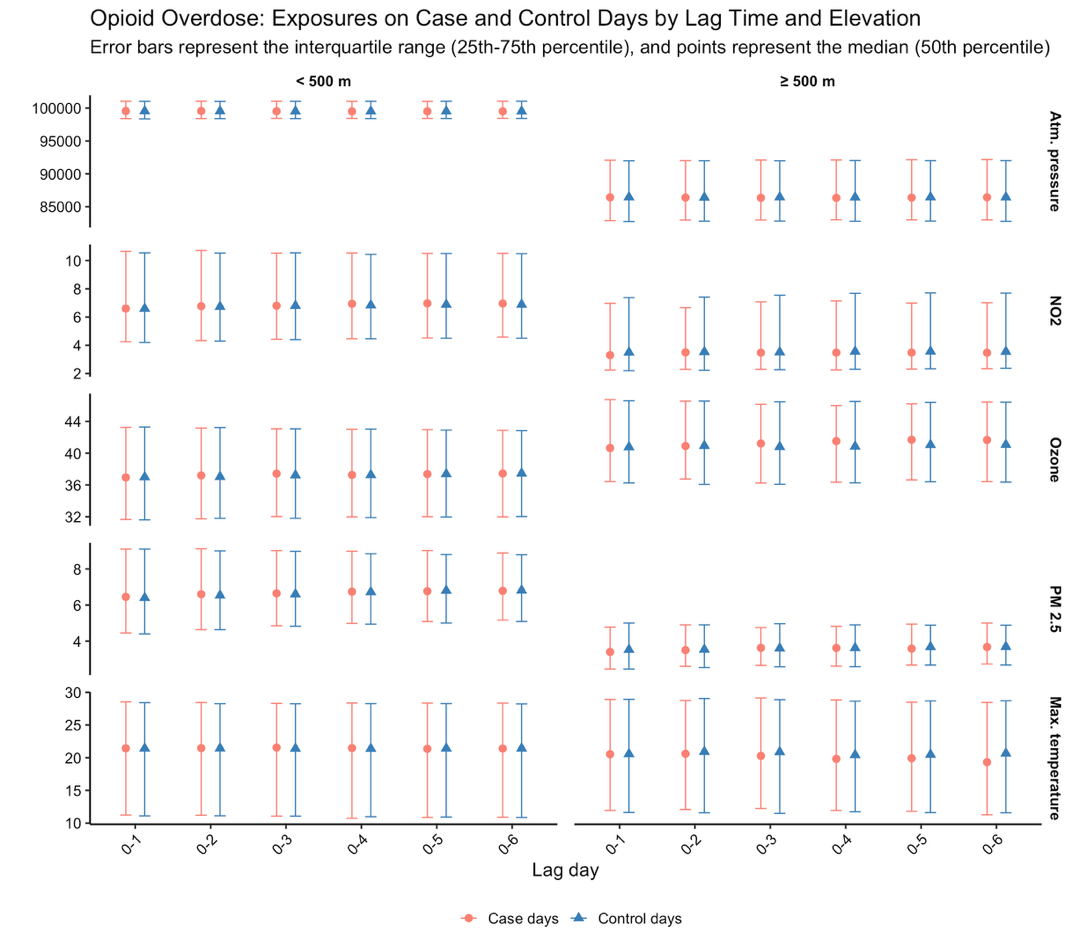* |

**Figure S2:** Odds ratios (ORs) and 95% confidence intervals (CIs) estimated for each exposure across *single lag days* (0 to 6) for suicide, drug overdose, and opioid overdose in the full population. Estimates with CIs that do not cross 1 are highlighted in bold, with blue indicating ORs < 1 and red indicating ORs > 1.

**
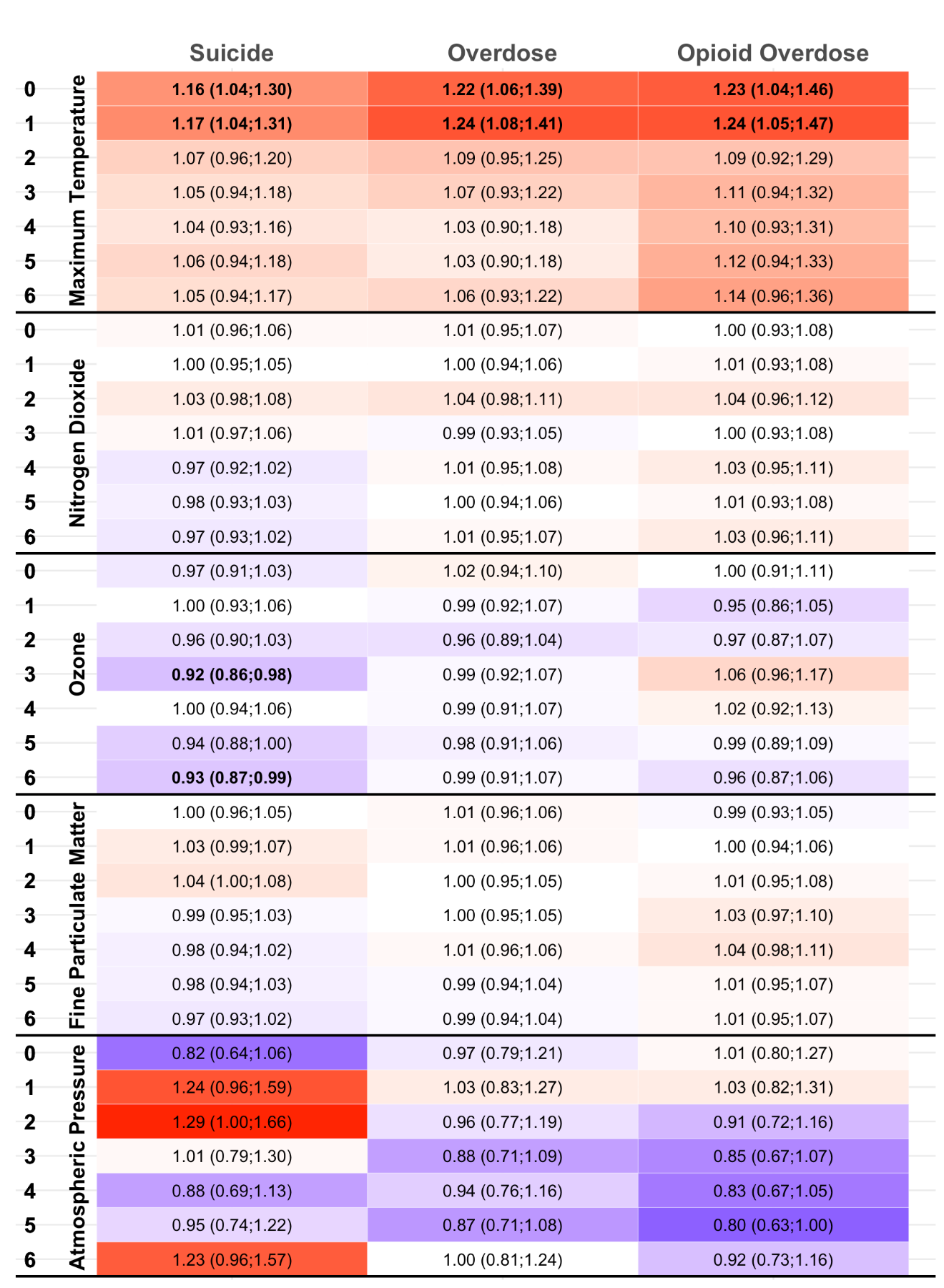
**

*Air pollution models (PM_2.5_, NO_2_, O_3_) were adjusted for maximum temperature, precipitation (mm/day), and shortwave solar radiation (W/m²). Maximum temperature models were adjusted for PM_2.5_, precipitation, and solar radiation. Atmospheric pressure models were adjusted for PM_2.5_, maximum temperature, precipitation, and solar radiation. Results are expressed as adjusted odds ratios (ORs) calculated per interquartile range (IQR) increase of each exposure.*

**Table S1:** Summary statistics of U.S. veterans’ suicide and overdose mortality (including opioid overdose), by U.S. Census Division, 2018–2019.

| **U.S. Region / Division** | **Suicide (N)** | **Overdose (N)** | **Opioid Overdose (N)** |
| --- | --- | --- | --- |
| **North Central** | **1,299** | **931** | **640** |
| East North Central | 795 | 705 | 525 |
| West North Central | 504 | 226 | 115 |
| **Northeast** | **655** | **850** | **655** |
| New England | 203 | 275 | 236 |
| Middle Atlantic | 452 | 575 | 419 |
| **South** | **2,734** | **1,629** | **1,034** |
| South Atlantic | 1,458 | 982 | 687 |
| East South Central | 471 | 298 | 202 |
| West South Central | 805 | 349 | 145 |
| **West** | **1,678** | **911** | **431** |
| Mountain | 843 | 378 | 209 |
| Pacific | 835 | 533 | 222 |
| **Total** | **6,366** | **4,321** | **2,760** |

**Table S2:** Summary statistics of air pollutant and meteorological exposures across *cumulative lag days* (0–1 to 0–6), by cause of death, from 2018 to 2019.

| Exposure | Cumulative Lag Days | **Suicide** | | **Overdose** | | **Opioid Overdose** | |
| --- | --- | --- | --- | --- | --- | --- | --- |
|  |  | Mean (SD) [IQR] | Mean (SD) [IQR] | Mean (SD) [IQR] | Mean (SD) [IQR] | Mean (SD) [IQR] | Mean (SD) [IQR] |
|  |  | case days | controls days | case days | controls days | case days | controls days |
| Average 1-hour Maximum  Nitrogen Dioxide  (NO_2_)  [ppb] | 0-1 | 6.04 (4.54) [4.70] | 6.04 (4.50) [4.70] | 7.77 (5.89) [6.29] | 7.73 (5.84) [6.20] | 8.13 (6.11) [6.49] | 8.07 (6.04) [6.45] |
|  | 0-2 | 6.05 (4.34) [4.60] | 6.04 (4.31) [4.60] | 7.78 (5.74) [6.15] | 7.72 (5.60) [6.03] | 8.14 (5.96) [6.40] | 8.07 (5.79) [6.31] |
|  | 0-3 | 6.05 (4.24) [4.48] | 6.04 (4.19) [4.53] | 7.78 (5.62) [5.95] | 7.73 (5.46) [5.98] | 8.14 (5.81) [6.20] | 8.08 (5.63) [6.22] |
|  | 0-4 | 6.05 (4.14) [4.38] | 6.05 (4.12) [4.46] | 7.78 (5.53) [5.88] | 7.73 (5.38) [5.82] | 8.14 (5.71) [6.19] | 8.08 (5.55) [6.10] |
|  | 0-5 | 6.04 (4.08) [4.30] | 6.05 (4.06) [4.42] | 7.77 (5.44) [5.88] | 7.73 (5.32) [5.85] | 8.13 (5.61) [6.15] | 8.07 (5.48) [6.08] |
|  | 0-6 | 6.04 (4.03) [4.30] | 6.06 (4.03) [4.38] | 7.76 (5.37) [5.85] | 7.72 (5.27) [5.79] | 8.13 (5.55) [6.10] | 8.05 (5.43) [6.09] |
| Average 8-hour  Maximum Ozone  (O_3_)  [ppb] | 0-1 | 38.43 (7.79) [11.10] | 38.42 (7.67) [10.90] | 38.17 (8.29) [11.49] | 38.11 (8.06) [11.55] | 37.97 (8.28) [11.35] | 37.97 (8.21) [11.700] |
|  | 0-2 | 38.42 (7.47) [10.70] | 38.42 (7.43) [10.70] | 38.18 (7.97) [11.33] | 38.14 (7.79) [11.17] | 38.01 (7.96) [11.43] | 38.00 (7.88) [11.30] |
|  | 0-3 | 38.40 (7.24) [10.66] | 38.44 (7.25) [10.55] | 38.20 (7.75) [11.02] | 38.15 (7.61) [11.00] | 38.05 (7.74) [11.11] | 38.00 (7.68) [11.17] |
|  | 0-4 | 38.41 (7.11) [10.42] | 38.43 (7.12) [10.44] | 38.17 (7.60) [10.92] | 38.14 (7.48) [10.88] | 38.05 (7.61) [10.95] | 37.99 (7.55) [11.08] |
|  | 0-5 | 38.39 (7.01) [10.34] | 38.44 (7.01) [10.31] | 38.16 (7.47) [10.80] | 38.14 (7.36) [10.71] | 38.05 (7.49) [10.96] | 37.98 (7.43) [10.93] |
|  | 0-6 | 38.39 (6.92) [10.24] | 38.45 (6.93) [10.26] | 38.16 (7.38) [10.81] | 38.13 (7.27) [10.66] | 38.03 (7.40) [10.88] | 37.98 (7.34) [10.80] |
| Average 24-hour  Fine Particulate  Matter  (PM_2.5_)  [µg/m^3^] | 0-1 | 6.34 (3.81) [4.35] | 6.29 (3.64) [4.35] | 6.92 (4.08) [4.69] | 6.82 (3.87) [4.60] | 6.99 (4.07) [4.60] | 6.93 (3.86) [4.68] |
|  | 0-2 | 6.37 (3.60) [4.15] | 6.29 (3.43) [4.16] | 6.92 (3.75) [4.49] | 6.82 (3.61) [4.33] | 7.00 (3.76) [4.47] | 6.93 (3.55) [4.40] |
|  | 0-3 | 6.36 (3.36) [4.01] | 6.30 (3.27) [4.00] | 6.91 (3.50) [4.27] | 6.82 (3.39) [4.18] | 7.01 (3.52) [4.25] | 6.92 (3.33) [4.17] |
|  | 0-4 | 6.35 (3.19) [3.86] | 6.31 (3.14) [3.90] | 6.89 (3.33) [4.04] | 6.82 (3.24) [4.04] | 7.03 (3.36) [4.10] | 6.93 (3.19) [4.00] |
|  | 0-5 | 6.34 (3.07) [3.75] | 6.32 (3.04) [3.78] | 6.89 (3.19) [3.97] | 6.82 (3.11) [3.92] | 7.04 (3.24) [4.06] | 6.93 (3.07) [3.88] |
|  | 0-6 | 6.33 (2.97) [3.70] | 6.32 (2.95) [3.70] | 6.89 (3.08) [3.80] | 6.82 (3.02) [3.80] | 7.03 (3.14) [3.84] | 6.92 (2.97) [3.81] |
| Average 24-hour  Precipitation  [mm/day] | 0-1 | 3.28 (6.31) [3.76] | 3.27 (6.47) [3.91] | 3.37 (6.21) [4.11] | 3.46 (6.26) [4.28] | 3.56 (6.46) [4.53] | 3.56 (6.18) [4.56] |
|  | 0-2 | 3.27 (5.40) [4.41] | 3.29 (5.56) [4.44] | 3.40 (5.27) [4.84] | 3.43 (5.28) [4.84] | 3.58 (5.45) [5.09] | 3.53 (5.18) [5.10] |
|  | 0-3 | 3.25 (4.80) [4.70] | 3.31 (4.93) [4.67] | 3.42 (4.69) [5.02] | 3.42 (4.70) [4.98] | 3.56 (4.68) [5.10] | 3.52 (4.56) [5.12] |
|  | 0-4 | 3.26 (4.48) [4.77] | 3.31 (4.53) [4.67] | 3.42 (4.23) [4.91] | 3.43 (4.29) [4.90] | 3.55 (4.15) [4.85] | 3.54 (4.17) [4.98] |
|  | 0-5 | 3.28 (4.29) [4.63] | 3.30 (4.22) [4.56] | 3.44 (3.96) [4.72] | 3.44 (4.00) [4.79] | 3.55 (3.86) [4.56] | 3.56 (3.89) [4.86] |
|  | 0-6 | 3.28 (4.06) [4.54] | 3.29 (3.99) [4.48] | 3.42 (3.71) [4.57] | 3.46 (3.80) [4.65] | 3.54 (3.62) [4.45] | 3.57 (3.68) [4.66] |
| Average 24-hour  Total Solar  Radiation  [MJ/m^2^/day] | 0-1 | 331.06 (107.74) [160.14] | 331.14 (108.49) [161.79] | 324.10 (108.11) [167.07] | 323.05 (107.63) [165.33] | 319.43 (106.11) [164.51] | 318.18 (106.73) [164.06] |
|  | 0-2 | 331.02 (103.08) [151.82] | 330.88 (103.28) [152.83] | 324.62 (103.01) [157.09] | 323.04 (102.23) [154.93] | 319.97 (100.62) [153.6] | 318.44 (101.38) [154.66] |
|  | 0-3 | 331.07 (99.49) [144.31] | 330.87 (99.79) [147.46] | 324.80 (99.48) [149.36] | 323.12 (98.45) [148.29] | 320.02 (96.83) [146.95] | 318.35 (97.31) [147.45] |
|  | 0-4 | 331.24 (96.77) [137.05] | 331.02 (97.25) [142.32] | 324.31 (96.82) [145.08] | 323.12 (95.80) [145.30] | 319.55 (94.39) [143.07] | 318.18 (94.39) [145.11] |
|  | 0-5 | 331.05 (95.03) [135.51] | 331.1 (95.41) [139.16] | 323.79 (95.06) [143.69] | 322.85 (93.81) [139.74] | 319.19 (92.92) [142.09] | 317.87 (92.29) [140.30] |
|  | 0-6 | 330.91 (93.55) [133.84] | 331.21 (94.06) [136.81] | 323.74 (93.67) [140.48] | 322.69 (92.37) [137.47] | 319.08 (91.48) [137.69] | 317.68 (90.86) [136.87] |
| Average 24-hour  Maximum  Temperature  [Degrees C] | 0-1 | 21.19 (10.76) [16.18] | 21.08 (10.69) [16.23] | 20.28 (10.63) [16.78] | 20.14 (10.67) [16.54] | 19.68 (10.77) [17.24] | 19.53 (10.83) [17.29] |
|  | 0-2 | 21.15 (10.65) [16.22] | 21.07 (10.58) [16.07] | 20.25 (10.58) [16.64] | 20.13 (10.57) [16.48] | 19.66 (10.72) [17.20] | 19.53 (10.72) [17.13] |
|  | 0-3 | 21.14 (10.57) [16.29] | 21.08 (10.49) [16.06] | 20.22 (10.52) [16.57] | 20.13 (10.50) [16.49] | 19.63 (10.66) [17.16] | 19.52 (10.64) [17.21] |
|  | 0-4 | 21.13 (10.51) [16.18] | 21.08 (10.43) [16.01] | 20.17 (10.49) [16.50] | 20.11 (10.45) [16.53] | 19.59 (10.63) [17.44] | 19.50 (10.59) [17.26] |
|  | 0-5 | 21.11 (10.47) [16.32] | 21.07 (10.39) [16.01] | 20.14 (10.47) [16.56] | 20.10 (10.41) [16.52] | 19.57 (10.61) [17.42] | 19.49 (10.55) [17.30] |
|  | 0-6 | 21.10 (10.43) [16.27] | 21.07 (10.36) [15.96] | 20.12 (10.44) [16.58] | 20.09 (10.37) [16.54] | 19.56 (10.58) [17.36] | 19.48 (10.52) [17.34] |
| Average 24-hour  Atmospheric  Pressure  [Pa] | 0-1 | 97,079 (5,422) [4,352] | 97,090 (5,410) [4,379] | 98,041 (4,583) [3,262] | 98,031 (4,585) [3,333] | 98,397 (4,241) [2,985] | 98,392 (4,229) [3,008] |
|  | 0-2 | 97,084 (5,419) [4,402] | 97,090 (5,406) [4,358] | 98,041 (4,575) [3,241] | 98,031 (4,578) [3,284] | 98,394 (4,228) [2,973] | 98,391 (4,219) [2,967] |
|  | 0-3 | 97,084 (5,416) [4,368] | 97,092 (5,404) [4,356] | 98,039 (4,570) [3,255] | 98,031 (4,574) [3,275] | 98,390 (4,222) [2,959] | 98,391 (4,213) [2,946] |
|  | 0-4 | 97,084 (5,414) [4,368] | 97,093 (5,403) [4,359] | 98,038 (4,568) [3,258] | 98,031 (4,570) [3,265] | 98,388 (4,218) [2,963] | 98,392 (4,209) [2,948] |
|  | 0-5 | 97,083 (5,413) [4,396] | 97,093 (5,401) [4,347] | 98,038 (4,565) [3,261] | 98,032 (4,568) [3,271] | 98,387 (4,216) [2,953] | 98,394 (4,207) [2,943] |
|  | 0-6 | 97,085 (5,412) [4,404] | 97,094 (5,400) [4,355] | 98,038 (4,564) [3,259] | 98,032 (4,567) [3,264] | 98,388 (4,216) [2,957] | 98,395 (4,205) [2,930] |

*IQR=Q3−Q1*

**Table S3:** Summary statistics of air pollutant and meteorological exposures across *single lag days* (0 to 6), by cause of death, from 2018 to 2019.

| Exposure | Single Lag Day | **Suicide** | | **Overdose** | | **Opioid Overdose** | |
| --- | --- | --- | --- | --- | --- | --- | --- |
|  |  | Mean (SD) [IQR] | Mean (SD) [IQR] | Mean (SD) [IQR] | Mean (SD) [IQR] | Mean (SD) [IQR] | Mean (SD) [IQR] |
|  |  | case days | controls days | case days | controls days | case days | controls days |
| Average 1-hour Maximum  Nitrogen Dioxide  (NO_2_)  [ppb] | 0 | 6.07 (4.86) [4.90] | 6.05 (4.81) [4.90] | 7.78 (6.20) [6.40] | 7.73 (6.21) [6.40] | 8.09 (6.41) [6.70] | 8.04 (6.41) [6.60] |
|  | 1 | 6.03 (4.84) [4.80] | 6.03 (4.79) [4.90] | 7.77 (6.36) [6.25] | 7.73 (6.23) [6.40] | 8.17 (6.65) [6.50] | 8.10 (6.49) [6.70] |
|  | 2 | 6.06 (4.78) [5.00] | 6.03 (4.75) [5.00] | 7.81 (6.41) [6.40] | 7.70 (6.15) [6.50] | 8.18 (6.68) [6.70] | 8.07 (6.39) [6.80] |
|  | 3 | 6.07 (4.87) [4.80] | 6.05 (4.81) [4.90] | 7.77 (6.46) [6.30] | 7.75 (6.22) [6.30] | 8.14 (6.64) [6.60] | 8.10 (6.43) [6.70] |
|  | 4 | 6.03 (4.69) [4.80] | 6.08 (4.81) [4.80] | 7.76 (6.36) [6.40] | 7.75 (6.26) [6.40] | 8.16 (6.63) [6.80] | 8.09 (6.50) [6.70] |
|  | 5 | 6.02 (4.79) [4.82] | 6.08 (4.81) [4.90] | 7.72 (6.27) [6.30] | 7.70 (6.26) [6.30] | 8.08 (6.50) [6.50] | 8.02 (6.43) [6.60] |
|  | 6 | 6.01 (4.70) [4.90] | 6.08 (4.80) [4.90] | 7.69 (6.15) [6.30] | 7.63 (6.13) [6.30] | 8.09 (6.49) [6.50] | 7.94 (6.36) [6.50] |
| Average 8-hour  Maximum Ozone  (O_3_)  [ppb] | 0 | 38.44 (8.27) [11.4] | 38.45 (8.15) [11.2] | 38.17 (8.83) [11.9] | 38.1 (8.59) [11.9] | 37.99 (8.79) [11.8] | 37.95 (8.75) [12.20] |
|  | 1 | 38.41 (8.22) [11.3] | 38.38 (8.16) [11.3] | 38.16 (8.81) [12.2] | 38.11 (8.64) [11.8] | 37.94 (8.89) [12.2] | 38.00 (8.80) [12.20] |
|  | 2 | 38.41 (8.13) [11.4] | 38.44 (8.23) [11.3] | 38.21 (8.77) [12.1] | 38.2 (8.68) [11.8] | 38.08 (8.83) [12.1] | 38.06 (8.75) [11.90] |
|  | 3 | 38.33 (8.15) [11.4] | 38.47 (8.24) [11.4] | 38.24 (8.7) [11.77] | 38.19 (8.67) [11.9] | 38.19 (8.71) [11.9] | 37.98 (8.73) [12.10] |
|  | 4 | 38.44 (8.2) [11.3] | 38.43 (8.24) [11.5] | 38.08 (8.68) [12] | 38.11 (8.71) [11.9] | 38.04 (8.79) [12.3] | 37.94 (8.84) [12.05] |
|  | 5 | 38.32 (8.3) [11.3] | 38.45 (8.21) [11.3] | 38.09 (8.6) [12.1] | 38.1 (8.57) [11.8] | 38.03 (8.73) [12.28] | 37.95 (8.69) [12.10] |
|  | 6 | 38.35 (8.17) [11.2] | 38.55 (8.26) [11.5] | 38.13 (8.67) [11.9] | 38.12 (8.64) [11.9] | 37.96 (8.75) [11.87] | 37.96 (8.70) [12.10] |
| Average 24-hour  Fine Particulate  Matter  (PM_2.5_)  [µg/m^3^] | 0 | 6.33 (4.12) [4.8] | 6.3 (3.97) [4.7] | 6.89 (4.53) [5.1] | 6.8 (4.25) [5.1] | 6.94 (4.49) [5.2] | 6.90 (4.25) [5.20] |
|  | 1 | 6.36 (4.19) [4.8] | 6.28 (4) [4.8] | 6.94 (4.4) [5.17] | 6.83 (4.26) [5.1] | 7.03 (4.44) [5.2] | 6.95 (4.28) [5.30] |
|  | 2 | 6.41 (4.31) [4.8] | 6.31 (4.13) [4.8] | 6.92 (4.33) [5.3] | 6.84 (4.32) [5.1] | 7.03 (4.43) [5.3] | 6.93 (4.26) [5.30] |
|  | 3 | 6.34 (4) [4.7] | 6.33 (4.14) [4.8] | 6.87 (4.33) [5] | 6.83 (4.25) [5.2] | 7.05 (4.46) [5.2] | 6.91 (4.27) [5.30] |
|  | 4 | 6.3 (3.97) [4.8] | 6.33 (4.12) [4.7] | 6.85 (4.28) [5] | 6.82 (4.3) [5.1] | 7.08 (4.45) [5.2] | 6.95 (4.39) [5.20] |
|  | 5 | 6.31 (4.09) [4.7] | 6.36 (4.12) [4.8] | 6.88 (4.38) [5.2] | 6.79 (4.27) [5.1] | 7.09 (4.57) [5.4] | 6.91 (4.32) [5.20] |
|  | 6 | 6.29 (3.94) [4.8] | 6.36 (4.1) [4.8] | 6.85 (4.28) [5.08] | 6.83 (4.4) [5] | 7.01 (4.41) [5.2] | 6.91 (4.42) [5.00] |
| Average 24-hour  Precipitation  [mm/day] | 0 | 3.27 (8.21) [2.58] | 3.28 (8.17) [2.45] | 3.48 (8.29) [2.89] | 3.49 (8.14) [2.88] | 3.74 (8.8) [3.18] | 3.62 (8.15) [3.18] |
|  | 1 | 3.29 (7.78) [2.81] | 3.25 (7.96) [2.62] | 3.27 (7.49) [2.93] | 3.43 (7.84) [2.96] | 3.38 (7.68) [3.24] | 3.50 (7.7) [3.19] |
|  | 2 | 3.25 (7.88) [2.58] | 3.33 (8.3) [2.74] | 3.44 (8.05) [2.77] | 3.38 (7.81) [2.88] | 3.63 (8.28) [3.07] | 3.46 (7.67) [3.10] |
|  | 3 | 3.20 (7.80) [2.47] | 3.37 (8.03) [2.80] | 3.48 (8.01) [2.99] | 3.39 (7.93) [2.93] | 3.50 (7.55) [3.20] | 3.51 (7.91) [3.18] |
|  | 4 | 3.31 (8.35) [2.42] | 3.30 (8.09) [2.52] | 3.43 (7.85) [3.00] | 3.44 (8.01) [2.84] | 3.48 (7.57) [3.26] | 3.61 (8.16) [3.22] |
|  | 5 | 3.36 (8.37) [2.80] | 3.25 (7.95) [2.54] | 3.55 (8.13) [3.27] | 3.52 (8.11) [3.12] | 3.56 (8.04) [3.43] | 3.67 (8.21) [3.42] |
|  | 6 | 3.32 (8.23) [2.67] | 3.28 (8.15) [2.49] | 3.32 (7.51) [2.81] | 3.55 (8.61) [2.94] | 3.52 (7.67) [3.14] | 3.65 (8.28) [3.22] |
| Average 24-hour  Total Solar  Radiation  [MJ/m^2^/day] | 0 | 331.67 (114.96) [170.76] | 331.46 (115.69) [172.52] | 323.31 (116.10) [178.03] | 323.11 (115.87) [176.43] | 318.90 (115.38) [176.09] | 317.88 (115.55) [176.02] |
|  | 1 | 330.44 (115.49) [172.77] | 330.81 (116.03) [174.30] | 324.88 (115.51) [176.72] | 322.98 (115.33) [176.99] | 319.95 (113.51) [171.66] | 318.48 (114.64) [175.52] |
|  | 2 | 330.94 (115.96) [174.38] | 330.36 (115.88) [174.76] | 325.67 (115.85) [172.86] | 323.03 (114.98) [174.09] | 321.05 (113.53) [170.09] | 318.95 (114.98) [173.61] |
|  | 3 | 331.23 (115.10) [171.47] | 330.85 (116.26) [175.48] | 325.34 (116.44) [178.40] | 323.34 (115.27) [176.58] | 320.16 (115.26) [176.54] | 318.09 (114.60) [174.14] |
|  | 4 | 331.93 (114.07) [167.31] | 331.64 (115.76) [173.61] | 322.33 (115.19) [174.12] | 323.13 (115.17) [174.48] | 317.70 (115.35) [173.35] | 317.51 (114.17) [174.93] |
|  | 5 | 330.11 (115.53) [172.55] | 331.51 (115.70) [173.42] | 321.23 (117.16) [180.24] | 321.52 (115.35) [176.16] | 317.37 (116.69) [177.46] | 316.29 (114.85) [176.96] |
|  | 6 | 330.05 (115.64) [172.83] | 331.85 (116.11) [174.53] | 323.45 (115.92) [175.64] | 321.70 (115.32) [175.15] | 318.44 (114.48) [173.33] | 316.53 (115.09) [174.43] |
| Average 24-hour  Maximum  Temperature  [Degrees C] | 0 | 21.22 (10.9) [16.22] | 21.11 (10.81) [16.39] | 20.25 (10.76) [16.97] | 20.14 (10.82) [16.66] | 19.66 (10.89) [17.44] | 19.52 (10.99) [17.44] |
|  | 1 | 21.15 (10.91) [16.55] | 21.04 (10.87) [16.49] | 20.3 (10.82) [16.79] | 20.13 (10.84) [16.76] | 19.70 (10.98) [17.54] | 19.54 (10.99) [17.51] |
|  | 2 | 21.09 (10.92) [16.53] | 21.06 (10.82) [16.35] | 20.2 (10.99) [16.93] | 20.12 (10.87) [16.92] | 19.62 (11.17) [17.74] | 19.53 (11.02) [17.48] |
|  | 3 | 21.1 (10.92) [16.69] | 21.09 (10.81) [16.46] | 20.14 (11.00) [17.10] | 20.11 (10.91) [16.90] | 19.56 (11.14) [17.83] | 19.48 (11.04) [17.66] |
|  | 4 | 21.08 (10.91) [16.57] | 21.07 (10.86) [16.45] | 19.99 (11.05) [17.18] | 20.06 (10.94) [16.96] | 19.44 (11.20) [18.32] | 19.42 (11.10) [17.73] |
|  | 5 | 21.02 (10.96) [16.60] | 21.04 (10.89) [16.48] | 19.97 (11.08) [17.47] | 20.04 (10.95) [17.09] | 19.45 (11.22) [18.09] | 19.42 (11.10) [17.74] |
|  | 6 | 21.04 (10.94) [16.56] | 21.08 (10.92) [16.46] | 20.03 (11.00) [17.25] | 20.05 (10.93) [17.21] | 19.51 (11.15) [17.58] | 19.43 (11.08) [17.85] |
| Average 24-hour  Atmospheric  Pressure  [Pa] | 0 | 97,073 (5,421) [4,426] | 97,096 (5,416) [4,441] | 98,037 (4,595) [3,326] | 98,031 (4,592) [3,337] | 98,395 (4,253) [3,004] | 98,394 (4,237) [3,037] |
|  | 1 | 97,085 (5,434) [4,416] | 97,084 (5,414) [4,424] | 98,045 (4,587) [3,302] | 98,031 (4,593) [3,337] | 98,398 (4,247) [2,981] | 98,391 (4,240) [3,014] |
|  | 2 | 97,094 (5,431) [4,443] | 97,089 (5,418) [4,413] | 98,042 (4,585) [3,331] | 98,030 (4,590) [3,335] | 98,389 (4,235) [3,004] | 98,388 (4,230) [3,023] |
|  | 3 | 97,085 (5,427) [4,403] | 97,096 (5,419) [4,414] | 98,032 (4,585) [3,321] | 98,032 (4,590) [3,337] | 98,379 (4,236) [2,950] | 98,391 (4,229) [3,011] |
|  | 4 | 97,081 (5,428) [4,414] | 97,098 (5,418) [4,395] | 98,034 (4,587) [3,373] | 98,031 (4,586) [3,329] | 98,379 (4,238) [2,996] | 98,396 (4,230) [2,997] |
|  | 5 | 97,081 (5,429) [4,469] | 97,096 (5,416) [4,420] | 98,035 (4,582) [3,370] | 98,035 (4,589) [3,355] | 98,385 (4,242) [3,006] | 98,403 (4,235) [2,988] |
|  | 6 | 97,092 (5,433) [4,463] | 97,096 (5,415) [4,442] | 98,041 (4,593) [3,330] | 98,034 (4,590) [3,333] | 98,395 (4,255) [2,999] | 98,402 (4,229) [2,982] |

*IQR=Q3−Q1*

**Table S4:** Adjusted odds ratios (ORs) and 95% confidence intervals (CIs) for exposures across *cumulative lag days* (0–1 to 0–6) from *suicide*-stratified models, presented by season, region, rural/urban classification, and elevation. Estimates with CIs that do not cross 1 are highlighted in bold.

|  |  |  | | | | **Suicide** | | | |  | | | |
| --- | --- | --- | --- | --- | --- | --- | --- | --- | --- | --- | --- | --- | --- |
|  |  | **Season** | | | | **Region** | | | | **Urbanicity** | | **Altitude** | |
|  |  | **Spring** | **Summer** | **Fall** | **Winter** | **North Central** | **Northeast** | **South** | **West** | **Metro** | **Nonmetro** | **< 500 m** | **>= 500 m** |
| **Nitrogen Dioxide (NO_2_)** | **Lag**  **0-1** | 0.97 (0.87;1.09) | 1.04 (0.92;1.19) | **0.85 (0.75;0.96)** | **1.19 (1.07;1.32)** | **1.11 (1.00;1.24)** | 0.97 (0.83;1.14) | 1.01 (0.94;1.09) | 0.95 (0.82;1.09) | 1.02 (0.96;1.08) | 1.04 (0.92;1.17) | 1.03 (0.98;1.09) | 0.93 (0.80;1.09) |
|  | **Lag**  **0-2** | 0.99 (0.87;1.13) | 0.99 (0.85;1.14) | **0.85 (0.74;0.98)** | **1.27 (1.13;1.43)** | **1.11 (1.00;1.24)** | 0.96 (0.82;1.13) | 1.01 (0.94;1.09) | 0.95 (0.83;1.09) | 1.02 (0.96;1.08) | 1.04 (0.92;1.17) | 1.03 (0.98;1.09) | 0.93 (0.80;1.09) |
|  | **Lag**  **0-3** | 0.99 (0.86;1.14) | 0.94 (0.80;1.11) | 0.88 (0.76;1.02) | **1.29 (1.13;1.47)** | 1.11 (0.99;1.23) | 0.96 (0.82;1.13) | 1.01 (0.93;1.09) | 0.95 (0.83;1.09) | 1.02 (0.96;1.07) | 1.04 (0.92;1.17) | 1.03 (0.97;1.08) | 0.93 (0.80;1.09) |
|  | **Lag**  **0-4** | 0.99 (0.85;1.15) | 0.92 (0.77;1.09) | 0.88 (0.75;1.03) | **1.25 (1.08;1.44)** | 1.11 (0.99;1.23) | 0.96 (0.81;1.12) | 1.01 (0.93;1.08) | 0.96 (0.83;1.10) | 1.01 (0.96;1.07) | 1.03 (0.92;1.17) | 1.03 (0.97;1.08) | 0.93 (0.80;1.08) |
|  | **Lag**  **0-5** | 1.01 (0.86;1.19) | 0.86 (0.72;1.04) | 0.86 (0.73;1.02) | **1.25 (1.07;1.47)** | 1.10 (0.99;1.23) | 0.96 (0.82;1.12) | 1.01 (0.93;1.08) | 0.96 (0.84;1.10) | 1.02 (0.96;1.07) | 1.03 (0.92;1.17) | 1.03 (0.97;1.08) | 0.93 (0.80;1.09) |
|  | **Lag**  **0-6** | 0.99 (0.83;1.18) | **0.79 (0.64;0.96)** | 0.85 (0.71;1.02) | **1.28 (1.09;1.52)** | 1.10 (0.99;1.23) | 0.96 (0.82;1.13) | 1.01 (0.93;1.08) | 0.96 (0.83;1.10) | 1.02 (0.96;1.07) | 1.04 (0.92;1.17) | 1.03 (0.97;1.08) | 0.93 (0.80;1.09) |
| **Ozone**  **(O_3_)** | **Lag**  **0-1** | 1.03 (0.92;1.15) | 0.96 (0.86;1.08) | 1.11 (0.98;1.27) | **0.84 (0.75;0.95)** | 1.01 (0.86;1.18) | 0.94 (0.78;1.13) | 1.01 (0.91;1.13) | 0.91 (0.78;1.06) | 0.98 (0.91;1.06) | 0.97 (0.83;1.14) | 0.98 (0.91;1.06) | 0.96 (0.79;1.16) |
|  | **Lag**  **0-2** | 1.01 (0.90;1.13) | 0.93 (0.82;1.05) | **1.18 (1.02;1.36)** | **0.81 (0.71;0.92)** | 0.99 (0.85;1.16) | 0.92 (0.76;1.10) | 1.01 (0.91;1.12) | 0.92 (0.79;1.06) | 0.98 (0.90;1.05) | 0.98 (0.84;1.14) | 0.98 (0.91;1.05) | 0.96 (0.79;1.17) |
|  | **Lag**  **0-3** | 1.02 (0.91;1.15) | **0.88 (0.77;1.00)** | **1.18 (1.01;1.39)** | **0.76 (0.66;0.87)** | 0.99 (0.84;1.15) | 0.91 (0.76;1.09) | 1.01 (0.91;1.12) | 0.93 (0.80;1.07) | 0.97 (0.90;1.05) | 0.98 (0.84;1.14) | 0.98 (0.91;1.05) | 0.97 (0.80;1.17) |
|  | **Lag**  **0-4** | 1.06 (0.94;1.20) | **0.87 (0.76;0.99)** | **1.21 (1.03;1.44)** | **0.73 (0.63;0.85)** | 0.99 (0.84;1.15) | 0.92 (0.77;1.10) | 1.01 (0.91;1.11) | 0.93 (0.80;1.07) | 0.97 (0.90;1.05) | 0.98 (0.84;1.15) | 0.98 (0.91;1.05) | 0.97 (0.80;1.17) |
|  | **Lag**  **0-5** | 1.08 (0.95;1.22) | **0.84 (0.72;0.97)** | 1.17 (0.98;1.41) | **0.72 (0.62;0.84)** | 0.98 (0.84;1.15) | 0.93 (0.79;1.11) | 1.00 (0.91;1.11) | 0.93 (0.80;1.07) | 0.97 (0.90;1.05) | 0.99 (0.85;1.15) | 0.98 (0.91;1.05) | 0.97 (0.80;1.17) |
|  | **Lag**  **0-6** | 1.09 (0.96;1.24) | **0.81 (0.70;0.94)** | 1.14 (0.94;1.38) | **0.71 (0.61;0.83)** | 0.98 (0.84;1.14) | 0.93 (0.79;1.11) | 1.01 (0.91;1.12) | 0.93 (0.80;1.08) | 0.97 (0.90;1.05) | 0.99 (0.85;1.15) | 0.98 (0.91;1.05) | 0.97 (0.81;1.18) |
| **Fine Particulate Matter**  **(PM_2.5_)** | **Lag**  **0-1** | 1.08 (0.98;1.19) | 0.97 (0.88;1.06) | **0.91 (0.83;0.99)** | **1.15 (1.05;1.26)** | 1.04 (0.93;1.16) | 0.96 (0.83;1.11) | 1.01 (0.93;1.09) | 0.98 (0.90;1.07) | 1.03 (0.98;1.09) | **0.89 (0.79;1.00)** | 1.01 (0.96;1.07) | 0.92 (0.81;1.05) |
|  | **Lag**  **0-2** | 1.05 (0.95;1.17) | 0.96 (0.88;1.05) | 0.95 (0.87;1.04) | **1.20 (1.09;1.32)** | 1.04 (0.94;1.16) | 0.95 (0.82;1.09) | 1.01 (0.93;1.09) | 0.99 (0.91;1.08) | 1.03 (0.98;1.09) | **0.90 (0.80;1.00)** | 1.01 (0.96;1.07) | 0.93 (0.82;1.05) |
|  | **Lag**  **0-3** | 1.06 (0.95;1.19) | 0.94 (0.85;1.04) | 0.96 (0.88;1.06) | **1.15 (1.04;1.28)** | 1.04 (0.94;1.16) | 0.95 (0.83;1.09) | 1.00 (0.93;1.08) | 0.99 (0.91;1.08) | 1.03 (0.97;1.08) | 0.90 (0.80;1.01) | 1.01 (0.96;1.06) | 0.93 (0.82;1.06) |
|  | **Lag**  **0-4** | 1.10 (0.97;1.24) | 0.92 (0.83;1.02) | 0.95 (0.86;1.05) | 1.11 (0.99;1.25) | 1.04 (0.94;1.16) | 0.94 (0.82;1.08) | 1.00 (0.93;1.07) | 0.99 (0.91;1.08) | 1.02 (0.97;1.08) | 0.90 (0.81;1.01) | 1.01 (0.96;1.06) | 0.93 (0.82;1.05) |
|  | **Lag**  **0-5** | 1.11 (0.98;1.26) | 0.91 (0.82;1.02) | 0.93 (0.84;1.04) | 1.12 (0.99;1.26) | 1.04 (0.93;1.15) | 0.94 (0.82;1.08) | 1.00 (0.93;1.07) | 0.99 (0.91;1.08) | 1.02 (0.97;1.08) | 0.90 (0.81;1.01) | 1.01 (0.96;1.06) | 0.94 (0.83;1.06) |
|  | **Lag**  **0-6** | 1.14 (0.99;1.30) | 0.91 (0.81;1.02) | 0.92 (0.82;1.02) | 1.10 (0.97;1.25) | 1.04 (0.94;1.15) | 0.95 (0.83;1.09) | 1.00 (0.93;1.08) | 1.00 (0.91;1.08) | 1.03 (0.97;1.08) | 0.91 (0.81;1.01) | 1.01 (0.96;1.06) | 0.94 (0.83;1.06) |
| **Maximum Temperature** | **Lag**  **0-1** | 1.18 (0.99;1.41) | **1.55 (1.35;1.79)** | 1.11 (0.90;1.37) | 0.97 (0.83;1.14) | 0.93 (0.63;1.38) | 1.56 (0.91;2.68) | 1.04 (0.86;1.27) | **1.57 (1.08;2.30)** | **1.22 (1.02;1.45)** | 0.79 (0.55;1.12) | 1.07 (0.91;1.27) | 1.45 (0.91;2.31) |
|  | **Lag**  **0-2** | 1.18 (0.98;1.43) | **1.61 (1.39;1.87)** | 1.17 (0.94;1.46) | 0.91 (0.77;1.08) | 0.90 (0.62;1.32) | 1.43 (0.85;2.40) | 1.04 (0.85;1.26) | **1.53 (1.07;2.19)** | **1.20 (1.01;1.42)** | 0.80 (0.57;1.13) | 1.06 (0.90;1.25) | 1.41 (0.91;2.18) |
|  | **Lag**  **0-3** | 1.15 (0.95;1.41) | **1.73 (1.48;2.03)** | 1.19 (0.94;1.49) | 0.89 (0.75;1.06) | 0.90 (0.63;1.31) | 1.40 (0.84;2.32) | 1.03 (0.85;1.24) | **1.59 (1.12;2.26)** | **1.18 (1.00;1.40)** | 0.82 (0.58;1.15) | 1.06 (0.90;1.24) | 1.41 (0.92;2.17) |
|  | **Lag**  **0-4** | 1.10 (0.90;1.36) | **1.87 (1.59;2.21)** | 1.20 (0.94;1.52) | 0.88 (0.73;1.05) | 0.91 (0.63;1.31) | 1.36 (0.82;2.24) | 1.02 (0.84;1.23) | **1.59 (1.12;2.25)** | **1.18 (1.00;1.39)** | 0.82 (0.59;1.14) | 1.06 (0.90;1.24) | 1.38 (0.90;2.11) |
|  | **Lag**  **0-5** | 1.08 (0.87;1.34) | **2.01 (1.69;2.39)** | 1.20 (0.94;1.54) | 0.88 (0.73;1.07) | 0.90 (0.63;1.29) | 1.34 (0.82;2.19) | 1.02 (0.85;1.23) | **1.59 (1.13;2.25)** | **1.18 (1.00;1.39)** | 0.82 (0.59;1.15) | 1.06 (0.91;1.24) | 1.39 (0.92;2.12) |
|  | **Lag**  **0-6** | 1.08 (0.87;1.35) | **2.12 (1.76;2.55)** | 1.22 (0.94;1.57) | 0.89 (0.73;1.09) | 0.91 (0.63;1.30) | 1.34 (0.82;2.19) | 1.03 (0.85;1.24) | **1.58 (1.12;2.23)** | **1.19 (1.01;1.40)** | 0.83 (0.59;1.15) | 1.07 (0.91;1.25) | 1.41 (0.93;2.13) |
| **Atmospheric Pressure** | **Lag**  **0-1** | 0.87 (0.46;1.63) | 0.74 (0.34;1.58) | 0.62 (0.38;1.02) | **2.04 (1.17;3.55)** | 1.06 (0.88;1.27) | 1.37 (0.84;2.25) | 0.96 (0.72;1.26) | 0.54 (0.09;3.26) | 1.14 (0.83;1.57) | 0.69 (0.40;1.18) | 1.06 (0.86;1.30) | 0.45 (0.07;2.82) |
|  | **Lag**  **0-2** | 0.84 (0.41;1.73) | 0.92 (0.39;2.16) | 0.86 (0.50;1.48) | **2.49 (1.30;4.76)** | 1.09 (0.89;1.34) | 1.57 (0.91;2.72) | 1.08 (0.79;1.49) | 0.77 (0.10;5.80) | 1.30 (0.91;1.86) | 0.86 (0.46;1.59) | 1.17 (0.92;1.48) | 0.79 (0.10;6.19) |
|  | **Lag**  **0-3** | 0.82 (0.37;1.83) | 1.06 (0.42;2.67) | 0.88 (0.48;1.61) | **2.57 (1.22;5.43)** | 1.10 (0.88;1.38) | 1.39 (0.76;2.55) | 1.16 (0.81;1.65) | 0.75 (0.08;7.00) | 1.29 (0.86;1.93) | 0.89 (0.44;1.78) | 1.15 (0.88;1.51) | 1.04 (0.11;10.2) |
|  | **Lag**  **0-4** | 0.66 (0.27;1.58) | 1.14 (0.42;3.10) | 0.79 (0.41;1.53) | **2.55 (1.11;5.84)** | 1.09 (0.86;1.40) | 1.15 (0.58;2.25) | 1.19 (0.80;1.77) | 0.68 (0.06;7.80) | 1.24 (0.80;1.92) | 0.79 (0.37;1.67) | 1.11 (0.83;1.49) | 0.78 (0.06;9.43) |
|  | **Lag**  **0-5** | 0.46 (0.18;1.19) | 1.59 (0.54;4.71) | 0.72 (0.35;1.49) | **2.72 (1.12;6.61)** | 1.06 (0.82;1.37) | 0.92 (0.44;1.94) | 1.25 (0.82;1.92) | 0.88 (0.06;12.1) | 1.22 (0.76;1.96) | 0.75 (0.34;1.69) | 1.10 (0.80;1.51) | 0.86 (0.06;12.6) |
|  | **Lag**  **0-6** | 0.37 (0.14;1.02) | 2.28 (0.71;7.33) | 0.76 (0.35;1.64) | **4.17 (1.59;10.9)** | 1.07 (0.81;1.41) | 0.92 (0.41;2.03) | 1.40 (0.89;2.21) | 1.02 (0.06;16.7) | 1.35 (0.82;2.24) | 0.82 (0.35;1.92) | 1.20 (0.85;1.67) | 0.98 (0.06;16.9) |

*Air pollution models (PM_2.5_, NO_2_, O_3_) were adjusted for maximum temperature, precipitation (mm/day), and shortwave solar radiation (W/m²). Maximum temperature models were adjusted for PM_2.5_, precipitation, and solar radiation. Atmospheric pressure models were adjusted for PM_2.5_, maximum temperature, precipitation, and solar radiation. Results are expressed as adjusted odds ratios (ORs) calculated per interquartile range (IQR) increase of each exposure.*

**Table S5:** Adjusted odds ratios (ORs) and 95% confidence intervals (CIs) for exposures across *cumulative lag days* (0–1 to 0–6) from *overdose*-stratified models, presented by season, region, rural/urban classification, and elevation. Estimates with CIs that do not cross 1 are highlighted in bold.

|  |  |  | | | | **Overdose** | | | |  | | | |
| --- | --- | --- | --- | --- | --- | --- | --- | --- | --- | --- | --- | --- | --- |
|  |  | **Season** | | | | **Region** | | | | **Urbanicity** | | **Altitude** | |
|  |  | **Spring** | **Summer** | **Fall** | **Winter** | **North Central** | **Northeast** | **South** | **West** | **Metro** | **Nonmetro** | **< 500 m** | **>= 500 m** |
| **Nitrogen Dioxide (NO_2_)** | **Lag**  **0-1** | 0.99 (0.86;1.15) | 0.87 (0.74;1.02) | 0.99 (0.85;1.15) | 1.09 (0.95;1.25) | 1.08 (0.94;1.23) | 1.03 (0.88;1.20) | 1.05 (0.95;1.17) | 0.95 (0.80;1.12) | 1.01 (0.95;1.09) | **1.26 (1.06;1.51)** | 1.03 (0.97;1.10) | 0.95 (0.75;1.22) |
|  | **Lag**  **0-2** | 1.03 (0.88;1.22) | 0.86 (0.72;1.03) | 1.06 (0.89;1.25) | 1.11 (0.95;1.29) | 1.08 (0.95;1.24) | 1.02 (0.87;1.19) | 1.06 (0.95;1.18) | 0.95 (0.80;1.12) | 1.02 (0.95;1.09) | **1.26 (1.05;1.50)** | 1.04 (0.97;1.10) | 0.95 (0.75;1.21) |
|  | **Lag**  **0-3** | 0.97 (0.81;1.17) | **0.80 (0.66;0.98)** | 1.10 (0.91;1.32) | 1.16 (0.97;1.38) | 1.09 (0.96;1.25) | 1.02 (0.87;1.19) | 1.06 (0.95;1.18) | 0.95 (0.80;1.12) | 1.02 (0.95;1.09) | **1.25 (1.05;1.49)** | 1.04 (0.97;1.11) | 0.95 (0.74;1.21) |
|  | **Lag**  **0-4** | 0.99 (0.81;1.22) | **0.75 (0.60;0.93)** | 1.11 (0.91;1.35) | **1.21 (1.00;1.47)** | 1.10 (0.96;1.25) | 1.02 (0.87;1.19) | 1.06 (0.96;1.18) | 0.95 (0.80;1.12) | 1.02 (0.96;1.09) | **1.25 (1.05;1.49)** | 1.04 (0.97;1.11) | 0.94 (0.74;1.20) |
|  | **Lag**  **0-5** | 1.01 (0.82;1.26) | **0.74 (0.58;0.93)** | 1.09 (0.88;1.35) | **1.24 (1.01;1.52)** | 1.10 (0.96;1.26) | 1.02 (0.87;1.19) | 1.06 (0.96;1.18) | 0.95 (0.80;1.12) | 1.02 (0.96;1.09) | **1.25 (1.04;1.49)** | 1.04 (0.97;1.11) | 0.94 (0.74;1.20) |
|  | **Lag**  **0-6** | 1.01 (0.80;1.28) | **0.70 (0.54;0.91)** | 1.05 (0.83;1.32) | **1.32 (1.05;1.64)** | 1.10 (0.97;1.26) | 1.02 (0.87;1.19) | 1.06 (0.96;1.18) | 0.94 (0.79;1.11) | 1.02 (0.96;1.09) | **1.24 (1.04;1.48)** | 1.04 (0.98;1.11) | 0.94 (0.74;1.19) |
| **Ozone**  **(O_3_)** | **Lag**  **0-1** | 1.01 (0.88;1.16) | 1.03 (0.88;1.19) | 1.10 (0.95;1.29) | 0.88 (0.77;1.01) | 0.96 (0.78;1.17) | 0.94 (0.80;1.11) | 1.10 (0.96;1.27) | 0.92 (0.75;1.13) | 1.00 (0.92;1.09) | 1.04 (0.81;1.34) | 1.01 (0.92;1.10) | 1.06 (0.77;1.44) |
|  | **Lag**  **0-2** | 1.01 (0.88;1.17) | 1.01 (0.87;1.18) | 1.09 (0.92;1.29) | **0.82 (0.70;0.95)** | 0.95 (0.78;1.15) | 0.95 (0.81;1.11) | 1.10 (0.96;1.26) | 0.92 (0.75;1.12) | 1.01 (0.92;1.09) | 1.03 (0.80;1.32) | 1.00 (0.92;1.09) | 1.05 (0.77;1.42) |
|  | **Lag**  **0-3** | 1.05 (0.91;1.21) | 0.97 (0.83;1.15) | 1.11 (0.92;1.34) | **0.79 (0.67;0.93)** | 0.96 (0.79;1.16) | 0.97 (0.83;1.13) | 1.10 (0.96;1.25) | 0.93 (0.76;1.13) | 1.01 (0.93;1.10) | 1.02 (0.79;1.31) | 1.01 (0.93;1.10) | 1.05 (0.78;1.43) |
|  | **Lag**  **0-4** | 1.06 (0.91;1.23) | 0.97 (0.81;1.16) | 1.15 (0.94;1.41) | **0.74 (0.62;0.88)** | 0.96 (0.79;1.16) | 0.97 (0.83;1.13) | 1.10 (0.96;1.26) | 0.94 (0.77;1.14) | 1.02 (0.94;1.11) | 1.02 (0.80;1.31) | 1.01 (0.93;1.10) | 1.06 (0.78;1.44) |
|  | **Lag**  **0-5** | 1.10 (0.94;1.27) | 0.94 (0.78;1.13) | 1.16 (0.94;1.44) | **0.73 (0.61;0.88)** | 0.97 (0.80;1.17) | 0.97 (0.84;1.13) | 1.10 (0.96;1.25) | 0.94 (0.77;1.14) | 1.02 (0.94;1.11) | 1.01 (0.79;1.30) | 1.02 (0.94;1.10) | 1.07 (0.79;1.45) |
|  | **Lag**  **0-6** | 1.12 (0.96;1.31) | 0.93 (0.77;1.13) | 1.14 (0.91;1.43) | **0.75 (0.63;0.90)** | 0.97 (0.81;1.18) | 0.98 (0.84;1.14) | 1.09 (0.96;1.25) | 0.94 (0.77;1.14) | 1.02 (0.94;1.11) | 1.01 (0.79;1.30) | 1.02 (0.94;1.10) | 1.07 (0.79;1.45) |
| **Fine Particulate Matter**  **(PM_2.5_)** | **Lag**  **0-1** | 1.11 (0.99;1.24) | 0.97 (0.86;1.09) | 0.92 (0.83;1.02) | 1.07 (0.96;1.18) | 1.08 (0.95;1.22) | 1.02 (0.90;1.15) | 0.98 (0.88;1.09) | 0.97 (0.86;1.09) | 0.98 (0.92;1.05) | **1.17 (1.00;1.37)** | 1.01 (0.95;1.08) | 0.88 (0.73;1.07) |
|  | **Lag**  **0-2** | 1.10 (0.97;1.24) | 0.93 (0.82;1.06) | 0.95 (0.85;1.05) | 1.07 (0.96;1.20) | 1.08 (0.96;1.23) | 1.01 (0.89;1.14) | 0.99 (0.90;1.10) | 0.97 (0.86;1.08) | 0.99 (0.93;1.05) | 1.15 (0.99;1.35) | 1.02 (0.96;1.08) | 0.89 (0.73;1.07) |
|  | **Lag**  **0-3** | 1.01 (0.89;1.16) | 0.91 (0.79;1.05) | 0.98 (0.87;1.09) | 1.09 (0.98;1.23) | 1.10 (0.97;1.24) | 1.01 (0.89;1.14) | 0.99 (0.90;1.10) | 0.97 (0.87;1.09) | 1.00 (0.93;1.06) | 1.13 (0.97;1.32) | 1.02 (0.96;1.08) | 0.89 (0.73;1.07) |
|  | **Lag**  **0-4** | 1.02 (0.88;1.17) | 0.91 (0.78;1.05) | 0.97 (0.87;1.10) | 1.11 (0.98;1.24) | 1.10 (0.97;1.25) | 1.00 (0.88;1.12) | 1.00 (0.90;1.10) | 0.97 (0.87;1.09) | 1.00 (0.94;1.06) | 1.12 (0.96;1.30) | 1.02 (0.96;1.09) | 0.89 (0.74;1.07) |
|  | **Lag**  **0-5** | 1.05 (0.91;1.22) | 0.89 (0.76;1.05) | 0.96 (0.84;1.08) | **1.15 (1.02;1.31)** | 1.11 (0.98;1.26) | 0.99 (0.88;1.11) | 1.00 (0.91;1.10) | 0.97 (0.87;1.09) | 1.00 (0.94;1.06) | 1.12 (0.96;1.30) | 1.02 (0.96;1.09) | 0.89 (0.74;1.07) |
|  | **Lag**  **0-6** | 1.05 (0.90;1.23) | 0.88 (0.75;1.04) | 0.93 (0.82;1.05) | **1.18 (1.04;1.34)** | 1.12 (0.99;1.27) | 0.98 (0.87;1.11) | 1.00 (0.90;1.10) | 0.97 (0.86;1.08) | 1.00 (0.94;1.06) | 1.11 (0.95;1.30) | 1.02 (0.96;1.09) | 0.89 (0.74;1.06) |
| **Maximum Temperature** | **Lag**  **0-1** | **0.81 (0.66;0.99)** | **1.71 (1.42;2.06)** | **1.60 (1.26;2.03)** | 1.01 (0.85;1.19) | 0.86 (0.56;1.34) | 1.20 (0.77;1.87) | 1.16 (0.89;1.51) | 0.83 (0.54;1.28) | 1.03 (0.85;1.26) | 1.22 (0.69;2.16) | 1.06 (0.87;1.29) | 0.90 (0.44;1.81) |
|  | **Lag**  **0-2** | **0.75 (0.61;0.92)** | **1.84 (1.51;2.23)** | **1.60 (1.24;2.06)** | 0.99 (0.83;1.18) | 0.84 (0.55;1.28) | 1.17 (0.76;1.80) | 1.16 (0.89;1.51) | 0.83 (0.55;1.26) | 1.02 (0.84;1.23) | 1.19 (0.68;2.07) | 1.05 (0.87;1.27) | 0.80 (0.41;1.56) |
|  | **Lag**  **0-3** | **0.71 (0.57;0.88)** | **1.84 (1.51;2.25)** | **1.62 (1.24;2.11)** | 0.99 (0.82;1.19) | 0.90 (0.60;1.35) | 1.16 (0.76;1.77) | 1.16 (0.89;1.50) | 0.86 (0.57;1.28) | 1.03 (0.85;1.25) | 1.16 (0.67;2.01) | 1.07 (0.88;1.28) | 0.78 (0.41;1.49) |
|  | **Lag**  **0-4** | **0.68 (0.54;0.85)** | **1.90 (1.54;2.35)** | **1.63 (1.24;2.15)** | 0.97 (0.80;1.17) | 0.93 (0.62;1.39) | 1.19 (0.78;1.80) | 1.16 (0.90;1.50) | 0.86 (0.58;1.28) | 1.04 (0.87;1.26) | 1.15 (0.67;1.97) | 1.07 (0.89;1.29) | 0.78 (0.41;1.49) |
|  | **Lag**  **0-5** | **0.65 (0.51;0.82)** | **2.02 (1.62;2.51)** | **1.64 (1.24;2.18)** | 0.96 (0.78;1.18) | 0.95 (0.64;1.42) | 1.21 (0.80;1.82) | 1.17 (0.91;1.51) | 0.85 (0.57;1.27) | 1.05 (0.87;1.26) | 1.15 (0.67;1.97) | 1.08 (0.90;1.30) | 0.78 (0.41;1.48) |
|  | **Lag**  **0-6** | **0.63 (0.49;0.80)** | **2.10 (1.67;2.63)** | **1.63 (1.22;2.19)** | 1.00 (0.81;1.23) | 0.97 (0.65;1.44) | 1.22 (0.81;1.84) | 1.16 (0.90;1.50) | 0.84 (0.57;1.25) | 1.05 (0.87;1.26) | 1.13 (0.66;1.94) | 1.08 (0.90;1.29) | 0.77 (0.41;1.45) |
| **Atmospheric Pressure** | **Lag**  **0-1** | 1.21 (0.73;2.03) | 1.00 (0.51;1.99) | 0.73 (0.46;1.17) | 1.26 (0.82;1.95) | 1.04 (0.87;1.24) | 1.15 (0.80;1.65) | 0.90 (0.64;1.25) | 1.20 (0.16;9.02) | 1.03 (0.80;1.32) | 1.03 (0.49;2.19) | 1.03 (0.84;1.28) | 0.45 (0.02;8.26) |
|  | **Lag**  **0-2** | 1.07 (0.60;1.91) | 1.34 (0.63;2.86) | 0.71 (0.42;1.18) | 1.14 (0.69;1.90) | 0.98 (0.80;1.19) | 1.26 (0.84;1.90) | 0.85 (0.58;1.24) | 1.80 (0.19;17.4) | 0.97 (0.73;1.29) | 1.24 (0.53;2.91) | 1.01 (0.79;1.29) | 0.49 (0.02;12.3) |
|  | **Lag**  **0-3** | 1.01 (0.53;1.93) | 1.90 (0.82;4.40) | 0.58 (0.33;1.01) | 1.04 (0.58;1.88) | 0.93 (0.75;1.16) | 1.23 (0.78;1.95) | 0.87 (0.57;1.34) | 1.56 (0.12;20.4) | 0.93 (0.67;1.28) | 1.07 (0.42;2.78) | 0.96 (0.73;1.27) | 0.40 (0.01;15.5) |
|  | **Lag**  **0-4** | 0.93 (0.46;1.90) | 2.47 (0.98;6.22) | **0.49 (0.27;0.90)** | 1.20 (0.62;2.29) | 0.92 (0.73;1.17) | 1.24 (0.75;2.05) | 0.92 (0.58;1.47) | 1.55 (0.09;25.4) | 0.94 (0.66;1.33) | 0.87 (0.31;2.44) | 0.95 (0.71;1.29) | 0.37 (0.01;21.3) |
|  | **Lag**  **0-5** | 0.82 (0.39;1.76) | **3.04 (1.13;8.20)** | **0.44 (0.23;0.86)** | 1.10 (0.55;2.23) | 0.91 (0.71;1.17) | 1.17 (0.68;2.01) | 0.91 (0.55;1.51) | 1.83 (0.09;35.5) | 0.90 (0.62;1.31) | 0.74 (0.25;2.20) | 0.90 (0.65;1.24) | 0.62 (0.01;48.1) |
|  | **Lag**  **0-6** | 0.60 (0.26;1.35) | **3.57 (1.23;10.4)** | **0.37 (0.18;0.74)** | 1.36 (0.62;2.95) | 0.89 (0.68;1.18) | 1.16 (0.65;2.08) | 0.92 (0.54;1.57) | 1.57 (0.07;35.3) | 0.89 (0.60;1.34) | 0.72 (0.22;2.31) | 0.90 (0.64;1.28) | 0.42 (0.00;42.5) |

*Air pollution models (PM_2.5_, NO_2_, O_3_) were adjusted for maximum temperature, precipitation (mm/day), and shortwave solar radiation (W/m²). Maximum temperature models were adjusted for PM_2.5_, precipitation, and solar radiation. Atmospheric pressure models were adjusted for PM_2.5_, maximum temperature, precipitation, and solar radiation. Results are expressed as adjusted odds ratios (ORs) calculated per interquartile range (IQR) increase of each exposure.*

**Table S6:** Adjusted odds ratios (ORs) and 95% confidence intervals (CIs) for exposures across *cumulative lag days* (0–1 to 0–6) from *opioid overdose*-stratified models, presented by season, region, rural/urban classification, and elevation. Estimates with CIs that do not cross 1 are highlighted in bold.

|  |  |  | | | | **Opioid Overdose** | | | |  | | | |
| --- | --- | --- | --- | --- | --- | --- | --- | --- | --- | --- | --- | --- | --- |
|  |  | **Season** | | | | **Region** | | | | **Urbanicity** | | **Altitude** | |
|  |  | **Spring** | **Summer** | **Fall** | **Winter** | **North Central** | **Northeast** | **South** | **West** | **Metro** | **Nonmetro** | **< 500 m** | **>= 500 m** |
| **Nitrogen Dioxide (NO_2_)** | **Lag**  **0-1** | 0.97 (0.80;1.17) | 0.88 (0.72;1.07) | 0.94 (0.77;1.14) | 1.13 (0.96;1.34) | 1.00 (0.85;1.18) | 1.04 (0.88;1.24) | 1.04 (0.91;1.20) | 1.01 (0.79;1.29) | 1.01 (0.93;1.10) | 1.23 (0.98;1.55) | 1.03 (0.95;1.11) | 0.87 (0.59;1.28) |
|  | **Lag**  **0-2** | 1.06 (0.85;1.31) | 0.90 (0.72;1.14) | 0.98 (0.79;1.22) | 1.11 (0.92;1.34) | 1.01 (0.86;1.19) | 1.03 (0.87;1.23) | 1.05 (0.91;1.20) | 1.00 (0.79;1.28) | 1.01 (0.93;1.10) | 1.24 (0.98;1.56) | 1.03 (0.95;1.11) | 0.88 (0.60;1.29) |
|  | **Lag**  **0-3** | 0.97 (0.77;1.23) | 0.88 (0.68;1.14) | 1.03 (0.81;1.31) | 1.15 (0.94;1.42) | 1.02 (0.87;1.21) | 1.03 (0.87;1.22) | 1.05 (0.91;1.20) | 1.01 (0.79;1.29) | 1.01 (0.93;1.10) | 1.23 (0.98;1.55) | 1.03 (0.95;1.12) | 0.87 (0.60;1.28) |
|  | **Lag**  **0-4** | 0.98 (0.75;1.29) | 0.86 (0.65;1.13) | 1.07 (0.82;1.39) | 1.21 (0.96;1.52) | 1.03 (0.87;1.21) | 1.03 (0.87;1.23) | 1.05 (0.92;1.20) | 1.00 (0.78;1.27) | 1.01 (0.93;1.10) | 1.22 (0.97;1.53) | 1.03 (0.95;1.12) | 0.88 (0.60;1.29) |
|  | **Lag**  **0-5** | 1.00 (0.75;1.33) | 0.82 (0.61;1.10) | 1.09 (0.83;1.44) | 1.23 (0.95;1.58) | 1.04 (0.88;1.22) | 1.03 (0.86;1.22) | 1.06 (0.92;1.21) | 0.98 (0.77;1.26) | 1.02 (0.93;1.10) | 1.21 (0.96;1.52) | 1.03 (0.95;1.12) | 0.88 (0.60;1.29) |
|  | **Lag**  **0-6** | 1.00 (0.74;1.37) | 0.78 (0.56;1.07) | 1.09 (0.81;1.46) | **1.31 (1.01;1.70)** | 1.04 (0.88;1.23) | 1.03 (0.86;1.22) | 1.06 (0.92;1.22) | 0.97 (0.76;1.24) | 1.02 (0.94;1.10) | 1.21 (0.96;1.52) | 1.03 (0.95;1.12) | 0.87 (0.59;1.28) |
| **Ozone**  **(O_3_)** | **Lag**  **0-1** | 0.97 (0.81;1.15) | 1.01 (0.83;1.21) | 1.08 (0.90;1.30) | 0.86 (0.73;1.01) | 0.99 (0.78;1.25) | 0.92 (0.76;1.11) | 1.09 (0.91;1.30) | 1.13 (0.83;1.54) | 1.00 (0.90;1.12) | 1.18 (0.82;1.69) | 1.01 (0.91;1.12) | 1.26 (0.81;1.96) |
|  | **Lag**  **0-2** | 0.98 (0.82;1.18) | 1.04 (0.85;1.26) | 1.05 (0.85;1.29) | **0.77 (0.64;0.93)** | 0.99 (0.79;1.24) | 0.92 (0.77;1.11) | 1.09 (0.91;1.29) | 1.10 (0.81;1.49) | 1.01 (0.90;1.12) | 1.19 (0.83;1.70) | 1.01 (0.91;1.12) | 1.23 (0.80;1.90) |
|  | **Lag**  **0-3** | 1.05 (0.87;1.26) | 1.05 (0.84;1.29) | 1.10 (0.87;1.39) | **0.77 (0.63;0.93)** | 1.00 (0.80;1.25) | 0.95 (0.80;1.14) | 1.08 (0.91;1.29) | 1.08 (0.80;1.45) | 1.01 (0.91;1.13) | 1.16 (0.81;1.66) | 1.02 (0.92;1.13) | 1.22 (0.80;1.88) |
|  | **Lag**  **0-4** | 1.06 (0.87;1.29) | 1.05 (0.84;1.32) | 1.18 (0.92;1.52) | **0.72 (0.58;0.89)** | 1.00 (0.80;1.25) | 0.96 (0.80;1.14) | 1.08 (0.91;1.29) | 1.08 (0.80;1.45) | 1.02 (0.92;1.13) | 1.17 (0.82;1.67) | 1.02 (0.92;1.13) | 1.24 (0.81;1.90) |
|  | **Lag**  **0-5** | 1.08 (0.88;1.32) | 1.01 (0.79;1.28) | 1.22 (0.94;1.60) | **0.70 (0.57;0.88)** | 1.00 (0.80;1.25) | 0.96 (0.81;1.14) | 1.08 (0.91;1.29) | 1.08 (0.80;1.46) | 1.02 (0.92;1.13) | 1.16 (0.81;1.66) | 1.02 (0.92;1.13) | 1.27 (0.83;1.95) |
|  | **Lag**  **0-6** | 1.08 (0.88;1.33) | 0.99 (0.78;1.27) | 1.19 (0.89;1.57) | **0.71 (0.57;0.89)** | 1.00 (0.80;1.25) | 0.96 (0.81;1.14) | 1.08 (0.91;1.28) | 1.09 (0.81;1.47) | 1.02 (0.92;1.13) | 1.17 (0.82;1.67) | 1.01 (0.92;1.12) | 1.28 (0.83;1.97) |
| **Fine Particulate Matter**  **(PM_2.5_)** | **Lag**  **0-1** | 1.03 (0.88;1.20) | 0.92 (0.79;1.07) | 0.89 (0.78;1.01) | 1.09 (0.96;1.24) | 1.04 (0.89;1.22) | 1.06 (0.92;1.22) | 0.92 (0.80;1.05) | 0.99 (0.82;1.18) | 0.98 (0.90;1.06) | 1.24 (0.96;1.59) | 1.01 (0.93;1.09) | 0.78 (0.58;1.05) |
|  | **Lag**  **0-2** | 1.06 (0.90;1.25) | 0.92 (0.78;1.08) | 0.91 (0.79;1.05) | 1.09 (0.95;1.23) | 1.06 (0.91;1.23) | 1.04 (0.90;1.19) | 0.93 (0.82;1.06) | 0.98 (0.83;1.17) | 0.98 (0.91;1.06) | 1.22 (0.96;1.55) | 1.01 (0.94;1.09) | 0.82 (0.63;1.09) |
|  | **Lag**  **0-3** | 0.99 (0.84;1.18) | 0.95 (0.80;1.14) | 0.94 (0.81;1.09) | 1.11 (0.98;1.27) | 1.08 (0.92;1.26) | 1.03 (0.90;1.19) | 0.93 (0.82;1.06) | 0.99 (0.83;1.18) | 0.99 (0.91;1.07) | 1.17 (0.92;1.49) | 1.01 (0.94;1.09) | 0.81 (0.62;1.07) |
|  | **Lag**  **0-4** | 1.03 (0.86;1.22) | 0.95 (0.79;1.15) | 0.95 (0.82;1.11) | 1.13 (0.99;1.30) | 1.08 (0.93;1.26) | 1.03 (0.89;1.18) | 0.94 (0.83;1.07) | 0.99 (0.83;1.18) | 0.99 (0.92;1.07) | 1.13 (0.89;1.44) | 1.01 (0.94;1.09) | 0.83 (0.63;1.08) |
|  | **Lag**  **0-5** | 1.04 (0.86;1.26) | 0.95 (0.78;1.16) | 0.95 (0.81;1.11) | **1.20 (1.04;1.38)** | 1.09 (0.94;1.27) | 1.01 (0.88;1.16) | 0.95 (0.84;1.07) | 0.99 (0.83;1.18) | 0.99 (0.92;1.07) | 1.13 (0.89;1.43) | 1.01 (0.94;1.09) | 0.83 (0.64;1.09) |
|  | **Lag**  **0-6** | 1.00 (0.82;1.23) | 0.96 (0.77;1.18) | 0.94 (0.80;1.11) | **1.24 (1.07;1.43)** | 1.10 (0.95;1.29) | 1.01 (0.88;1.16) | 0.95 (0.84;1.08) | 0.98 (0.82;1.17) | 0.99 (0.92;1.07) | 1.13 (0.89;1.43) | 1.02 (0.94;1.10) | 0.84 (0.65;1.10) |
| **Maximum Temperature** | **Lag**  **0-1** | 0.83 (0.64;1.07) | **1.59 (1.28;1.99)** | **1.88 (1.39;2.54)** | 0.94 (0.77;1.15) | 0.89 (0.52;1.51) | 1.09 (0.65;1.82) | 1.38 (0.97;1.95) | 1.01 (0.54;1.90) | 1.07 (0.83;1.38) | 1.83 (0.81;4.12) | 1.11 (0.87;1.42) | 1.01 (0.39;2.63) |
|  | **Lag**  **0-2** | 0.78 (0.60;1.01) | **1.65 (1.31;2.09)** | **1.88 (1.37;2.59)** | 0.92 (0.75;1.13) | 0.89 (0.54;1.48) | 1.05 (0.64;1.72) | 1.35 (0.96;1.91) | 0.98 (0.54;1.78) | 1.04 (0.82;1.33) | 1.82 (0.82;4.01) | 1.09 (0.86;1.39) | 0.90 (0.37;2.21) |
|  | **Lag**  **0-3** | **0.76 (0.58;0.99)** | **1.57 (1.24;1.99)** | **1.92 (1.38;2.69)** | 0.92 (0.75;1.14) | 0.93 (0.57;1.53) | 1.06 (0.66;1.72) | 1.33 (0.95;1.87) | 0.95 (0.53;1.71) | 1.06 (0.83;1.34) | 1.74 (0.80;3.78) | 1.11 (0.87;1.40) | 0.84 (0.35;2.01) |
|  | **Lag**  **0-4** | **0.75 (0.56;0.99)** | **1.67 (1.29;2.14)** | **1.96 (1.38;2.77)** | 0.92 (0.74;1.14) | 0.93 (0.57;1.51) | 1.10 (0.68;1.77) | 1.34 (0.96;1.87) | 0.91 (0.51;1.63) | 1.07 (0.85;1.35) | 1.62 (0.75;3.49) | 1.11 (0.88;1.41) | 0.86 (0.36;2.02) |
|  | **Lag**  **0-5** | **0.72 (0.53;0.97)** | **1.81 (1.39;2.36)** | **1.97 (1.38;2.82)** | 0.93 (0.74;1.18) | 0.94 (0.58;1.52) | 1.12 (0.70;1.79) | 1.36 (0.97;1.89) | 0.90 (0.50;1.60) | 1.07 (0.85;1.36) | 1.61 (0.75;3.47) | 1.12 (0.89;1.41) | 0.84 (0.36;1.97) |
|  | **Lag**  **0-6** | **0.69 (0.51;0.95)** | **1.94 (1.47;2.56)** | **1.99 (1.37;2.89)** | 0.98 (0.78;1.24) | 0.95 (0.58;1.54) | 1.13 (0.71;1.81) | 1.36 (0.98;1.90) | 0.88 (0.50;1.58) | 1.08 (0.85;1.36) | 1.65 (0.76;3.55) | 1.12 (0.89;1.42) | 0.85 (0.37;1.99) |
| **Atmospheric Pressure** | **Lag**  **0-1** | 1.23 (0.70;2.15) | 1.11 (0.51;2.42) | 0.84 (0.51;1.39) | 1.10 (0.68;1.77) | 0.95 (0.78;1.16) | 1.28 (0.87;1.87) | 1.03 (0.68;1.56) | 0.32 (0.01;10.15) | 1.06 (0.81;1.38) | 1.01 (0.39;2.61) | 1.03 (0.81;1.32) | 1.91 (0.03;107) |
|  | **Lag**  **0-2** | 1.01 (0.54;1.90) | 1.43 (0.60;3.39) | 0.78 (0.44;1.37) | 1.02 (0.59;1.74) | 0.87 (0.70;1.09) | 1.42 (0.91;2.21) | 0.90 (0.56;1.44) | 1.37 (0.03;65.22) | 0.97 (0.71;1.31) | 1.44 (0.49;4.21) | 0.98 (0.74;1.29) | 2.28 (0.03;186) |
|  | **Lag**  **0-3** | 1.05 (0.52;2.12) | 1.77 (0.68;4.62) | 0.59 (0.32;1.09) | 0.93 (0.50;1.75) | 0.83 (0.66;1.06) | 1.38 (0.84;2.25) | 0.86 (0.50;1.45) | 2.04 (0.02;168.4) | 0.90 (0.64;1.27) | 1.12 (0.35;3.63) | 0.92 (0.67;1.26) | 0.75 (0.01;110) |
|  | **Lag**  **0-4** | 0.97 (0.45;2.10) | 1.91 (0.67;5.44) | **0.49 (0.25;0.97)** | 0.97 (0.49;1.93) | 0.81 (0.63;1.05) | 1.33 (0.78;2.27) | 0.82 (0.46;1.46) | 1.86 (0.02;212.2) | 0.84 (0.58;1.23) | 0.81 (0.24;2.80) | 0.85 (0.61;1.20) | 0.56 (0.00;135) |
|  | **Lag**  **0-5** | 0.84 (0.37;1.92) | 1.98 (0.65;6.07) | **0.44 (0.21;0.91)** | 0.89 (0.42;1.89) | 0.79 (0.60;1.05) | 1.17 (0.66;2.06) | 0.76 (0.41;1.42) | 2.76 (0.02;432.4) | 0.77 (0.51;1.16) | 0.64 (0.17;2.32) | 0.77 (0.53;1.11) | 0.65 (0.00;259) |
|  | **Lag**  **0-6** | 0.63 (0.26;1.54) | 2.10 (0.63;7.01) | **0.34 (0.16;0.74)** | 1.11 (0.48;2.55) | 0.77 (0.57;1.03) | 1.12 (0.60;2.07) | 0.77 (0.39;1.51) | 2.21 (0.01;447.1) | 0.74 (0.48;1.15) | 0.58 (0.15;2.29) | 0.75 (0.50;1.11) | 0.46 (0.00;250) |

*Air pollution models (PM_2.5_, NO_2_, O_3_) were adjusted for maximum temperature, precipitation (mm/day), and shortwave solar radiation (W/m²). Maximum temperature models were adjusted for PM_2.5_, precipitation, and solar radiation. Atmospheric pressure models were adjusted for PM_2.5_, maximum temperature, precipitation, and solar radiation. Results are expressed as adjusted odds ratios (ORs) calculated per interquartile range (IQR) increase of each exposure.*

**Table S7:** Adjusted odds ratios (ORs) and 95% confidence intervals (CIs) for exposures across *single lag days* (0 to 6) from *suicide*-stratified models, presented by season, region, rural/urban classification, and elevation. Estimates with CIs that do not cross 1 are highlighted in bold.

|  |  |  | | | | **Suicide** | | | |  | | | |
| --- | --- | --- | --- | --- | --- | --- | --- | --- | --- | --- | --- | --- | --- |
|  |  | **Season** | | | | **Region** | | | | **Urbanicity** | | **Altitude** | |
|  |  | **Spring** | **Summer** | **Fall** | **Winter** | **North Central** | **Northeast** | **South** | **West** | **Metro** | **Nonmetro** | **< 500 m** | **>= 500 m** |
| **Nitrogen Dioxide**  **(NO_2_)** | **Lag 0** | 0.99 (0.90;1.09) | 1.09 (0.97;1.22) | **0.91 (0.82;1.00)** | **1.11 (1.01;1.21)** | **1.11 (1.00;1.24)** | 0.96 (0.82;1.13) | 1.00 (0.93;1.08) | 0.94 (0.81;1.08) | 1.01 (0.95;1.07) | 1.04 (0.92;1.18) | 1.02 (0.97;1.08) | 0.94 (0.81;1.10) |
|  | **Lag 1** | 0.97 (0.88;1.07) | 0.97 (0.87;1.09) | **0.88 (0.80;0.98)** | **1.16 (1.06;1.27)** | **1.11 (1.00;1.24)** | 0.97 (0.83;1.14) | 1.02 (0.94;1.10) | 0.95 (0.83;1.09) | 1.02 (0.97;1.08) | 1.04 (0.92;1.17) | 1.04 (0.98;1.09) | 0.93 (0.80;1.08) |
|  | **Lag 2** | 1.01 (0.92;1.12) | 0.92 (0.82;1.03) | 0.98 (0.88;1.09) | **1.16 (1.06;1.27)** | 1.11 (0.99;1.23) | 0.95 (0.81;1.11) | 1.00 (0.93;1.08) | 0.96 (0.84;1.10) | 1.01 (0.96;1.07) | 1.04 (0.92;1.17) | 1.02 (0.97;1.08) | 0.93 (0.80;1.08) |
|  | **Lag 3** | 0.97 (0.88;1.06) | 0.93 (0.83;1.04) | 1.04 (0.94;1.15) | 1.09 (0.99;1.19) | 1.10 (0.98;1.22) | 0.95 (0.81;1.11) | 1.00 (0.93;1.08) | 0.97 (0.85;1.11) | 1.01 (0.96;1.07) | 1.03 (0.92;1.17) | 1.02 (0.97;1.08) | 0.94 (0.80;1.09) |
|  | **Lag 4** | 0.97 (0.88;1.06) | 0.96 (0.86;1.07) | 0.98 (0.88;1.08) | 0.97 (0.88;1.07) | 1.10 (0.99;1.22) | 0.96 (0.82;1.12) | 1.00 (0.93;1.07) | 0.96 (0.84;1.11) | 1.01 (0.96;1.07) | 1.03 (0.92;1.17) | 1.02 (0.97;1.08) | 0.94 (0.80;1.09) |
|  | **Lag 5** | 1.01 (0.91;1.11) | **0.88 (0.79;0.99)** | 0.97 (0.88;1.08) | 1.05 (0.95;1.15) | 1.10 (0.99;1.22) | 0.96 (0.82;1.12) | 1.00 (0.93;1.07) | 0.96 (0.84;1.10) | 1.01 (0.96;1.07) | 1.04 (0.92;1.17) | 1.02 (0.97;1.08) | 0.93 (0.80;1.08) |
|  | **Lag 6** | 0.93 (0.84;1.03) | **0.84 (0.75;0.94)** | 0.98 (0.88;1.09) | **1.11 (1.01;1.21)** | 1.10 (0.98;1.22) | 0.97 (0.83;1.14) | 1.00 (0.93;1.08) | 0.96 (0.84;1.10) | 1.01 (0.96;1.07) | 1.04 (0.92;1.18) | 1.02 (0.97;1.08) | 0.93 (0.80;1.08) |
| **Ozone**  **(O_3_)** | **Lag 0** | 0.99 (0.90;1.10) | 0.98 (0.88;1.09) | 1.03 (0.91;1.15) | 0.89 (0.81;0.99) | 0.99 (0.84;1.17) | 0.90 (0.73;1.09) | 1.01 (0.91;1.13) | 0.90 (0.77;1.05) | 0.97 (0.89;1.05) | 0.96 (0.81;1.12) | 0.97 (0.90;1.05) | 0.94 (0.77;1.15) |
|  | **Lag 1** | 1.05 (0.95;1.16) | 0.95 (0.85;1.06) | **1.12 (1.00;1.26)** | 0.87 (0.78;0.96) | 1.00 (0.86;1.17) | 0.96 (0.81;1.14) | 1.02 (0.92;1.13) | 0.93 (0.81;1.08) | 0.99 (0.92;1.07) | 0.99 (0.85;1.15) | 0.99 (0.92;1.06) | 0.98 (0.81;1.18) |
|  | **Lag 2** | 0.98 (0.89;1.09) | 0.90 (0.81;1.01) | **1.15 (1.02;1.29)** | 0.88 (0.80;0.97) | 0.99 (0.85;1.15) | 0.93 (0.78;1.10) | 1.02 (0.92;1.13) | 0.95 (0.82;1.09) | 0.98 (0.91;1.06) | 0.99 (0.85;1.15) | 0.99 (0.92;1.06) | 0.99 (0.82;1.19) |
|  | **Lag 3** | 1.02 (0.93;1.13) | 0.83 (0.75;0.93) | 1.06 (0.94;1.19) | 0.85 (0.77;0.94) | 0.98 (0.84;1.14) | 0.94 (0.80;1.11) | 1.01 (0.91;1.12) | 0.95 (0.83;1.10) | 0.98 (0.91;1.06) | 0.98 (0.84;1.14) | 0.98 (0.91;1.05) | 0.99 (0.82;1.20) |
|  | **Lag 4** | **1.11 (1.01;1.22)** | 0.91 (0.82;1.02) | 1.11 (0.99;1.24) | 0.88 (0.80;0.97) | 0.98 (0.84;1.14) | 0.94 (0.80;1.12) | 1.02 (0.92;1.13) | 0.95 (0.82;1.10) | 0.98 (0.91;1.06) | 0.98 (0.84;1.14) | 0.98 (0.92;1.06) | 0.99 (0.82;1.20) |
|  | **Lag 5** | 1.07 (0.97;1.17) | 0.87 (0.77;0.97) | 0.98 (0.87;1.10) | 0.90 (0.81;0.99) | 0.98 (0.84;1.14) | 0.95 (0.81;1.13) | 1.01 (0.91;1.12) | 0.94 (0.82;1.09) | 0.98 (0.91;1.06) | 0.99 (0.85;1.15) | 0.98 (0.92;1.06) | 0.98 (0.81;1.18) |
|  | **Lag 6** | 1.04 (0.95;1.14) | 0.89 (0.80;1.00) | 0.96 (0.85;1.08) | 0.87 (0.79;0.96) | 0.97 (0.84;1.13) | 0.97 (0.82;1.14) | 1.02 (0.92;1.13) | 0.95 (0.82;1.10) | 0.99 (0.91;1.06) | 0.98 (0.84;1.14) | 0.99 (0.92;1.06) | 0.98 (0.81;1.19) |
| **Fine Particulate Matter**  **(PM_2.5_)** | **Lag 0** | 1.03 (0.94;1.13) | 0.97 (0.89;1.06) | 0.93 (0.85;1.01) | **1.10 (1.01;1.19)** | 1.03 (0.92;1.14) | 0.95 (0.82;1.09) | 0.99 (0.92;1.07) | 0.98 (0.89;1.07) | 1.02 (0.97;1.08) | **0.88 (0.78;0.99)** | 1.00 (0.95;1.06) | 0.92 (0.81;1.05) |
|  | **Lag 1** | **1.09 (1.00;1.19)** | 0.97 (0.89;1.06) | **0.92 (0.85;1.00)** | **1.14 (1.04;1.24)** | 1.05 (0.95;1.17) | 0.97 (0.84;1.11) | 1.02 (0.94;1.10) | 0.99 (0.91;1.08) | 1.04 (0.98;1.10) | 0.90 (0.80;1.01) | 1.02 (0.97;1.08) | 0.93 (0.82;1.05) |
|  | **Lag 2** | 0.98 (0.89;1.07) | 0.96 (0.89;1.05) | 1.03 (0.96;1.11) | **1.16 (1.07;1.27)** | 1.03 (0.93;1.14) | 0.94 (0.83;1.08) | 1.00 (0.93;1.07) | 1.00 (0.92;1.08) | 1.02 (0.97;1.08) | 0.90 (0.81;1.01) | 1.01 (0.96;1.06) | 0.95 (0.84;1.07) |
|  | **Lag 3** | 1.04 (0.95;1.14) | 0.92 (0.85;1.01) | 1.02 (0.94;1.10) | 0.98 (0.90;1.07) | 1.02 (0.92;1.13) | 0.93 (0.82;1.06) | 0.99 (0.92;1.07) | 1.00 (0.92;1.09) | 1.02 (0.97;1.07) | 0.90 (0.81;1.01) | 1.00 (0.95;1.05) | 0.95 (0.84;1.07) |
|  | **Lag 4** | **1.10 (1.01;1.20)** | **0.91 (0.84;1.00)** | 0.95 (0.88;1.03) | 0.97 (0.88;1.06) | 1.03 (0.93;1.14) | 0.94 (0.82;1.07) | 0.99 (0.92;1.06) | 1.00 (0.92;1.09) | 1.02 (0.97;1.07) | 0.90 (0.81;1.00) | 1.00 (0.96;1.05) | 0.95 (0.84;1.07) |
|  | **Lag 5** | 1.05 (0.96;1.15) | **0.91 (0.83;1.00)** | 0.95 (0.87;1.02) | 1.05 (0.96;1.15) | 1.02 (0.92;1.13) | 0.95 (0.84;1.08) | 0.99 (0.93;1.07) | 1.00 (0.92;1.09) | 1.02 (0.97;1.08) | 0.90 (0.81;1.01) | 1.01 (0.96;1.06) | 0.94 (0.83;1.06) |
|  | **Lag 6** | 1.07 (0.97;1.17) | 0.94 (0.86;1.03) | 0.95 (0.87;1.02) | 0.97 (0.89;1.06) | 1.02 (0.92;1.13) | 0.96 (0.84;1.09) | 1.00 (0.93;1.07) | 1.00 (0.92;1.09) | 1.02 (0.97;1.08) | 0.90 (0.81;1.01) | 1.01 (0.96;1.06) | 0.95 (0.84;1.07) |
| **Maximum Temperature** | **Lag 0** | **1.23 (1.04;1.47)** | **1.46 (1.27;1.68)** | 1.01 (0.83;1.24) | 0.99 (0.85;1.16) | 0.87 (0.55;1.36) | **1.87 (1.02;3.41)** | 1.02 (0.83;1.26) | **1.64 (1.06;2.55)** | **1.21 (1.00;1.47)** | 0.74 (0.50;1.11) | 1.05 (0.87;1.26) | 1.55 (0.91;2.64) |
|  | **Lag 1** | 1.12 (0.95;1.33) | **1.49 (1.30;1.71)** | 1.18 (0.96;1.44) | 0.95 (0.81;1.10) | 0.87 (0.61;1.24) | 1.30 (0.79;2.12) | 1.02 (0.85;1.23) | **1.54 (1.10;2.16)** | 1.15 (0.98;1.36) | 0.84 (0.61;1.17) | 1.05 (0.89;1.22) | 1.28 (0.86;1.92) |
|  | **Lag 2** | 1.10 (0.92;1.31) | **1.45 (1.26;1.67)** | 1.19 (0.98;1.45) | **0.84 (0.73;0.98)** | 0.87 (0.61;1.23) | 1.24 (0.76;2.01) | 1.02 (0.85;1.23) | **1.52 (1.09;2.11)** | 1.15 (0.98;1.35) | 0.84 (0.61;1.17) | 1.05 (0.90;1.22) | 1.30 (0.87;1.93) |
|  | **Lag 3** | 1.00 (0.84;1.19) | **1.53 (1.33;1.77)** | 1.11 (0.91;1.35) | **0.85 (0.73;0.99)** | 0.85 (0.60;1.21) | 1.26 (0.77;2.03) | 1.03 (0.85;1.24) | **1.58 (1.13;2.20)** | 1.15 (0.98;1.35) | 0.85 (0.61;1.18) | 1.05 (0.90;1.22) | 1.29 (0.87;1.92) |
|  | **Lag 4** | 0.91 (0.76;1.08) | **1.48 (1.28;1.71)** | 1.11 (0.91;1.35) | **0.86 (0.74;0.99)** | 0.87 (0.61;1.23) | 1.25 (0.77;2.02) | 1.02 (0.85;1.23) | **1.54 (1.11;2.15)** | 1.15 (0.98;1.35) | 0.84 (0.61;1.17) | 1.04 (0.90;1.22) | 1.29 (0.87;1.92) |
|  | **Lag 5** | 0.93 (0.78;1.11) | **1.52 (1.31;1.76)** | 1.05 (0.87;1.28) | 0.92 (0.79;1.07) | 0.84 (0.59;1.20) | 1.25 (0.77;2.01) | 1.02 (0.85;1.22) | **1.53 (1.10;2.13)** | 1.14 (0.97;1.34) | 0.84 (0.61;1.16) | 1.04 (0.89;1.21) | 1.32 (0.89;1.95) |
|  | **Lag 6** | 0.98 (0.82;1.16) | **1.34 (1.16;1.54)** | 1.05 (0.86;1.28) | 0.96 (0.83;1.12) | 0.86 (0.60;1.22) | 1.25 (0.77;2.02) | 1.02 (0.85;1.23) | **1.53 (1.10;2.14)** | 1.15 (0.98;1.35) | 0.83 (0.60;1.15) | 1.04 (0.89;1.21) | 1.29 (0.87;1.90) |
| **Atmospheric Pressure** | **Lag 0** | 0.99 (0.57;1.72) | 0.55 (0.27;1.11) | **0.52 (0.34;0.81)** | 1.22 (0.76;1.97) | 0.94 (0.80;1.11) | 1.05 (0.69;1.62) | 0.84 (0.66;1.07) | 0.54 (0.11;2.66) | 0.90 (0.68;1.20) | **0.62 (0.38;1.00)** | 0.88 (0.73;1.06) | 0.57 (0.11;2.95) |
|  | **Lag 1** | 0.83 (0.48;1.43) | 1.02 (0.51;2.07) | 0.89 (0.58;1.37) | **2.29 (1.42;3.68)** | 1.16 (0.99;1.37) | 1.38 (0.91;2.10) | 1.09 (0.85;1.39) | 0.78 (0.16;3.75) | **1.34 (1.01;1.77)** | 0.90 (0.56;1.46) | **1.22 (1.01;1.46)** | 0.49 (0.10;2.45) |
|  | **Lag 2** | 0.84 (0.48;1.47) | 1.30 (0.64;2.66) | 1.44 (0.93;2.22) | 1.58 (0.99;2.54) | 1.06 (0.90;1.25) | 1.48 (0.97;2.24) | 1.22 (0.95;1.56) | 1.42 (0.30;6.79) | 1.26 (0.95;1.66) | 1.35 (0.82;2.20) | 1.19 (0.99;1.43) | 1.92 (0.38;9.74) |
|  | **Lag 3** | 0.87 (0.50;1.53) | 1.13 (0.57;2.26) | 0.99 (0.65;1.51) | 1.13 (0.71;1.81) | 1.02 (0.87;1.20) | 0.86 (0.57;1.30) | 1.11 (0.86;1.41) | 0.81 (0.16;3.99) | 1.02 (0.77;1.34) | 1.00 (0.61;1.62) | 0.99 (0.83;1.18) | 1.37 (0.26;7.13) |
|  | **Lag 4** | 0.59 (0.34;1.02) | 1.00 (0.50;2.02) | 0.80 (0.51;1.25) | 1.17 (0.73;1.86) | 0.98 (0.84;1.15) | 0.68 (0.45;1.04) | 1.06 (0.83;1.35) | 0.70 (0.15;3.34) | 0.94 (0.71;1.24) | 0.74 (0.46;1.19) | 0.93 (0.77;1.11) | 0.61 (0.12;3.07) |
|  | **Lag 5** | **0.46 (0.27;0.77)** | 1.88 (0.92;3.81) | 0.85 (0.54;1.34) | 1.19 (0.76;1.86) | 0.97 (0.83;1.14) | 0.66 (0.43;1.01) | 1.12 (0.88;1.43) | 1.24 (0.26;5.92) | 0.97 (0.74;1.29) | 0.91 (0.57;1.45) | 0.99 (0.82;1.18) | 0.95 (0.19;4.64) |
|  | **Lag 6** | **0.50 (0.29;0.85)** | **2.09 (1.01;4.31)** | 1.07 (0.68;1.69) | **2.18 (1.39;3.43)** | 1.01 (0.86;1.19) | 0.87 (0.57;1.34) | **1.31 (1.02;1.67)** | 1.51 (0.32;7.04) | 1.27 (0.96;1.67) | 1.03 (0.64;1.66) | 1.17 (0.98;1.41) | 1.37 (0.28;6.66) |

*Air pollution models (PM_2.5_, NO_2_, O_3_) were adjusted for maximum temperature, precipitation (mm/day), and shortwave solar radiation (W/m²). Maximum temperature models were adjusted for PM_2.5_, precipitation, and solar radiation. Atmospheric pressure models were adjusted for PM_2.5_, maximum temperature, precipitation, and solar radiation. Results are expressed as adjusted odds ratios (ORs) calculated per interquartile range (IQR) increase of each exposure.*

**Table S8:** Adjusted odds ratios (ORs) and 95% confidence intervals (CIs) for exposures across *single lag days* (0 to 6) from *overdose*-stratified models, presented by season, region, rural/urban classification, and elevation. Estimates with CIs that do not cross 1 are highlighted in bold.

|  |  |  | | | | **Overdose** | | | |  | | | |
| --- | --- | --- | --- | --- | --- | --- | --- | --- | --- | --- | --- | --- | --- |
|  |  | **Season** | | | | **Region** | | | | **Urbanicity** | | **Altitude** | |
|  |  | **Spring** | **Summer** | **Fall** | **Winter** | **North Central** | **Northeast** | **South** | **West** | **Metro** | **Nonmetro** | **< 500 m** | **>= 500 m** |
| **Nitrogen Dioxide**  **(NO_2_)** | **Lag 0** | 1.04 (0.92;1.18) | 0.88 (0.76;1.01) | 0.99 (0.87;1.12) | 1.06 (0.94;1.20) | 1.07 (0.94;1.23) | 1.02 (0.88;1.20) | 1.05 (0.94;1.16) | 0.93 (0.78;1.10) | 1.01 (0.94;1.08) | **1.24 (1.04;1.49)** | 1.02 (0.96;1.09) | 0.95 (0.74;1.21) |
|  | **Lag 1** | 0.95 (0.84;1.08) | 0.93 (0.81;1.06) | 1.00 (0.88;1.13) | 1.06 (0.95;1.19) | 1.10 (0.96;1.25) | 1.01 (0.87;1.18) | 1.07 (0.96;1.19) | 0.94 (0.80;1.11) | 1.02 (0.96;1.09) | **1.27 (1.07;1.51)** | 1.04 (0.97;1.11) | 0.96 (0.75;1.22) |
|  | **Lag 2** | 1.03 (0.92;1.16) | 0.96 (0.83;1.11) | 1.07 (0.94;1.22) | 1.07 (0.95;1.20) | 1.09 (0.96;1.25) | 1.01 (0.87;1.18) | 1.06 (0.96;1.18) | 0.93 (0.78;1.09) | 1.02 (0.96;1.09) | **1.24 (1.04;1.48)** | 1.04 (0.97;1.11) | 0.96 (0.75;1.22) |
|  | **Lag 3** | 0.90 (0.80;1.01) | 0.89 (0.77;1.02) | 1.05 (0.92;1.18) | 1.10 (0.97;1.24) | 1.09 (0.96;1.25) | 1.02 (0.87;1.19) | 1.06 (0.96;1.18) | 0.93 (0.78;1.10) | 1.02 (0.96;1.09) | **1.25 (1.05;1.49)** | 1.04 (0.97;1.11) | 0.96 (0.75;1.22) |
|  | **Lag 4** | 1.02 (0.91;1.15) | **0.86 (0.75;0.99)** | 1.00 (0.88;1.13) | **1.14 (1.01;1.29)** | 1.10 (0.96;1.25) | 1.02 (0.87;1.19) | 1.06 (0.96;1.18) | 0.92 (0.78;1.09) | 1.02 (0.95;1.09) | **1.24 (1.04;1.48)** | 1.04 (0.97;1.10) | 0.95 (0.75;1.21) |
|  | **Lag 5** | 1.01 (0.90;1.13) | 0.93 (0.82;1.07) | 0.94 (0.83;1.07) | 1.09 (0.97;1.23) | 1.10 (0.97;1.26) | 1.02 (0.88;1.19) | 1.06 (0.95;1.18) | 0.92 (0.78;1.09) | 1.02 (0.95;1.09) | **1.24 (1.04;1.48)** | 1.04 (0.97;1.11) | 0.95 (0.75;1.21) |
|  | **Lag 6** | 0.99 (0.88;1.12) | 0.92 (0.80;1.06) | 0.92 (0.81;1.05) | **1.18 (1.05;1.33)** | 1.11 (0.98;1.27) | 1.02 (0.87;1.19) | 1.06 (0.96;1.18) | 0.91 (0.77;1.08) | 1.02 (0.96;1.09) | **1.24 (1.04;1.48)** | 1.04 (0.98;1.11) | 0.93 (0.73;1.18) |
| **Ozone**  **(O_3_)** | **Lag 0** | 1.05 (0.93;1.19) | 0.99 (0.86;1.14) | 1.12 (0.98;1.27) | 0.92 (0.81;1.03) | 0.97 (0.79;1.19) | 0.93 (0.78;1.11) | 1.10 (0.95;1.26) | 0.95 (0.78;1.18) | 1.01 (0.93;1.11) | 1.01 (0.78;1.31) | 1.01 (0.92;1.10) | 1.05 (0.77;1.45) |
|  | **Lag 1** | 0.98 (0.87;1.10) | 1.05 (0.92;1.21) | 1.04 (0.91;1.19) | 0.90 (0.81;1.01) | 0.97 (0.80;1.17) | 0.99 (0.86;1.15) | 1.13 (0.99;1.29) | 0.92 (0.76;1.12) | 1.02 (0.94;1.11) | 1.06 (0.83;1.36) | 1.03 (0.94;1.11) | 1.08 (0.80;1.46) |
|  | **Lag 2** | 1.01 (0.90;1.14) | 0.98 (0.85;1.12) | 1.04 (0.91;1.19) | **0.83 (0.74;0.94)** | 0.98 (0.81;1.18) | 1.00 (0.87;1.16) | 1.12 (0.98;1.29) | 0.94 (0.77;1.14) | 1.04 (0.95;1.13) | 1.04 (0.82;1.33) | 1.03 (0.95;1.12) | 1.08 (0.80;1.47) |
|  | **Lag 3** | 1.09 (0.97;1.23) | 0.94 (0.81;1.08) | 1.09 (0.95;1.25) | **0.88 (0.78;0.99)** | 0.97 (0.80;1.17) | 1.01 (0.87;1.17) | 1.12 (0.98;1.28) | 0.94 (0.77;1.14) | 1.04 (0.95;1.13) | 1.03 (0.81;1.32) | 1.03 (0.95;1.12) | 1.10 (0.82;1.49) |
|  | **Lag 4** | 1.05 (0.93;1.18) | 0.99 (0.86;1.15) | **1.15 (1.00;1.33)** | **0.81 (0.72;0.91)** | 0.99 (0.82;1.19) | 1.00 (0.87;1.16) | 1.12 (0.98;1.28) | 0.93 (0.77;1.14) | 1.03 (0.95;1.12) | 1.04 (0.82;1.33) | 1.03 (0.95;1.12) | 1.08 (0.80;1.46) |
|  | **Lag 5** | 1.13 (1.01;1.27) | 0.92 (0.80;1.06) | 1.06 (0.92;1.22) | **0.87 (0.77;0.97)** | 0.98 (0.82;1.18) | 1.01 (0.88;1.17) | 1.12 (0.98;1.28) | 0.94 (0.78;1.15) | 1.03 (0.95;1.12) | 1.03 (0.81;1.32) | 1.03 (0.95;1.12) | 1.08 (0.80;1.46) |
|  | **Lag 6** | 1.08 (0.96;1.22) | 0.98 (0.85;1.13) | 0.99 (0.86;1.14) | 0.93 (0.83;1.05) | 0.98 (0.81;1.18) | 1.01 (0.88;1.17) | 1.12 (0.98;1.28) | 0.95 (0.78;1.16) | 1.03 (0.95;1.12) | 1.04 (0.82;1.33) | 1.03 (0.95;1.12) | 1.07 (0.79;1.45) |
| **Fine Particulate Matter**  **(PM_2.5_)** | **Lag 0** | **1.15 (1.04;1.28)** | 0.99 (0.88;1.11) | **0.91 (0.82;1.00)** | 1.03 (0.93;1.14) | 1.07 (0.94;1.21) | 1.04 (0.92;1.18) | 0.96 (0.87;1.07) | 0.96 (0.85;1.08) | 0.99 (0.92;1.05) | 1.13 (0.96;1.32) | 1.01 (0.95;1.08) | 0.89 (0.73;1.08) |
|  | **Lag 1** | 1.05 (0.94;1.16) | 0.96 (0.86;1.08) | 0.95 (0.86;1.05) | 1.07 (0.97;1.19) | 1.11 (0.98;1.26) | 0.99 (0.88;1.12) | 1.01 (0.91;1.12) | 0.97 (0.86;1.09) | 0.99 (0.93;1.06) | **1.19 (1.02;1.40)** | 1.02 (0.96;1.09) | 0.90 (0.75;1.09) |
|  | **Lag 2** | 1.05 (0.94;1.18) | 0.90 (0.79;1.01) | 0.99 (0.90;1.09) | 1.05 (0.95;1.16) | 1.11 (0.98;1.25) | 1.00 (0.89;1.12) | 1.01 (0.91;1.11) | 0.95 (0.85;1.07) | 1.00 (0.94;1.06) | 1.12 (0.96;1.30) | 1.02 (0.96;1.08) | 0.91 (0.76;1.09) |
|  | **Lag 3** | 0.91 (0.81;1.02) | 0.93 (0.83;1.05) | 1.01 (0.92;1.12) | 1.10 (0.99;1.21) | 1.12 (0.99;1.26) | 1.00 (0.89;1.12) | 1.01 (0.92;1.11) | 0.96 (0.86;1.08) | 1.00 (0.94;1.07) | 1.11 (0.95;1.29) | 1.02 (0.96;1.08) | 0.92 (0.77;1.11) |
|  | **Lag 4** | 1.07 (0.96;1.18) | 0.93 (0.82;1.06) | 0.94 (0.85;1.04) | **1.10 (1.00;1.21)** | 1.12 (0.99;1.26) | 1.00 (0.89;1.12) | 1.01 (0.92;1.11) | 0.95 (0.85;1.07) | 1.00 (0.94;1.06) | 1.11 (0.96;1.30) | 1.02 (0.96;1.08) | 0.91 (0.77;1.09) |
|  | **Lag 5** | **1.15 (1.03;1.29)** | 0.92 (0.81;1.04) | **0.89 (0.81;0.98)** | **1.17 (1.07;1.29)** | 1.11 (0.99;1.26) | 1.00 (0.90;1.12) | 1.01 (0.92;1.11) | 0.95 (0.85;1.06) | 1.00 (0.94;1.06) | 1.12 (0.96;1.30) | 1.02 (0.96;1.08) | 0.90 (0.75;1.07) |
|  | **Lag 6** | 1.05 (0.95;1.17) | 0.92 (0.82;1.04) | **0.89 (0.80;0.98)** | **1.12 (1.02;1.22)** | **1.12 (1.00;1.27)** | 1.01 (0.90;1.13) | 1.01 (0.92;1.11) | 0.95 (0.85;1.06) | 1.00 (0.94;1.06) | 1.13 (0.97;1.31) | 1.03 (0.97;1.09) | 0.90 (0.75;1.07) |
| **Maximum Temperature** | **Lag 0** | 0.83 (0.68;1.01) | **1.57 (1.32;1.88)** | **1.49 (1.19;1.86)** | 0.99 (0.84;1.18) | 0.79 (0.48;1.30) | 1.33 (0.81;2.18) | 1.13 (0.84;1.51) | 0.84 (0.51;1.38) | 1.02 (0.82;1.28) | 1.22 (0.65;2.31) | 1.05 (0.85;1.31) | 0.93 (0.41;2.10) |
|  | **Lag 1** | **0.82 (0.68;1.00)** | **1.68 (1.41;2.01)** | **1.55 (1.23;1.95)** | 1.02 (0.87;1.20) | 0.92 (0.62;1.36) | 1.11 (0.74;1.67) | 1.17 (0.91;1.51) | 0.84 (0.57;1.24) | 1.02 (0.85;1.22) | 1.22 (0.72;2.06) | 1.05 (0.88;1.26) | 0.90 (0.49;1.65) |
|  | **Lag 2** | **0.76 (0.62;0.92)** | **1.61 (1.35;1.92)** | **1.31 (1.05;1.65)** | 0.95 (0.81;1.12) | 0.96 (0.65;1.42) | 1.21 (0.81;1.82) | 1.17 (0.91;1.50) | 0.87 (0.59;1.27) | 1.06 (0.88;1.27) | 1.18 (0.70;1.99) | 1.09 (0.91;1.30) | 0.84 (0.46;1.54) |
|  | **Lag 3** | **0.75 (0.62;0.91)** | **1.42 (1.19;1.69)** | **1.35 (1.07;1.70)** | 0.97 (0.83;1.14) | 0.94 (0.64;1.40) | 1.25 (0.84;1.86) | 1.18 (0.92;1.51) | 0.87 (0.59;1.28) | 1.06 (0.89;1.27) | 1.14 (0.68;1.92) | 1.09 (0.91;1.30) | 0.88 (0.48;1.61) |
|  | **Lag 4** | **0.74 (0.61;0.90)** | **1.47 (1.22;1.76)** | **1.28 (1.02;1.61)** | 0.93 (0.80;1.10) | 0.97 (0.66;1.44) | 1.26 (0.85;1.89) | 1.17 (0.92;1.51) | 0.84 (0.57;1.23) | 1.05 (0.88;1.26) | 1.19 (0.71;2.00) | 1.08 (0.91;1.29) | 0.89 (0.48;1.63) |
|  | **Lag 5** | **0.71 (0.58;0.86)** | **1.49 (1.23;1.80)** | **1.28 (1.02;1.60)** | 0.94 (0.79;1.11) | 0.96 (0.65;1.41) | 1.27 (0.85;1.89) | 1.17 (0.91;1.50) | 0.83 (0.56;1.22) | 1.06 (0.88;1.27) | 1.16 (0.68;1.95) | 1.08 (0.91;1.29) | 0.89 (0.49;1.62) |
|  | **Lag 6** | **0.72 (0.59;0.89)** | **1.31 (1.09;1.57)** | **1.20 (0.95;1.51)** | 1.10 (0.93;1.30) | 0.95 (0.64;1.40) | 1.23 (0.82;1.83) | 1.16 (0.90;1.48) | 0.80 (0.55;1.18) | 1.05 (0.88;1.26) | 1.14 (0.68;1.92) | 1.07 (0.90;1.28) | 0.85 (0.46;1.56) |
| **Atmospheric Pressure** | **Lag 0** | 1.33 (0.85;2.07) | 1.00 (0.53;1.87) | 0.68 (0.45;1.03) | 1.15 (0.79;1.70) | 0.96 (0.82;1.13) | 1.10 (0.80;1.51) | 0.96 (0.72;1.29) | 0.61 (0.10;3.85) | 0.98 (0.79;1.23) | 0.91 (0.47;1.76) | 1.00 (0.83;1.20) | 0.36 (0.03;4.95) |
|  | **Lag 1** | 1.11 (0.70;1.76) | 1.00 (0.54;1.87) | 0.83 (0.55;1.24) | 1.22 (0.83;1.78) | 1.09 (0.93;1.28) | 1.13 (0.82;1.54) | 0.86 (0.64;1.16) | 1.35 (0.25;7.37) | 1.03 (0.82;1.28) | 1.09 (0.55;2.17) | 1.03 (0.86;1.24) | 0.61 (0.05;7.62) |
|  | **Lag 2** | 0.98 (0.62;1.53) | 1.65 (0.88;3.09) | 0.86 (0.58;1.27) | 0.95 (0.64;1.39) | 0.93 (0.79;1.09) | 1.23 (0.89;1.69) | 0.85 (0.64;1.13) | 1.17 (0.20;6.80) | 0.92 (0.74;1.14) | 1.39 (0.71;2.74) | 0.96 (0.80;1.16) | 0.93 (0.07;12.3) |
|  | **Lag 3** | 1.08 (0.68;1.70) | 1.94 (1.04;3.64) | **0.55 (0.37;0.82)** | 0.97 (0.66;1.42) | 0.95 (0.80;1.12) | 0.98 (0.71;1.34) | 0.97 (0.73;1.30) | 0.51 (0.08;3.19) | 0.89 (0.72;1.11) | 0.76 (0.38;1.54) | 0.90 (0.75;1.09) | 0.52 (0.03;8.42) |
|  | **Lag 4** | 0.94 (0.60;1.48) | 1.53 (0.82;2.85) | **0.56 (0.38;0.83)** | **1.50 (1.03;2.19)** | 0.95 (0.81;1.12) | 1.09 (0.80;1.48) | 1.03 (0.77;1.38) | 0.91 (0.16;5.15) | 0.98 (0.79;1.22) | 0.67 (0.35;1.31) | 0.95 (0.79;1.15) | 1.15 (0.09;15.4) |
|  | **Lag 5** | 0.74 (0.48;1.14) | 1.61 (0.85;3.05) | **0.63 (0.42;0.96)** | 1.07 (0.74;1.55) | 0.96 (0.81;1.13) | 0.99 (0.73;1.34) | 0.89 (0.67;1.18) | 0.93 (0.18;4.91) | 0.90 (0.73;1.12) | 0.65 (0.33;1.28) | 0.88 (0.74;1.06) | 1.43 (0.12;17.3) |
|  | **Lag 6** | 0.59 (0.38;0.93) | 1.61 (0.86;3.04) | **0.57 (0.38;0.87)** | **1.71 (1.19;2.46)** | 0.98 (0.83;1.14) | 1.09 (0.80;1.47) | 0.99 (0.75;1.32) | 1.26 (0.22;7.14) | 0.99 (0.80;1.22) | 1.09 (0.55;2.16) | 1.02 (0.85;1.23) | 0.42 (0.03;5.20) |

*Air pollution models (PM_2.5_, NO_2_, O_3_) were adjusted for maximum temperature, precipitation (mm/day), and shortwave solar radiation (W/m²). Maximum temperature models were adjusted for PM_2.5_, precipitation, and solar radiation. Atmospheric pressure models were adjusted for PM_2.5_, maximum temperature, precipitation, and solar radiation. Results are expressed as adjusted odds ratios (ORs) calculated per interquartile range (IQR) increase of each exposure.*

**Table S9:** Adjusted odds ratios (ORs) and 95% confidence intervals (CIs) for exposures across *single lag days* (0 to 6) from *opioid overdose*-stratified models, presented by season, region, rural/urban classification, and elevation. Estimates with CIs that do not cross 1 are highlighted in bold.

|  |  |  | | | | **Opioid Overdose** | | | |  | | | |
| --- | --- | --- | --- | --- | --- | --- | --- | --- | --- | --- | --- | --- | --- |
|  |  | **Season** | | | | **Region** | | | | **Urbanicity** | | **Altitude** | |
|  |  | **Spring** | **Summer** | **Fall** | **Winter** | **North Central** | **Northeast** | **South** | **West** | **Metro** | **Nonmetro** | **< 500 m** | **>= 500 m** |
| **Nitrogen Dioxide**  **(NO_2_)** | **Lag 0** | 1.04 (0.89;1.21) | 0.88 (0.73;1.05) | 0.93 (0.79;1.10) | 1.06 (0.92;1.22) | 0.99 (0.84;1.17) | 1.04 (0.88;1.24) | 1.03 (0.89;1.18) | 0.99 (0.76;1.27) | 1.00 (0.92;1.09) | 1.20 (0.96;1.52) | 1.02 (0.94;1.10) | 0.85 (0.57;1.26) |
|  | **Lag 1** | 0.92 (0.79;1.08) | 0.93 (0.79;1.10) | 0.98 (0.83;1.16) | 1.12 (0.97;1.29) | 1.02 (0.87;1.21) | 1.03 (0.87;1.23) | 1.06 (0.92;1.21) | 1.00 (0.79;1.27) | 1.01 (0.93;1.10) | 1.23 (0.98;1.54) | 1.03 (0.95;1.12) | 0.90 (0.62;1.32) |
|  | **Lag 2** | 1.09 (0.93;1.28) | 1.05 (0.87;1.27) | 1.02 (0.87;1.21) | 1.03 (0.89;1.19) | 1.01 (0.86;1.19) | 1.02 (0.86;1.22) | 1.05 (0.91;1.20) | 1.00 (0.78;1.27) | 1.01 (0.93;1.10) | 1.20 (0.95;1.51) | 1.03 (0.95;1.11) | 0.90 (0.61;1.31) |
|  | **Lag 3** | 0.87 (0.75;1.01) | 0.97 (0.81;1.15) | 1.05 (0.90;1.23) | 1.09 (0.94;1.26) | 1.01 (0.86;1.19) | 1.04 (0.87;1.23) | 1.05 (0.91;1.20) | 1.01 (0.80;1.29) | 1.02 (0.94;1.11) | 1.20 (0.96;1.51) | 1.03 (0.95;1.12) | 0.88 (0.60;1.30) |
|  | **Lag 4** | 0.98 (0.85;1.14) | 0.93 (0.78;1.11) | 1.03 (0.87;1.21) | 1.15 (0.99;1.34) | 1.03 (0.88;1.21) | 1.03 (0.87;1.23) | 1.05 (0.91;1.20) | 0.99 (0.78;1.26) | 1.02 (0.94;1.10) | 1.18 (0.94;1.49) | 1.03 (0.95;1.12) | 0.92 (0.63;1.34) |
|  | **Lag 5** | 1.00 (0.86;1.15) | 0.91 (0.77;1.07) | 1.00 (0.85;1.17) | 1.07 (0.93;1.24) | 1.04 (0.89;1.23) | 1.04 (0.87;1.23) | 1.05 (0.92;1.20) | 0.97 (0.76;1.24) | 1.02 (0.94;1.10) | 1.21 (0.96;1.52) | 1.03 (0.95;1.12) | 0.91 (0.62;1.33) |
|  | **Lag 6** | 1.00 (0.86;1.16) | 0.90 (0.76;1.07) | 0.97 (0.83;1.13) | **1.24 (1.07;1.43)** | 1.06 (0.90;1.24) | 1.03 (0.87;1.22) | 1.06 (0.92;1.21) | 0.97 (0.76;1.23) | 1.02 (0.94;1.11) | 1.21 (0.96;1.52) | 1.04 (0.96;1.13) | 0.87 (0.60;1.27) |
| **Ozone**  **(O_3_)** | **Lag 0** | 1.09 (0.93;1.28) | 0.95 (0.80;1.13) | 1.12 (0.96;1.31) | 0.90 (0.77;1.04) | 0.99 (0.77;1.26) | 0.93 (0.76;1.14) | 1.08 (0.90;1.30) | 1.15 (0.84;1.58) | 1.00 (0.90;1.12) | 1.11 (0.77;1.60) | 1.00 (0.90;1.12) | 1.26 (0.81;1.97) |
|  | **Lag 1** | 0.89 (0.76;1.04) | 1.06 (0.89;1.27) | 1.01 (0.85;1.19) | 0.90 (0.79;1.03) | 0.99 (0.79;1.24) | 0.97 (0.82;1.15) | 1.11 (0.93;1.31) | 1.13 (0.84;1.52) | 1.02 (0.92;1.14) | 1.22 (0.86;1.74) | 1.03 (0.93;1.14) | 1.29 (0.85;1.98) |
|  | **Lag 2** | 1.04 (0.89;1.21) | 1.06 (0.89;1.26) | 1.01 (0.85;1.19) | **0.80 (0.69;0.93)** | 1.01 (0.81;1.26) | 0.99 (0.84;1.17) | 1.10 (0.93;1.31) | 1.08 (0.80;1.44) | 1.04 (0.94;1.15) | 1.19 (0.84;1.69) | 1.04 (0.94;1.15) | 1.27 (0.83;1.94) |
|  | **Lag 3** | 1.15 (0.99;1.35) | 1.04 (0.87;1.24) | 1.11 (0.94;1.32) | 0.91 (0.79;1.06) | 0.99 (0.79;1.23) | 1.00 (0.85;1.18) | 1.10 (0.92;1.30) | 1.08 (0.80;1.45) | 1.04 (0.94;1.15) | 1.15 (0.81;1.63) | 1.03 (0.93;1.14) | 1.30 (0.85;1.99) |
|  | **Lag 4** | 1.04 (0.89;1.20) | 1.02 (0.85;1.22) | **1.23 (1.03;1.47)** | **0.81 (0.71;0.93)** | 1.01 (0.81;1.26) | 1.00 (0.85;1.18) | 1.10 (0.93;1.30) | 1.09 (0.81;1.46) | 1.04 (0.94;1.15) | 1.18 (0.83;1.67) | 1.03 (0.93;1.14) | 1.29 (0.85;1.96) |
|  | **Lag 5** | 1.09 (0.94;1.26) | 0.91 (0.77;1.08) | 1.18 (0.99;1.41) | **0.85 (0.73;0.97)** | 1.01 (0.81;1.25) | 1.00 (0.85;1.19) | 1.10 (0.92;1.30) | 1.14 (0.85;1.53) | 1.04 (0.94;1.15) | 1.13 (0.80;1.61) | 1.03 (0.93;1.14) | 1.34 (0.88;2.06) |
|  | **Lag 6** | 1.03 (0.89;1.20) | 0.96 (0.80;1.15) | 0.99 (0.83;1.18) | 0.91 (0.79;1.05) | 1.01 (0.81;1.26) | 1.00 (0.85;1.18) | 1.10 (0.92;1.30) | 1.13 (0.84;1.51) | 1.04 (0.94;1.15) | 1.16 (0.82;1.65) | 1.04 (0.94;1.14) | 1.21 (0.79;1.84) |
| **Fine Particulate Matter**  **(PM_2.5_)** | **Lag 0** | 1.11 (0.97;1.27) | 0.93 (0.80;1.09) | **0.88 (0.77;1.00)** | 1.04 (0.92;1.17) | 1.03 (0.88;1.20) | 1.08 (0.93;1.24) | 0.90 (0.79;1.03) | 0.97 (0.81;1.16) | 0.98 (0.90;1.06) | 1.12 (0.88;1.44) | 1.00 (0.92;1.08) | 0.80 (0.60;1.07) |
|  | **Lag 1** | 0.98 (0.85;1.12) | 0.93 (0.80;1.08) | 0.93 (0.82;1.05) | 1.11 (0.99;1.25) | 1.08 (0.92;1.26) | 1.02 (0.89;1.18) | 0.94 (0.83;1.07) | 0.98 (0.83;1.17) | 0.98 (0.91;1.07) | **1.29 (1.00;1.65)** | 1.01 (0.94;1.09) | 0.84 (0.64;1.10) |
|  | **Lag 2** | 1.09 (0.94;1.26) | 0.96 (0.82;1.11) | 0.98 (0.86;1.11) | 1.04 (0.93;1.17) | 1.07 (0.92;1.24) | 1.02 (0.90;1.17) | 0.94 (0.83;1.06) | 0.97 (0.82;1.15) | 0.99 (0.92;1.07) | 1.09 (0.86;1.38) | 1.01 (0.94;1.08) | 0.87 (0.67;1.12) |
|  | **Lag 3** | 0.93 (0.81;1.07) | 1.05 (0.90;1.22) | 1.00 (0.88;1.14) | **1.12 (1.00;1.25)** | 1.08 (0.93;1.25) | 1.03 (0.90;1.18) | 0.95 (0.84;1.07) | 0.99 (0.84;1.18) | 1.00 (0.93;1.08) | 1.08 (0.86;1.36) | 1.01 (0.94;1.09) | 0.89 (0.69;1.15) |
|  | **Lag 4** | 1.10 (0.97;1.25) | 0.97 (0.82;1.13) | 0.96 (0.85;1.08) | **1.13 (1.01;1.27)** | 1.08 (0.94;1.26) | 1.03 (0.91;1.18) | 0.95 (0.84;1.07) | 0.98 (0.82;1.16) | 1.00 (0.93;1.08) | 1.09 (0.87;1.37) | 1.01 (0.94;1.09) | 0.90 (0.70;1.15) |
|  | **Lag 5** | 1.08 (0.93;1.24) | 0.97 (0.83;1.12) | 0.89 (0.79;1.01) | **1.23 (1.10;1.37)** | 1.09 (0.94;1.26) | 1.04 (0.91;1.18) | 0.95 (0.85;1.07) | 0.98 (0.82;1.16) | 1.00 (0.93;1.07) | 1.12 (0.89;1.41) | 1.02 (0.95;1.09) | 0.90 (0.70;1.15) |
|  | **Lag 6** | 0.96 (0.85;1.09) | 0.98 (0.84;1.14) | 0.91 (0.81;1.02) | **1.19 (1.07;1.33)** | 1.10 (0.95;1.28) | 1.04 (0.92;1.19) | 0.95 (0.85;1.07) | 0.98 (0.82;1.16) | 1.00 (0.93;1.08) | 1.11 (0.88;1.40) | 1.02 (0.95;1.10) | 0.89 (0.69;1.14) |
| **Maximum Temperature** | **Lag 0** | 0.82 (0.64;1.06) | **1.50 (1.21;1.87)** | **1.77 (1.33;2.36)** | 0.94 (0.78;1.15) | 0.74 (0.40;1.35) | 1.18 (0.67;2.09) | 1.36 (0.93;2.00) | 0.88 (0.42;1.83) | 1.03 (0.78;1.37) | 1.69 (0.68;4.22) | 1.08 (0.82;1.43) | 0.92 (0.31;2.77) |
|  | **Lag 1** | 0.87 (0.68;1.11) | **1.56 (1.26;1.93)** | **1.76 (1.32;2.35)** | 0.97 (0.80;1.17) | 0.95 (0.59;1.55) | 1.13 (0.71;1.79) | 1.31 (0.94;1.82) | 1.07 (0.61;1.89) | 1.07 (0.85;1.35) | 1.81 (0.85;3.85) | 1.11 (0.89;1.40) | 1.07 (0.46;2.48) |
|  | **Lag 2** | 0.80 (0.62;1.02) | **1.44 (1.16;1.79)** | **1.46 (1.10;1.95)** | 0.90 (0.74;1.09) | 0.96 (0.59;1.54) | 1.18 (0.75;1.88) | 1.33 (0.96;1.85) | 1.00 (0.57;1.75) | 1.11 (0.88;1.39) | 1.57 (0.75;3.30) | 1.14 (0.91;1.44) | 0.96 (0.42;2.18) |
|  | **Lag 3** | 0.84 (0.65;1.08) | 1.24 (0.99;1.54) | **1.48 (1.11;1.98)** | 0.96 (0.79;1.15) | 0.92 (0.57;1.48) | 1.26 (0.80;1.98) | 1.34 (0.97;1.85) | 1.03 (0.59;1.81) | 1.13 (0.90;1.42) | 1.46 (0.69;3.06) | 1.16 (0.92;1.45) | 0.98 (0.43;2.25) |
|  | **Lag 4** | 0.80 (0.62;1.03) | **1.50 (1.19;1.88)** | **1.44 (1.08;1.92)** | 0.94 (0.78;1.14) | 0.96 (0.60;1.55) | 1.26 (0.80;1.99) | 1.33 (0.96;1.84) | 0.98 (0.56;1.72) | 1.12 (0.89;1.40) | 1.52 (0.72;3.22) | 1.15 (0.91;1.44) | 1.09 (0.48;2.50) |
|  | **Lag 5** | **0.77 (0.59;0.99)** | **1.59 (1.26;2.01)** | **1.43 (1.07;1.90)** | 0.97 (0.79;1.18) | 0.94 (0.58;1.51) | 1.27 (0.81;2.00) | 1.32 (0.96;1.83) | 1.01 (0.57;1.76) | 1.11 (0.89;1.40) | 1.47 (0.69;3.12) | 1.14 (0.91;1.43) | 1.11 (0.49;2.53) |
|  | **Lag 6** | 0.82 (0.63;1.06) | **1.33 (1.07;1.66)** | **1.37 (1.02;1.85)** | 1.10 (0.90;1.34) | 0.93 (0.58;1.50) | 1.24 (0.79;1.96) | 1.31 (0.95;1.80) | 0.97 (0.55;1.69) | 1.11 (0.88;1.39) | 1.42 (0.67;2.99) | 1.12 (0.90;1.41) | 1.07 (0.47;2.45) |
| **Atmospheric Pressure** | **Lag 0** | 1.25 (0.77;2.02) | 1.06 (0.52;2.19) | 0.75 (0.48;1.16) | 1.13 (0.74;1.71) | 0.91 (0.75;1.09) | 1.14 (0.81;1.60) | 1.11 (0.77;1.61) | 0.18 (0.01;3.91) | 1.02 (0.80;1.29) | 0.95 (0.41;2.19) | 1.02 (0.82;1.26) | 0.50 (0.01;17.2) |
|  | **Lag 1** | 1.14 (0.69;1.88) | 1.07 (0.53;2.17) | 0.94 (0.61;1.47) | 1.06 (0.70;1.59) | 0.99 (0.82;1.19) | 1.27 (0.90;1.78) | 0.91 (0.63;1.31) | 0.59 (0.03;11.91) | 1.04 (0.82;1.31) | 1.06 (0.43;2.57) | 1.02 (0.82;1.26) | 3.53 (0.10;121) |
|  | **Lag 2** | 0.82 (0.50;1.35) | 1.66 (0.81;3.39) | 0.83 (0.53;1.29) | 0.93 (0.62;1.40) | 0.85 (0.70;1.03) | 1.26 (0.89;1.78) | 0.76 (0.53;1.08) | 8.00 (0.35;180.7) | 0.88 (0.69;1.11) | 1.60 (0.68;3.80) | 0.91 (0.73;1.12) | 2.26 (0.07;73.6) |
|  | **Lag 3** | 1.15 (0.71;1.89) | 1.54 (0.75;3.16) | **0.55 (0.36;0.85)** | 0.93 (0.63;1.38) | 0.91 (0.75;1.10) | 0.99 (0.71;1.38) | 0.84 (0.59;1.20) | 2.12 (0.09;50.38) | 0.88 (0.70;1.11) | 0.58 (0.24;1.41) | 0.88 (0.71;1.08) | 0.17 (0.00;7.92) |
|  | **Lag 4** | 0.83 (0.51;1.36) | 1.14 (0.56;2.32) | **0.55 (0.36;0.84)** | 1.21 (0.81;1.79) | 0.89 (0.73;1.07) | 0.99 (0.72;1.36) | 0.86 (0.60;1.24) | 1.67 (0.09;29.71) | 0.87 (0.69;1.10) | 0.58 (0.26;1.30) | 0.85 (0.69;1.05) | 1.06 (0.03;38.2) |
|  | **Lag 5** | 0.68 (0.43;1.08) | 1.17 (0.58;2.37) | **0.60 (0.39;0.92)** | 1.02 (0.68;1.52) | 0.92 (0.77;1.12) | 0.88 (0.64;1.21) | 0.81 (0.57;1.15) | 2.89 (0.16;51.16) | 0.83 (0.66;1.04) | 0.60 (0.27;1.34) | 0.81 (0.66;1.00) | 1.10 (0.03;36.3) |
|  | **Lag 6** | **0.60 (0.36;0.98)** | 1.21 (0.59;2.49) | **0.46 (0.29;0.72)** | 1.66 (1.13;2.43) | 0.92 (0.77;1.09) | 1.00 (0.73;1.38) | 1.00 (0.70;1.41) | 1.54 (0.08;29.93) | 0.92 (0.73;1.16) | 0.87 (0.37;2.02) | 0.95 (0.77;1.17) | 0.22 (0.01;7.13) |

*Air pollution models (PM_2.5_, NO_2_, O_3_) were adjusted for maximum temperature, precipitation (mm/day), and shortwave solar radiation (W/m²). Maximum temperature models were adjusted for PM_2.5_, precipitation, and solar radiation. Atmospheric pressure models were adjusted for PM_2.5_, maximum temperature, precipitation, and solar radiation. Results are expressed as adjusted odds ratios (ORs) calculated per interquartile range (IQR) increase of each exposure.*
